# Iodine-mediated thio-arylation under electrochemical conditions†

**DOI:** 10.1039/d5ra00100e

**Published:** 2025-04-16

**Authors:** Jiajia Yu, Tong Li, Qi Sun, Zhiyong Wang

**Affiliations:** a Institute of Advanced Technology, University of Science and Technology of China Hefei 230000 China sunqi924@ustc.edu.cn zwang3@ustc.edu.cn; b Hefei National Research Center for Physical Sciences at Microscale, Key Laboratory of Precision and Intelligent Chemistry, School of Chemistry and Materials Science, University of Science and Technology of China Hefei 230026 China

## Abstract

An efficient iodine-catalyzed thio-arylation reaction of aniline was developed under electrochemical conditions. A variety of diaryl sulfide compounds can be obtained under metal-free and chemical oxidant-free conditions. The reaction features a broad substrate scope, regulation of product distribution, and scalable preparation.

## Abstract 2

An efficient iodine-mediated electrochemical construction of C–S bonds under metal-free and chemical oxidant-free conditions.

## Graphical abstract

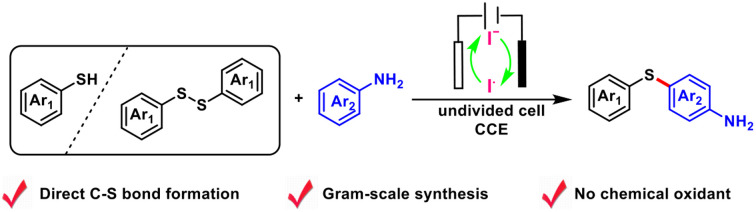

Sulfur-containing compounds play an important role in medicines,^1^ natural products,^2^ and functional materials.^3^ For instance, vortioxetine is a substance that regulates neurotransmitters and acts on multiple receptors, effectively alleviating depression-related symptoms. Dapson antibiotics have antibacterial and anti-inflammatory effects (Fig. 1).^4^ Owing to the excellent biological activity of sulfur-containing compounds, numerous methods have been proposed for synthesizing sulfur-containing compounds.

**Fig. 1:**
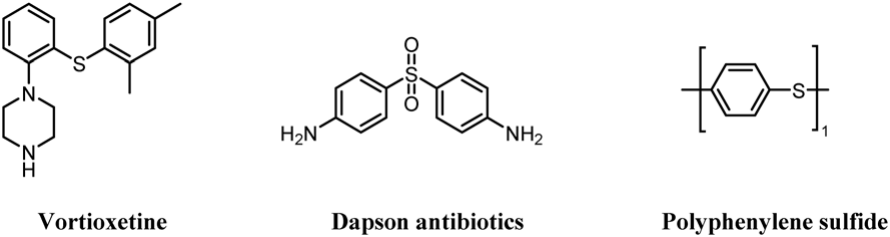
Organosulfur compounds in natural products and drugs.

At present, the conventional synthesis strategies for sulfur-containing compounds are mainly divided into two categories. One is the cross-coupling synthesis of thiols/disulfides and aryl halides in the presence of transition metal catalysts such as Pd,^5^ and Rh.^6^ The another one is the formation of diaryl sulfides in the presence of an I_2_/peroxide catalytic system.^7^ For example, the Schoenebeck group successfully constructed C–S bonds using a Pd catalyst.^8^ Wang's group reported the formation of diaryl sulfides using an I_2_/DTBP catalytic system.^9^ However, these traditional methods require the use of expensive substrates, chemical oxidants, metal catalysts, and harsh reaction conditions. However, green chemistry principles demand the development of facile synthesis strategies with atomic economy and environmental benignity. In this study, an electrochemical method was adopted, wherein only 1.0 equivalent of Et_4_NI was needed as the electrolyte, and the construction of C–S bonds was completed in 4 h. Compared with the I_2_/DTBP catalytic system, this reaction did not require the excess addition of peroxides (such as DTBP). Additionally, this electrochemical method could be carried out under air atmosphere, while the I_2_/DTBP catalysis required inert gas protection.

Electrochemical synthesis is an environmentally benign method to prepare organic compounds as electricity can provide the electrons needed for redox reactions, thereby avoiding the use of redox reagents.^10^ Previously, Zhou's group reported the construction of C–S bonds between quinoxalinone and thiol under electrochemical conditions.^11^ Intrigued by this electrochemical construction of diaryl sulfur compounds, we developed a reaction to directly construct aromatic sulfur bonds under electrochemical conditions, without the involvement of any transition metal catalysts or chemical oxidants (Scheme 1).

**Scheme 1:**
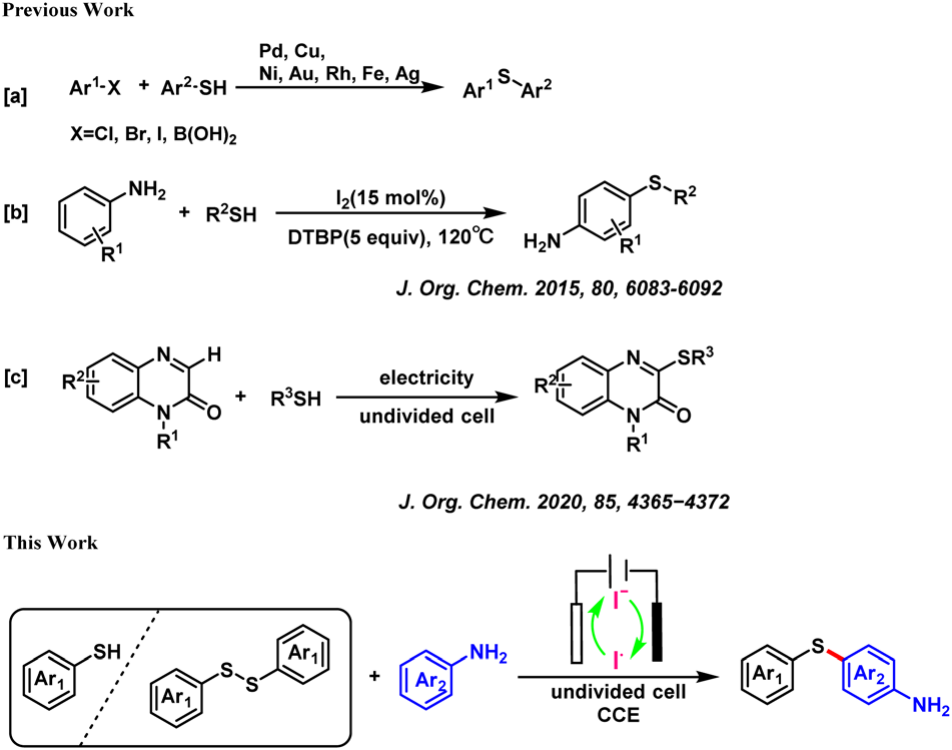
Previous work and this work for the synthesis of diaryl sulfides.

## Results and discussion

As a model reaction, the reaction of diphenyl disulfide 1a and aniline 2a was conducted in the presence of Et_4_NI as the electrolyte and dimethyl sulfoxide (DMSO) as the solvent at a constant current density of 10 mA cm^−2^ for 4 h under air atmosphere. The desired product was obtained with a high yield of 74% (entry 1, Table 1). Based on this result, we further optimized the reaction conditions. Firstly, different solvents were screened. The experimental results showed that DMSO was the optimal solvent, as shown in Table 1. Subsequently, various bases and acids were added to the reaction mixture, and the results demonstrated that these additions had a negative influence on the reaction, as shown in entries 4–8 of Table 1. To our delight, when H_2_O was added, the reaction yield was enhanced from 74% to 80% (entry 10, Table 1). The amount of H_2_O added was also investigated. We found that a ratio of DMSO : H_2_O = 10 : 1 gave the optimal result, affording 3aa in 86% yield (entry 11, Table 1). Afterwards, a variety of electrolytes, such as *n*-Bu_4_NBF_4_, *n*-Bu_4_NPF_6_, NH_4_BF_4_, were examined in this reaction. However, no better result was obtained (entries 13–15, Table 1). It was noted that the iodide ion in the electrolyte was essential for constructing the C(sp^2^)–S bond. In the absence of iodide in the electrolyte, no desired product was observed (entries 13–15, Table 1). As a result, we optimized different iodine salts and found that Et_4_NI was the best electrolyte for the reaction (entries 16–20, Table 1). Furthermore, the reaction was performed at different temperature. It was found that low yield of 40% was obtained when the reaction was carried out at 100 °C. At temperatures below 100 °C, it was challenging to obtain the desired product, as shown in entries 1–8 of Table S1 in the ESI.† In the end, the electrode was optimized. We attempted to use carbon electrodes to replace the platinum electrodes. However, carbon electrode gave poor results, as shown in entries 21–23 of Table 1.

**Table 1:** Optimization of reaction conditions^a^

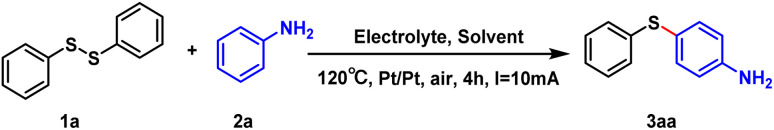					
Entry	Solvent	Additive	Electrolyte	Electrode	Yield^b^ [%]
1	DMSO	—	Et_4_NI	Pt(+)/Pt(−)	74
2	DMF	—	Et_4_NI	Pt(+)/Pt(−)	nd^d^
3	DMA	—	Et_4_NI	Pt(+)/Pt(−)	20
4	DMSO	DIPEA	Et_4_NI	Pt(+)/Pt(−)	nd^d^
5	DMSO	Cs_2_CO_3_	Et_4_NI	Pt(+)/Pt(−)	nd^d^
6	DMSO	NaOH	Et_4_NI	Pt(+)/Pt(−)	nd^d^
7	DMSO	H_2_SO_4_	Et_4_NI	Pt(+)/Pt(−)	nd^d^
8	DMSO	H_2_C_2_O_4_	Et_4_NI	Pt(+)/Pt(−)	nd^d^
9	DMSO	H_2_O	Et_4_NI	Pt(+)/Pt(−)	80
10	DMSO : H_2_O = 7 : 1	—	Et_4_NI	Pt(+)/Pt(−)	80
11	DMSO : H_2_O = 10 : 1	—	Et_4_NI	Pt(+)/Pt(−)	86
12	DMSO : H_2_O = 15 : 1	—	Et_4_NI	Pt(+)/Pt(−)	82
13	DMSO : H_2_O = 10 : 1	—	*n*-Bu_4_NBF_4_	Pt(+)/Pt(−)	nd^d^
14	DMSO : H_2_O = 10 : 1	—	*n*-Bu_4_NPF_6_	Pt(+)/Pt(−)	nd^d^
15	DMSO : H_2_O = 10 : 1	—	NH_4_BF_4_	Pt(+)/Pt(−)	nd^d^
16	DMSO : H_2_O = 10 : 1	—	*n*-Bu_4_NI	Pt(+)/Pt(−)	55
17	DMSO : H_2_O = 10 : 1	—	Me_4_NI	Pt(+)/Pt(−)	66
18	DMSO : H_2_O = 10 : 1	—	NH_4_I	Pt(+)/Pt(−)	65
19	DMSO : H_2_O = 10 : 1	—	KI	Pt(+)/Pt(−)	60
20	DMSO : H_2_O = 10 : 1	—	NaI	Pt(+)/Pt(−)	40
21	DMSO : H_2_O = 10 : 1	—	Et_4_NI	C(+)/C(−)	30
22	DMSO : H_2_O = 10 : 1	—	Et_4_NI	Pt(+)/C(−)	65
23	DMSO : H_2_O = 10 : 1	—	Et_4_NI	C(+)/Pt(−)	70
24^c^	DMSO : H_2_O = 10 : 1	—	Et_4_NI	—	nd^d^

a Standard conditions: platinum plate (10 mm × 10 mm × 0.2 mm) as the anode, platinum plate (10 mm × 10 mm × 0.2 mm) as the cathode, undivided cell, 1a (0.15 mmol), 2a (0.9 mmol), Et_4_NI (0.3 mmol), and DMSO (3 mL), air, 120 °C, 4 h.

b Isolated yield.

c Without electricity.

d Not detected.

**Scheme 2:**
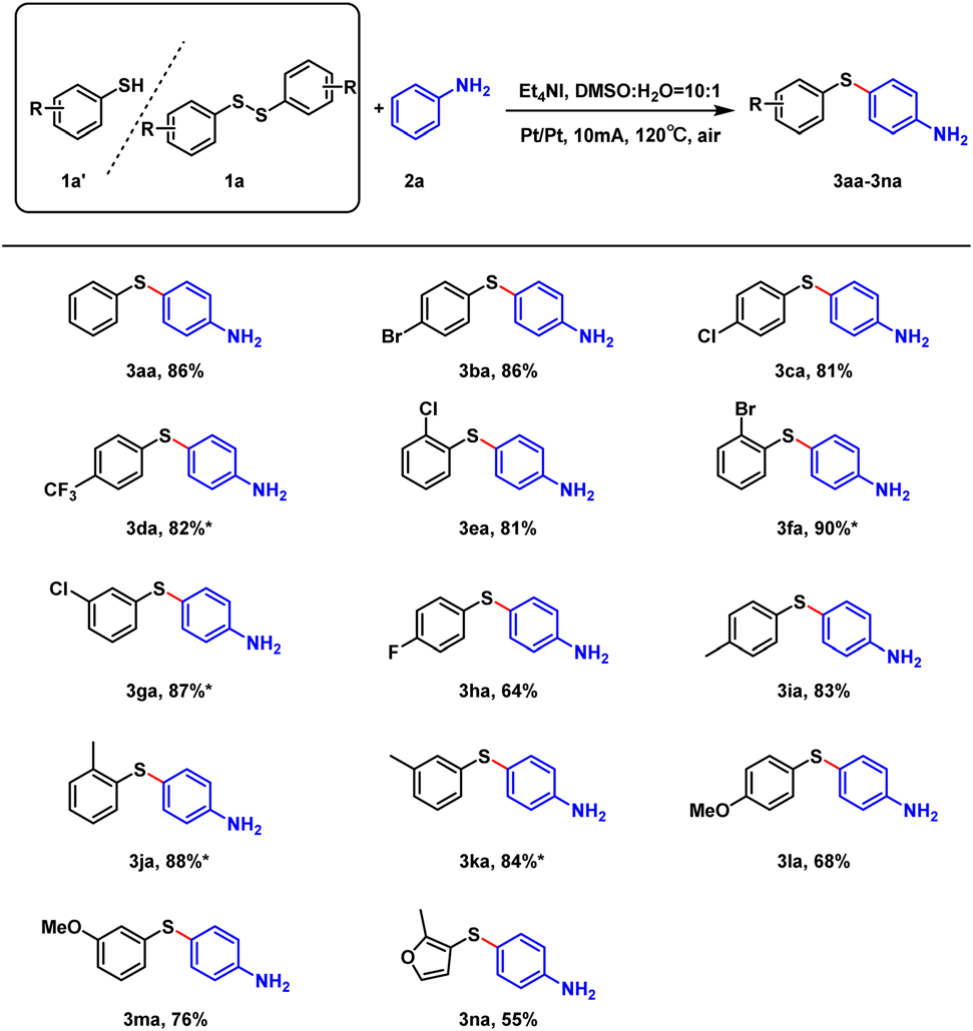
Scope of substrates. Unless otherwise noted, all reactions were performed with 1a–1n (0.15 mmol), 2a (0.9 mmol), Et_4_NI (0.3 mmol), DMSO (3.0 mL). The reaction was carried out at 120 °C for 4 h. *Reaction conditions: thiophenols as substrate 1d′, 1f′, 1g′, 1j′, 1k′ (0.3 mmol), 2a (0.9 mmol), Et_4_NI (0.3 mmol), DMSO (3.0 mL). The reaction was carried out at 120 °C for 4 h.

After establishing the optimal conditions, the substrate scope of thiols and thioethers was studied. As show in Scheme 2, the reaction yields ranged from 81% to 90% (3ba–3ga) when the substituent was an electron-withdrawing group. The position of the methyl substituent had no significant effect on the reaction, and the yield was generally above 83% (3ia–3ka). When the substituent was a strong electron-donating methoxy group, the yield was significantly reduced (3la–3ma). More importantly, when 4-(trifluoromethyl)thiophenol (1d′), 2-bromothiophenol (1f′), 3-chlorothiophenol (1g′), 2-methylbenzenethiol (1j′) and 3-methylbenzenethiol (1k′) were used as substrates, the reactions proceeded smoothly, and the desired products were obtained with yields of 82–90%. In addition, bis(2-methyl-3-furyl) disulfide was also well tolerated and generated the desired product with 55% yield (3na).

**Scheme 3:**
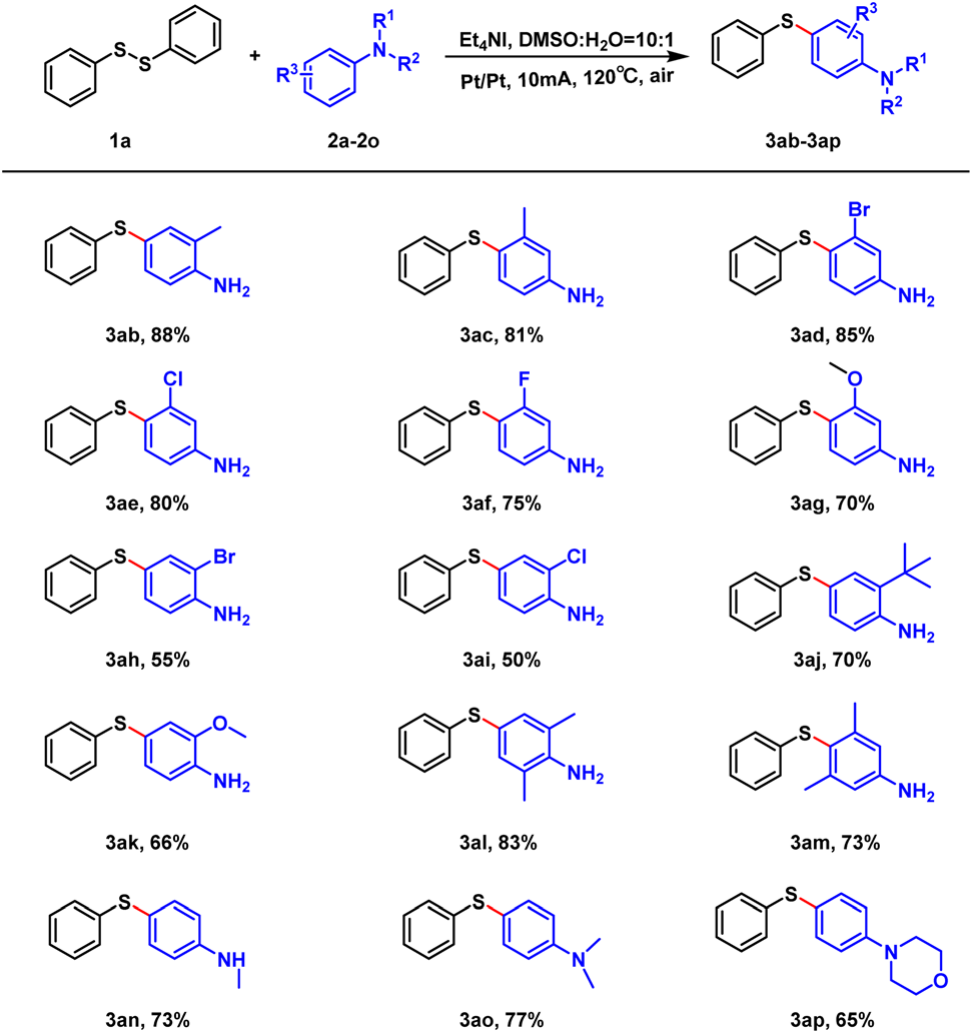
Scope of substrates. Unless otherwise noted, all reactions were performed with 1a (0.15 mmol), 2a–2p (0.9 mmol), Et_4_NI (0.3 mmol), DMSO (3.0 mL). The reaction was carried out at 120 °C for 4 h.

Afterwards, we examined the range of aniline substrates. As shown in Scheme 3, the reaction yields were above 81% (3ab, 3ac) when the single methyl substituent was present on the phenyl ring, regardless of the substituent position. Regarding other substituents, the electronic effect influenced the reaction. When the substituent was located at the *meta*-position, the electron-withdrawing group favored the reaction, while the electron-donating group disfavored it (3ad–3ag). In contrast, when the substituent was located at the *ortho*-position, the electron-donating group was superior to the electron-withdrawing group (3ah–3ak). Regarding di-substitution, the steric effect influenced the reaction (3al, 3am). When the hydrogen on the amino group of the aniline was replaced, the moderate yields of 65–70% were obtained (3an–3ap).

**Scheme 4:**
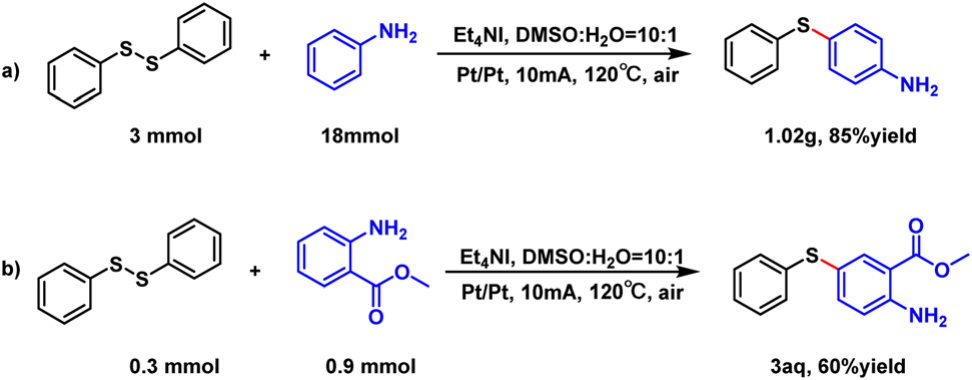
Gram-scale experiments, and their employment in the preparation of natural products.

In addition, we compared this electrochemical method with the chemical method. For instance, some substrates, such as 3aa, 3da, 3fa, 3ga, 3ka, 3ha and 3af, worked poorly in the I_2_/DTBP catalysis while worked well in this electrochemical reaction (see the control experiments on page S5 of the ESI†). To demonstrate the utility of this reaction, we performed the gram-scale of the model reaction. This scale-up reaction was carried out smoothly affording the desired product with a high yield of 85% (Scheme 4). Additionally, methyl *ortho*-aminobenzoate, an important pharmaceutical intermediate of drugs for psychotropic therapy and antimicrobial activity^12^ (3aq) was successfully prepared using our method in one step with a yield of 60%.

**Fig. 2:**
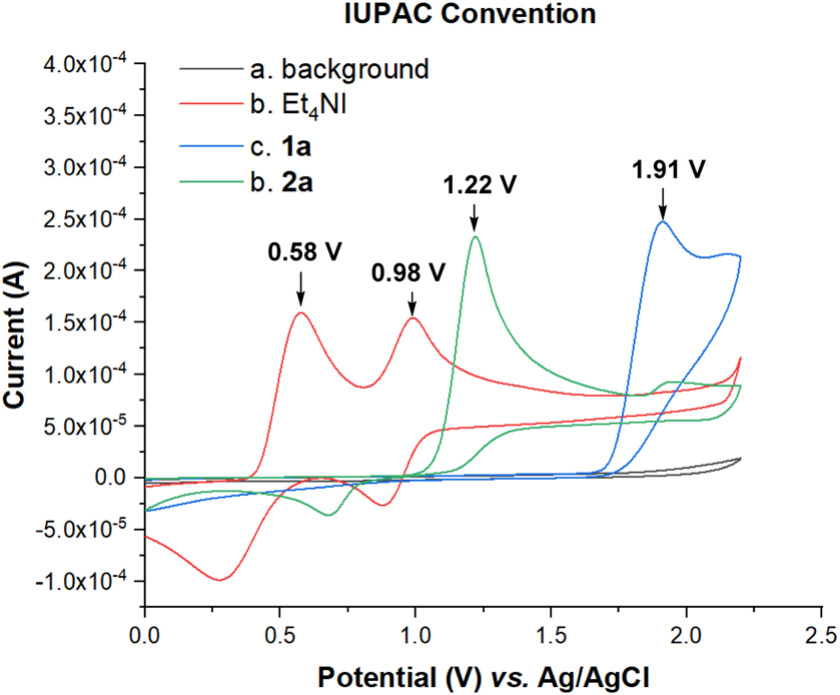
Cyclic voltammetry experiments: cyclic voltammograms of 1a, 2a and Et_4_NI in 0.1 M *n*-Bu_4_NBF_4_/MeCN = 10 mL using a Pt disk as the working electrode, and a Pt wire and Ag/AgCl as the counter and reference electrodes, respectively, at a scan rate of 100 mV s^−1^; background (curve *a*), Et_4_NI (5 mmol L^−1^, curve *b*), 1a (5 mmol L^−1^, curve *c*) and 2a (5 mmol L^−1^, curve *d*).

To gain more insights into the reaction mechanism, cyclic voltammetry (CV) experiments were conducted. The electrochemical properties of the starting materials were investigated, as shown in Fig. 2. The oxidation peaks from Et_4_NI (0.58 V, 0.98 V) [*vs.* Ag/AgCl] were observed, while the oxidation peaks of 1a (1.91 V) and 2a (1.22 V) were also detected. This observation verified the catalytic role of Et_4_NI in the reaction, indicating that the iodide anion should be oxidized first under standard conditions. We hypothesized that the iodide oxidation could generate some active species to initiate the reaction.

**Scheme 5:**
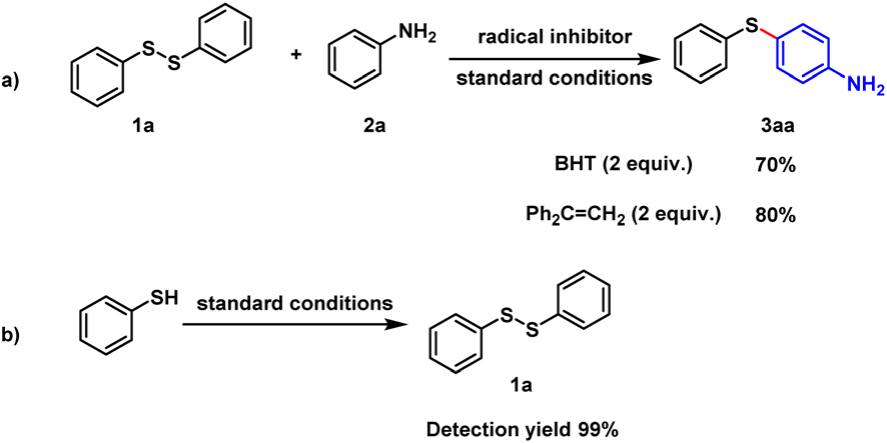
Control experiments.

To further investigate the reaction mechanism, we conducted a series of control experiments (Scheme 5). Initially, the reaction proceeded well in the presence of BHT (butylated hydroxytoluene) and 1,1-diphenylethylene (DPE), suggesting that the reaction may not proceed through a radical process. During the electrolysis process under standard conditions, the complete conversion of thiophenol to diphenyl disulfide was detected.

**Scheme 6:**
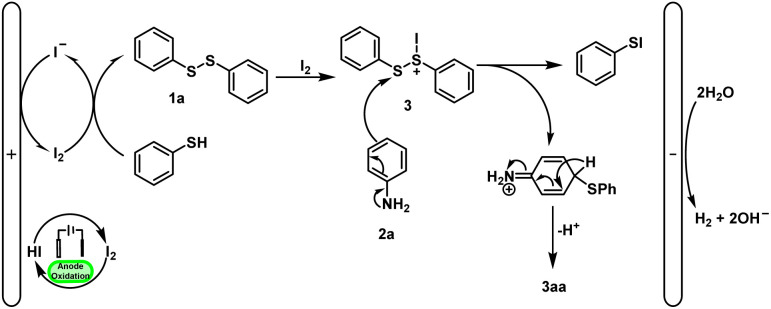
Proposed reaction mechanism.

Based on these control experiments and some previous literature reports,^7,10,13,14^ a relatively reasonable reaction mechanism was proposed (Scheme 6). First, iodine anions are oxidized to I_2_ at the anode, which can oxidize thiophenol to diphenyl disulfide. The diphenyl disulfide can be further converted into the cationic species 3 by molecular iodine. Subsequently, the cationic species 3 reacts with aniline to afford 3aa. Meanwhile, the water is reduced at the cathode.

## Conclusion

In summary, we developed a method to synthesize diaryl sulfide derivatives from aniline and thioether/thiophenol *via* electrochemical catalysis in the presence of Et_4_NI. This electrocatalytic C–H/S–H coupling reaction can be carried out smoothly without the use of metals or oxidants, under the air atmosphere. Furthermore, the reaction features a broad substrate scope, rapid reaction rate and high atomic economy, providing a facile avenue for the synthesis of aryl sulfides.

## Data availability

The datasets generated and/or analyzed during the current study are available from the corresponding author on reasonable request. The data includes experimental procedures and compound characterizations using NMR and HRMS.

## Conflicts of interest

There are no conflicts to declare.

## Supplementary Material

RA-015-D5RA00100E-s001
