# Supplementary material for: Iodine-mediated thio-arylation under electrochemical conditions
Source: RSC Adv. 2025 Apr 16;15(15):12042–6. doi: 10.1039/d5ra00100e (PMC12001020; doi:10.1039/d5ra00100e)

Supplementary Information (SI) for RSC Advances.
This journal is © The Royal Society of Chemistry 2025

Supporting Information

Iodine-mediated Thio-arylation under Electrochemistry

Jiajia Yu[1], Tong Li[2], Qi Sun[*1] and Zhiyong Wang[*1, 2]

[1]Institute of Advanced Technology, University of Science and Technology of China. Hefei 230000, China. [*]Hefei National Center for Physical Sciences at Microscale, Key Laboratory of Precision and Intelligent Chemistry, School of Chemistry and Materials Science, University of Science and Technology of China, Hefei 230026, China.

[*]E-mail: zwang3@ustc.edu.cn.

Table of Contents

1. **General Information S2**
2. **Experimental Procedure S2**
3. **Optimization of Reaction Conditions S3**
4. **Cyclic Voltammetry Studies S3**
5. **Control experiments S5**
6. **Detail Descriptions for Products S5**
7. **Supporting Refrence...S14**
8. **NMR Spectra for the Products.......................................S15**

1. General Information

Unless otherwise noted, materials were obtained from commercial suppliers and used without further purification. NMR spectra were recorded on a Bruker AV-500 (1H: 500 MHz, ^{13}C: 125 MHz, ^{19}F NMR: 470 MHz) spectrometer using TMS as internal reference. Chemical shifts (δ) and coupling constants (*J*) were expressed in ppm and Hz, respectively. GC-MS was Shimadzu QP-5050 GC-MS system. Commercially available compounds were used without further purification. All substances were known compounds and synthesized according to the references. High resolution mass spectra (HRMS) were measured using electrospray ionization (ESI) and the time-of-flight (TOF) mass analyzer. The anode electrode and cathode electrode all are Pt (1.0 × 1.0 cm^2). These electrodes are commercially available from GaossUnion, China.

2. Experimental Procedure

Typical Procedure for 4-aminophenyl phenyl sulfide: A mixture of diphenyl disulfide (0.15 mmol), aniline (0.9 mmol), Et_4NI (0.3 mmol), DMSO = 3 mL and H_2O = 0.3 mL was added to an undivided cell. The cell was equipped with platinum electrode as both the anode and cathode. The reaction mixture was stirred and electrolyzed at a constant current of 10 mA under 120 °C for corresponding time. When the reaction was finished, the solution was extracted with EtOAc (3×10 ml). The solvent was removed with a rotary evaporator. The residue was purified by column chromatography on silica gel (PE/EtOAc = 4:1) to afford the desired product.

Gram-scale synthesis of 3aa: A mixture of diphenyl disulfide (3 mmol) benzylamine (15 mmol), ammonium tetrafluoroborate (5 mmol) and aniline (18 mmol), Et_4NI (6 mmol), DMSO = 60 mL and H_2O = 6 mL was added to an undivided cell. The cell was equipped with platinum electrode as both the anode and cathode. The reaction mixture was stirred and electrolyzed (J = 10 mA/cm^2, I = 23 mA) under 120 °C for 2 days. When the reaction was finished, the solution was extracted with EtOAc (3×100 mL). The combined organic layer was dried with Na_2SO_4, filtered. The solvent was removed with a rotary evaporator. The residue was purified by column chromatography on silica gel (PE/EtOAc = 4:1) to afford the desired product.

3. Optimization of Reaction Conditions

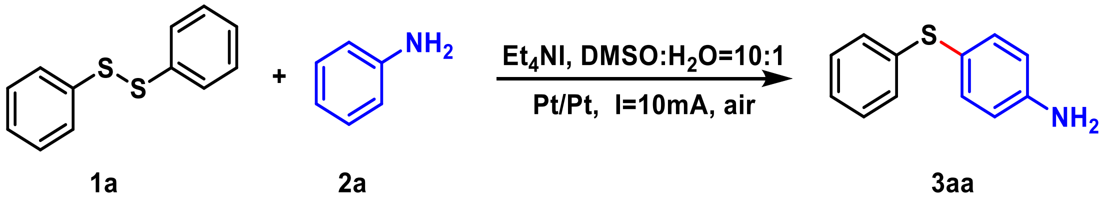

Table S1. the optimization of temperature of reaction.[a]

Entry	Temperature(°C)	Yield[%][b]
1	25	trace
2	40	trace
3	50	trace
4	60	trace
5	80	trace
6	100	40
7	120	86
8	140	80

[a] Standard condition: platinum plate (10 mm × 10 mm × 0.2mm) as the anode, platinum plate (10 mm × 10 mm × 0.2mm) as the cathode, undivided cell, 1a (0.15 mmol), 2a (0.9 mmol), , Et_4NI (0.3 mmol), and DMSO (3 mL), Air, 120℃, 4h. [b] Isolated yield.

4. Cyclic Voltammetry Studies

Cyclic voltammetry data were measured with a Shanghai Chenhua potentiostat (CHI760E).

Working electrode: The working electrode is a 3 mm diameter Pt disk working electrode. Polished with 0.3 μm aluminum oxide and then sonicated in distilled water before drying.

Reference electrode: The reference electrode consisted of a silver wire covered with silver chloride immersed in a saturated solution of potassium chloride.

Counter electrode: The counter electrode is a platinum wire that was polished with sand paper.

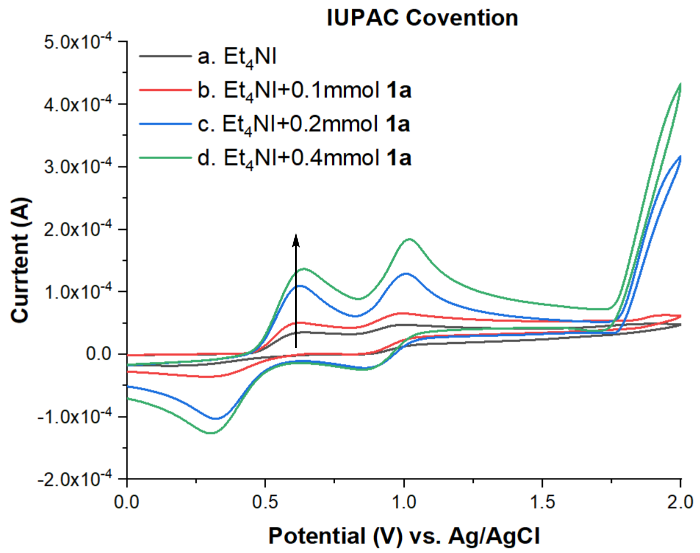

Figure S1. Cyclic voltammetry experiments: Cyclic voltammograms of **1a**, and 0.1 mmol Et4NI in 0.1 M *n*-Bu4NBF4/MeCN = 10 mL using a Pt disk as the working electrode, and Pt wire and Ag/AgCl as the counter and reference electrodes, respectively, at a scan rate of 100 mV·s^{-1}; background (curve a), **1a** (10 mmol·L^{-1}, curve b), **1a** (20 mmol·L^{-1}, curve c) and **1a** (40 mmol·L^{-1}, curve d).

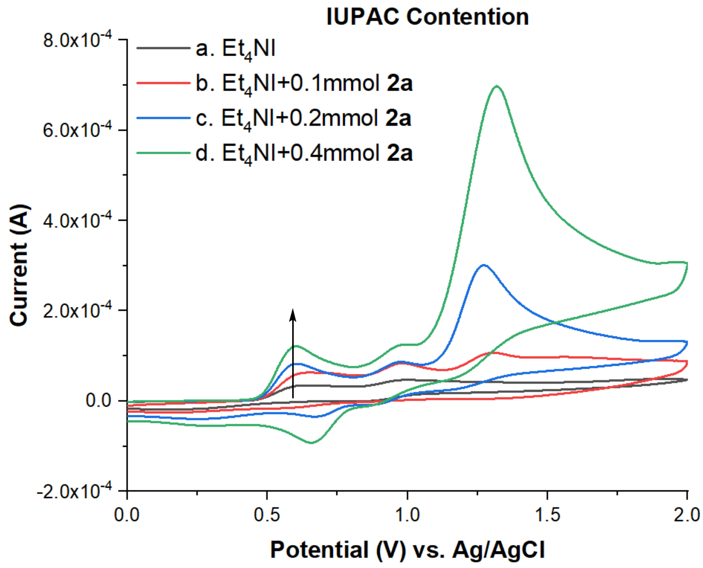

Figure S2. Cyclic voltammetry experiments: Cyclic voltammograms of **2a**, and 0.1 mmol Et4NI in 0.1 M *n*-Bu4NBF4/MeCN = 10 mL using a Pt disk as the working electrode, and Pt wire and Ag/AgCl as the counter and reference electrodes, respectively, at a scan rate of 100 mV·s^{-1}; background (curve a), **2a** (10 mmol·L^{-1}, curve b), **2a** (20 mmol·L^{-1}, curve c) and **2a** (40 mmol·L^{-1}, curve d).

Figure S1 and Figure **S2** investigated the catalytic activity of Et4NI. It can be observed that excessive treatment of Et4NI with **1a** and **2a** led to a significant increase in the anodic peak of the mediator Et4NI, which confirmed the catalytic role of Et4NI in the reaction.

5. Control experiments

We conducted experiments without the addition of oxidants on all substrates. It was ultimately found that when thiols were used as substrates for the reaction, the corresponding products could not be obtained under the condition that iodine was not added as an oxidant. And under I_2/DTBP conditions, the yield after 6 hours of reaction is very low.

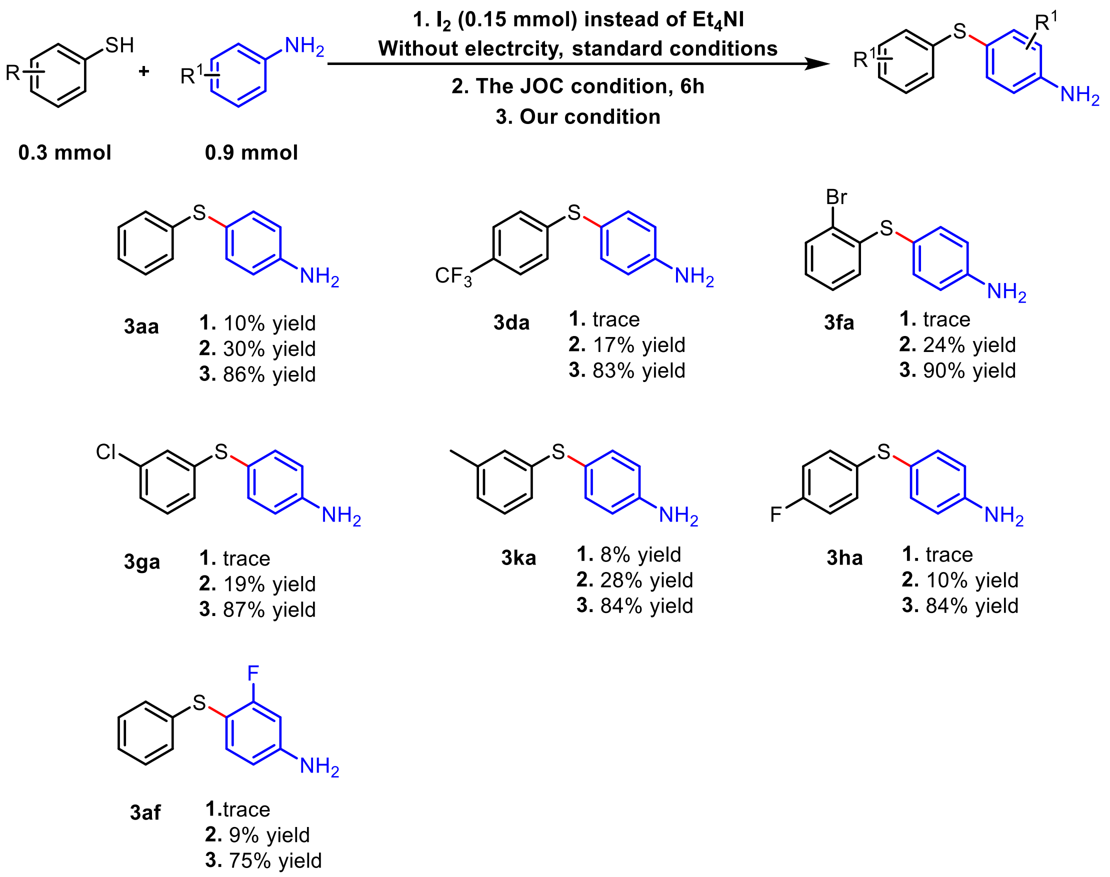

6. Detail Descriptions for Products

4-(phenylthio)aniline (**3aa**)

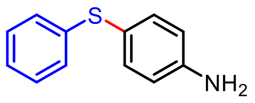

The title compound was prepared according to the general working procedure and purified by column chromatography (petroleum ether/ethyl acetate = 4:1) to give the product as a yellow solid. 86% yield,51.9 mg. M.P. = 76-78 °C. HRMS (ESI) m/z: calcd for $C_{12}H_{12}NS$ $[M+H]^+$ 202.0685, found: 202.0690.

^1H NMR (500 MHz, $CDCl_3$) δ 7.36 – 7.32 (m, 2H), 7.26 – 7.21 (m, 2H), 7.21 – 7.03 (m, 3H), 6.71 – 6.65 (m, 2H), 3.72 (s, 2H).

^{13}C NMR (125 MHz, $CDCl_3$) δ 147.1, 139.7, 136.2, 128.9, 127.3, 125.3, 120.43, 115.9.

4-((4-bromophenyl)thio)aniline (**3ba**)[S1]

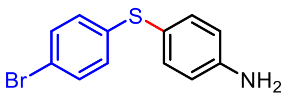

The title compound was prepared according to the general working procedure and purified by column chromatography (petroleum ether/ethyl acetate = 4:1) to give the product as a yellow oil. 86% yield, 72.2 mg.

^{1}H NMR (500 MHz, CDCl$_3$) δ 7.30 (t, *J* = 8.0 Hz, 4H), 6.97 (m,2H), 6.70 – 6.65 (m, 2H), 3.81 (s, 2H).

^{13}C NMR (125 MHz, CDCl$_3$) δ 147.4, 139.3, 136.3, 131.8, 128.6, 119.6, 118.8, 116.0.

4-((4-chlorophenyl)thio)aniline (**3ca**)[S2]

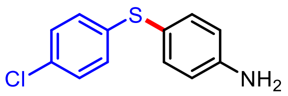

The title compound was prepared according to the general working procedure and purified by column chromatography (petroleum ether/ethyl acetate = 4:1) to give the product as a yellow solid. 81% yield, 57.1 mg. M.P. = 73-75 °C.

^{1}H NMR (500 MHz, CDCl$_3$) δ 7.33 – 7.28 (m, 2H), 7.21 – 7.16 (m, 2H), 7.08 – 7.03 (m, 2H), 6.70 – 6.65 (m, 2H), 3.79 (s, 2H).

^{13}C NMR (125 MHz, CDCl$_3$) δ 147.3, 138.5, 136.2, 131.1, 128.9, 128.5, 119.9, 116.0.

4-((4-(trifluoromethyl)phenyl)thio)aniline (**3da**)

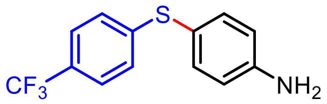

The title compound was prepared according to the general working procedure and purified by column chromatography (petroleum ether/ethyl acetate = 4:1) to give the product as a yellow solid. 82% yield, 66.2 mg. M.P. = 85-87 °C. HRMS (ESI) m/z: calcd for $C_{13}H_{10}F_3NS$ $[M+H]^+$ 270.0559, found 270.0561.

^{1}H NMR (500 MHz, CDCl$_3$) δ 7.42 (d, *J* = 8.3 Hz, 2H), 7.33 (t, *J* = 5.6 Hz, 2H), 7.12 (d, *J* = 8.2 Hz, 2H), 6.73 (d, *J* = 8.5 Hz, 2H), 3.74 (s, 2H).

^{13}C NMR (125 MHz, CDCl$_3$) δ 148.0, 145.9, 137.1, 126.8 (q, *J* = 32.5 Hz), 125.9, 125.6 (q, *J* = 3.7 Hz), 124.4 (q, *J* = 271.5 Hz), 117.8, 116.1.

^{19}F NMR (470 MHz, CDCl$_3$) δ -62.25.

4-((2-chlorophenyl)thio)aniline (**3ea**)[S3]

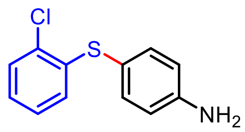

The title compound was prepared according to the general working procedure and purified by column chromatography (petroleum ether/ethyl acetate = 4:1) to give the product as a yellow oil. 81% yield, 57.1mg.

^{1}H NMR (500 MHz, CDCl$_3$) δ 7.37 – 7.29 (m, 3H), 7.03 (m, 2H), 6.75 – 6.69 (m, 3H), 3.74 (s, 2H).

^{13}C NMR (125 MHz, CDCl$_3$) δ 147.8, 139.7, 137.4, 130.4, 129.3, 127.0, 126.9, 125.7, 117.8, 116.2.

4-((2-bromophenyl)thio)aniline (**3fa**)[S4]

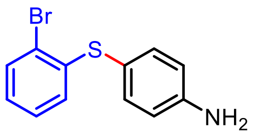

The title compound was prepared according to the general working procedure and purified by column chromatography (petroleum ether/ethyl acetate = 4:1) to give the product as a yellow oil. 90% yield, 75.6 mg.

^{1}H NMR (500 MHz, $CDCl_3$) δ 7.49 (dd, J = 7.9, 1.2 Hz, 1H), 7.36 – 7.31 (m, 2H), 7.10 – 7.06 (m, 1H), 6.93 (td, J = 7.6, 1.5 Hz, 1H), 6.73 – 6.70 (m, 2H), 6.68 (dd, J = 8.0, 1.5 Hz, 1H), 3.75 (s, 2H).

^{13}C NMR (125 MHz, $CDCl_3$) δ 147.8, 141.7, 137.4, 132.6, 127.6, 126.9, 125.9, 120.1, 118.3, 116.2.

4-((3-chlorophenyl)thio)aniline (**3ga**)[S3]

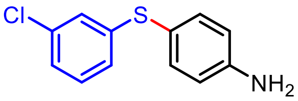

The title compound was prepared according to the general working procedure and purified by column chromatography (petroleum ether/ethyl acetate = 4:1) to give the product as a yellow oil. 87% yield, 61.3 mg.

^{1}H NMR (500 MHz, $CDCl_3$) δ 7.41 – 7.27 (m, 2H), 7.16 – 7.09 (m, 1H), 7.09 – 7.00 (m, 2H), 7.02 – 6.93 (m, 1H), 6.85 – 6.62 (m, 2H), 3.74 (s, 2H).

^{13}C NMR (125 MHz, $CDCl_3$) δ 147.5, 142.4, 136.7, 134.8, 129.8, 126.3, 125.2, 124.8, 118.9, 116.1.

4-((4-fluorophenyl)thio)aniline (**3ha**)[S5]

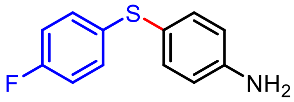

The title compound was prepared according to the general working procedure and purified by column chromatography (petroleum ether/ethyl acetate = 4:1) to give the product as a yellow oil. 64% yield, 42.0 mg.

^{1}H NMR (500 MHz, $CDCl_3$) δ 7.29 – 7.23 (m, 2H), 7.16 – 7.12 (m, 2H), 6.95 – 6.90 (m, 2H), 6.70 – 6.64 (m, 2H), 3.38 (s, 2H).

^{13}C NMR (125 MHz, $CDCl_3$) δ 161.3 (d, J = 245.2 Hz), 146.6, 135.4, 134.2 (d, J = 3.2 Hz), 130.0 (d, J = 8.0 Hz), 121.6, 116.0, 115.9 (d, J = 22.5 Hz).

^{19}F NMR (470 MHz, $CDCl_3$) δ -117.06.

4-(p-tolylthio)aniline (**3ia**)[S6]

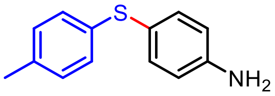

The title compound was prepared according to the general working procedure and purified by column chromatography (petroleum ether/ethyl acetate = 4:1) to give the product as a yellow oil. 83% yield, 53.5 mg.

^{1}H NMR (500 MHz, $CDCl_3$) δ 7.33 – 7.28 (m, 2H), 7.14 – 7.04 (m, 4H), 6.69 – 6.64 (m, 2H), 3.73 (s, 2H), 2.31 (s, 3H).

^{13}C NMR (125 MHz, $CDCl_3$) δ 146.7, 135.6, 135.5, 135.4, 129.7, 128.3, 121.7, 115.9, 21.0.

4-(o-tolylthio)aniline (**3ja**)[S3]

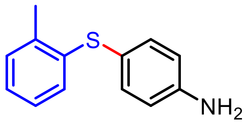

The title compound was prepared according to the general working procedure and purified by column chromatography (petroleum ether/ethyl acetate = 4:1) to give the product as a yellow oil. 88% yield, 56.7 mg.

^{1}H NMR (500 MHz, $CDCl_3$) δ 7.31 – 7.27 (m, 2H), 7.20 – 7.16 (m, 1H), 7.10 – 7.04 (m, 2H), 6.93 (m, 1H), 6.71 – 6.67 (m, 2H), 3.68 (s, 2H), 2.42 (s, 3H).

^{13}C NMR (125 MHz, $CDCl_3$) δ 146.8, 138.5, 136.0, 135.8, 130.1, 127.6, 126.4, 125.4, 120.4, 116.1, 20.3.

4-(m-tolylthio)aniline (**3ka**)[S3]

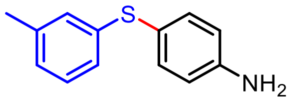

The title compound was prepared according to the general working procedure and purified by column chromatography (petroleum ether/ethyl acetate = 4:1) to give the product as a yellow oil. 84% yield, 54.1 mg.

^{1}H NMR (500 MHz, $CDCl_3$) δ 7.35 – 7.31 (m, 2H), 7.12 (t, *J* = 7.7 Hz, 1H), 7.00 (s, 1H), 6.97 – 6.91 (m, 2H), 6.71 – 6.66 (m, 2H), 3.65 (s, 2H), 2.28 (s, 3H).

^{13}C NMR (125 MHz, $CDCl_3$) δ 146.9, 139.3, 138.7, 136.0, 128.8, 128.0, 126.3, 124.5, 120.8, 116.0, 21.4.

4-((4-methoxyphenyl)thio)aniline (**3la**)[S7]

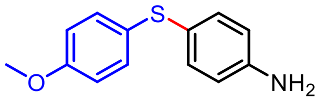

The title compound was prepared according to the general working procedure and purified by column chromatography (petroleum ether/ethyl acetate = 4:1) to give the product as a yellow oil. 68% yield, 47.1 mg.

^{1}H NMR (500 MHz, $CDCl_3$) δ 7.26 – 7.18 (m, 4H), 6.82 (d, *J* = 8.7 Hz, 2H), 6.62 (d, *J* = 8.4 Hz, 2H), 3.77 (s, 3H), 3.64 (s, 2H).

^{13}C NMR (125 MHz, $CDCl_3$) δ 158.6, 146.2, 134.0, 131.5, 128.9, 123.5, 115.9, 114.7, 55.4.

4-((3-methoxyphenyl)thio)aniline (**3ma**)[S8]

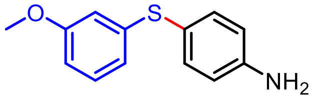

The title compound was prepared according to the general working procedure and purified by column chromatography (petroleum ether/ethyl acetate = 4:1) to give the product as a yellow oil. 76% yield, 52.6 mg.

^{1}H NMR (500 MHz, $CDCl_3$) δ 7.36 – 7.32 (m, 2H), 7.15 (t, *J* = 8.0 Hz, 1H), 6.75 – 6.65 (m, 5H), 3.80 – 3.68 (m, 5H).

^{13}C NMR (125 MHz, $CDCl_3$) δ 160.0, 147.2, 141.3, 136.4, 129.7, 119.9, 119.4, 116.0, 112.6, 110.9, 55.2.

4-((2-methylfuran-3-yl)thio)aniline (**3na**)

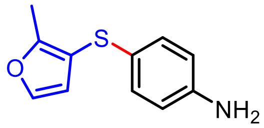

The title compound was prepared according to the general working procedure and purified by column chromatography (petroleum ether/ethyl acetate = 4:1) to give the product as a yellow oil. 55% yield, 33.8 mg. HRMS (ESI) m/z: calcd for

$C_{11}H_{11}NOS$ $[M+H]^+$ 206.0634, found 206.0634.
^1H NMR (500 MHz, $CDCl_3$) δ 7.29 (d, J = 1.9 Hz, 1H), 7.08 – 7.04 (m, 2H), 6.61 – 6.58 (m, 2H), 6.32 (d, J = 1.9 Hz, 1H), 3.45 (s, 2H), 2.36 (s, 3H).
^{13}C NMR (125 MHz, $CDCl_3$) δ 155.1, 144.9, 140.8, 130.3, 125.3, 115.9, 115.0, 110.6, 11.9.

2-methyl-4-(phenylthio)aniline (**3ab**)[S9]

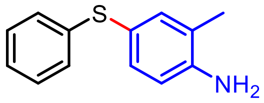

The title compound was prepared according to the general working procedure and purified by column chromatography (petroleum ether/ethyl acetate = 4:1) to give the product as a yellow solid. 88% yield, 56.8 mg.
^1H NMR (500 MHz, $CDCl_3$) δ 7.24 – 7.16 (m, 4H), 7.13 – 7.05 (m, 3H), 6.64 (d, J = 8.1 Hz, 1H), 3.69 (s, 2H), 2.12 (s, 3H).
^{13}C NMR (125 MHz, $CDCl_3$) δ 145.4, 140.0, 137.1, 134.0, 128.9, 127.1, 125.2, 123.4, 120.0, 115.7, 17.3.

3-methyl-4-(phenylthio)aniline (**3ac**)

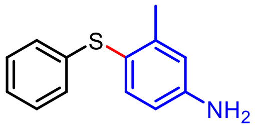

The title compound was prepared according to the general working procedure and purified by column chromatography (petroleum ether/ethyl acetate = 4:1) to give the product as a yellow oil: 81% yield, 52.2 mg. HRMS (ESI) m/z: calcd for $C_{13}H_{13}NS$ $[M+H]^+$ 216.0841, found 216.0843.
^1H NMR (500 MHz, $CDCl_3$) δ 7.36 (d, J = 8.2 Hz, 1H), 7.22 (m, 2H), 7.12 – 7.08 (m, 1H), 7.06 (m, 2H), 6.65 (d, J = 2.4 Hz, 1H), 6.55 (dd, J = 8.2, 2.5 Hz, 1H), 3.72 (s, 2H), 2.32 (s, 3H).
^{13}C NMR (125 MHz, $CDCl_3$) δ 147.7, 144.0, 139.5, 138.0, 128.9, 126.3, 124.8, 119.2, 117.3, 113.6, 20.9.

3-bromo-4-(phenylthio)aniline (**3ad**)

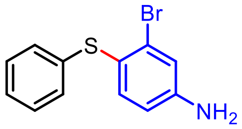

The title compound was prepared according to the general working procedure and purified by column chromatography (petroleum ether/ethyl acetate = 4:1) to give the product as a yellow oil. 85% yield, 71.4 mg. HRMS (ESI) m/z: calcd for $C_{12}H_{10}ClNS$ $[M+H]^+$ 279.9790, found 279.9789.
^1H NMR (500 MHz, $CDCl_3$) δ 7.24 (m, 3H), 7.17 – 7.10 (m, 3H), 6.96 (d, J = 2.5 Hz, 1H), 6.53 (dd, J = 8.4, 2.5 Hz, 1H), 3.70 (s, 2H).
^{13}C NMR (125 MHz, $CDCl_3$) δ 148.2, 137.6, 137.1, 130.2, 129.1, 128.0, 125.9, 121.6, 119.4, 115.0.

3-chloro-4-(phenylthio)aniline (**3ae**)

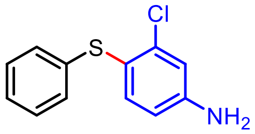

The title compound was prepared according to the general working procedure and purified by column chromatography (petroleum ether/ethyl acetate = 4:1) to give the product as a yellow oil. 80% yield, 56.4 mg. HRMS (ESI) m/z: calcd for

$C_{12}H_{10}ClNS$ [M+H]$^+$ 236.0295, found 236.0298.
^{1}H NMR (500 MHz, $CDCl_3$) δ 7.30 – 7.19 (m, 3H), 7.15 – 7.10 (m, 3H), 6.77 (d, *J* = 2.5 Hz, 1H), 6.50 (dd, *J* = 8.4, 2.5 Hz, 1H), 3.73 (s, 2H).
^{13}C NMR (125 MHz, $CDCl_3$) δ 148.3, 139.4, 137.5, 137.4, 129.0, 127.8, 125.8, 119.4, 116.2, 114.3.

3-fluoro-4-(phenylthio)aniline (**3af**)

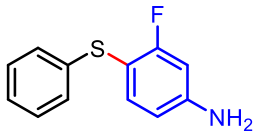

The title compound was prepared according to the general working procedure and purified by column chromatography (petroleum ether/ethyl acetate = 4:1) to give the product as a yellow solid. 75% yield, 49.3 mg. M.P. = 73-75 °C HRMS (ESI) m/z: calcd for $C_{12}H_{10}FNS$ [M+H]$^+$ 220.0591, found 220.0591.
^{1}H NMR (500 MHz, $CDCl_3$) δ 7.30 – 7.25 (m, 1H), 7.24 – 7.18 (m, 2H), 7.17 – 7.06 (m, 3H), 6.48 – 6.40 (m, 2H), 3.85 (s, 2H).
^{13}C NMR (125 MHz, $CDCl_3$) δ 163.9 (d, *J* = 246.0 Hz), 149.8, 149.7, 138.2, 138.2, 138.1, 128.9, 127.1, 125.5, 111.5 (d, *J* = 2.8 Hz), 106.7 (d, *J* = 19.0 Hz), 102.4 (d, *J* = 26.6 Hz).
^{19}F NMR (470 MHz, $CDCl_3$) δ -106.28.

3-methoxy-4-(phenylthio)aniline (**3ag**)

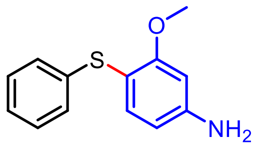

The title compound was prepared according to the general working procedure and purified by column chromatography (petroleum ether/ethyl acetate = 4:1) to give the product as a yellow solid. 70% yield, 48.5 mg. HRMS (ESI) m/z: calcd for $C_{13}H_{13}NOS$ [M+H]$^+$ 232.0791, found 232.0792.
^{1}H NMR (500 MHz, $CDCl_3$) δ 7.28 – 7.25 (m, 1H), 7.23 – 7.18 (m, 2H), 7.14 – 7.07 (m, 3H), 6.34 – 6.28 (m, 2H), 3.78 (s, 3H), 3.52 (s, 2H).
^{13}C NMR (125 MHz, $CDCl_3$) δ 160.9, 149.2, 138.9, 138.0, 128.7, 126.8, 124.9, 108.1, 107.9, 98.8, 55.9.

2-bromo-4-(phenylthio)aniline (**3ah**)

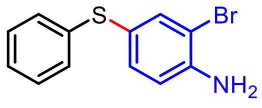

The title compound was prepared according to the general working procedure and purified by column chromatography (petroleum ether/ethyl acetate = 4:1) to give the product as a yellow oil. 55% yield, 46.2 mg. HRMS (ESI) m/z: calcd for $C_{12}H_{10}BrNS$ [M+H]$^+$ 279.9790, found 279.9792.
^{1}H NMR (500 MHz, $CDCl_3$) δ 7.58 (d, *J* = 2.0 Hz, 1H), 7.23 (m, 3H), 7.16 – 7.11 (m, 3H), 6.73 (d, *J* = 8.3 Hz, 1H), 4.13 (s, 2H).
^{13}C NMR (125 MHz, $CDCl_3$) δ 144,5, 138.7, 138.2, 134.7, 129.0, 127.9, 125.8, 122.1, 116.2, 109.3.

2-chloro-4-(phenylthio)aniline (**3ai**)[S10]

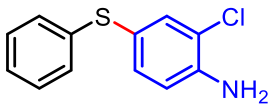

The title compound was prepared according to the general working procedure and purified by column chromatography (petroleum ether/ethyl acetate = 4:1) to give the product as a yellow oil. 50% yield, 35.3 mg.

^{1}H NMR (500 MHz, $CDCl_3$) δ 7.41 (d, J = 2.0 Hz, 1H), 7.25 – 7.19 (m, 3H), 7.17 – 7.11 (m, 3H), 6.73 (d, J = 8.3 Hz, 1H), 3.96 (s, 2H).

^{13}C NMR (125 MHz, $CDCl_3$) δ 143.3, 138.6, 135.1, 134.0, 129.0, 128.0, 125.8, 121.8, 119.5, 116.3.

2-(tert-butyl)-4-(phenylthio)aniline (**3aj**)

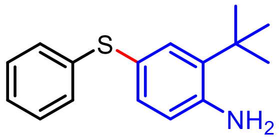

The title compound was prepared according to the general working procedure and purified by column chromatography (petroleum ether/ethyl acetate = 4:1) to give the product as a yellow oil. 70% yield, 54.0 mg. HRMS (ESI) m/z: calcd for $C_{16}H_{19}NS$ $[M+H]^+$ 258.1311, found 258.1313.

^{1}H NMR (500 MHz, $CDCl_3$) δ 7.39 (d, J = 2.1 Hz, 1H), 7.22 – 7.15 (m, 3H), 7.13 – 7.06 (m, 3H), 6.61 (d, J = 8.1 Hz, 1H), 3.82 (s, 2H), 1.39 (s, 9H).

^{13}C NMR (126 MHz, $CDCl_3$) δ 145.4, 139.9, 134.6, 133.6, 133.6, 128.9, 127.1, 125.2, 120.1, 118.6, 34.4, 29.5.

2-methoxy-4-(phenylthio)aniline (**3ak**)[S11]

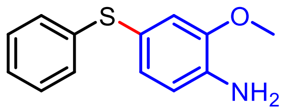

The title compound was prepared according to the general working procedure and purified by column chromatography (petroleum ether/ethyl acetate = 4:1) to give the product as a yellow oil. 66% yield, 45.7 mg.

^{1}H NMR (500 MHz, $CDCl_3$) δ 7.23 (t, J = 7.6 Hz, 2H), 7.16 – 7.09 (m, 3H), 7.01 (dd, J = 8.0, 1.8 Hz, 1H), 6.96 (d, J = 1.7 Hz, 1H), 6.71 (d, J = 8.0 Hz, 1H), 3.82 (s, 3H), 3.80 (s, 2H).

^{13}C NMR (125 MHz, $CDCl_3$) δ 147.6, 139.8, 137.0, 128.9, 128.3, 127.1, 125.2, 120.1, 116.7, 115.2, 55.6.

2,6-dimethyl-4-(phenylthio)aniline (**3al**)

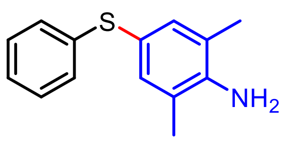

The title compound was prepared according to the general working procedure and purified by column chromatography (petroleum ether/ethyl acetate = 4:1) to give the product as a yellow oil. 83% yield, 57.0 mg. HRMS (ESI) m/z: calcd for $C_{14}H_{15}NS$ $[M+H]^+$ 230.0998, found 230.1002.

^{1}H NMR (500 MHz, $CDCl_3$) δ 7.18 (m, 2H), 7.14 – 7.04 (m, 5H), 3.63 (s, 2H), 2.13 (s, 6H).

^{13}C NMR (125 MHz, $CDCl_3$) δ 143.6, 140.2, 135.0, 128.8, 1271, 125.1, 122.8, 119.2, 17.6.

3,5-dimethyl-4-(phenylthio)aniline (**3am**)

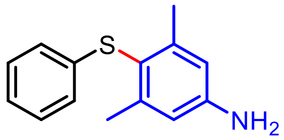

The title compound was prepared according to the general working procedure and purified by column chromatography (petroleum ether/ethyl acetate = 4:1) to give the product as a yellow solid. 73% yield, 50.1mg. M.P. = 103-105 °C HRMS (ESI) m/z: calcd for $C_{13}H_{13}NS$ $[M+H]^+$ 230.0998, found 230.1003.

^{1}H NMR (500 MHz, $CDCl_3$) δ 7.22 – 7.16 (m, 2H), 7.08 – 7.03 (m, 1H), 6.95 (m, 2H), 6.55 (s, 2H), 3.60 (s, 2H), 2.36 (s, 6H).

^{13}C NMR (125 MHz, $CDCl_3$) δ 147.3, 145.4, 139.4, 128.8, 125.0, 124.2, 118.3, 115.0, 21.9.

N-methyl-4-(phenylthio)aniline (**3an**)

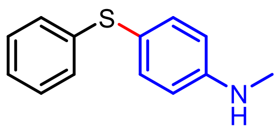

The title compound was prepared according to the general working procedure and purified by column chromatography (petroleum ether/ethyl acetate = 4:1) to give the product as a yellow oil. 73% yield, 47.1 mg. HRMS (ESI) m/z: calcd for $C_{13}H_{13}NS$ $[M+H]^+$ 216.0841, found 216.0846.

^{1}H NMR (500 MHz, $CDCl_3$) δ 7.40 – 7.37 (m, 2H), 7.25 – 7.21 (m, 2H), 7.16 – 7.08 (m, 3H), 3.76 (s, 1H), 2.87 (s, 3H).

^{13}C NMR (125 MHz, $CDCl_3$) δ 149.8, 140.3, 136.4, 128.8, 126.9, 125.1, 118.4, 113.2, 30.6.

N,*N*-dimethyl-4-(phenylthio)aniline (**3ao**)

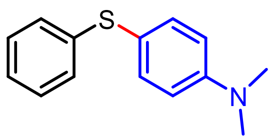

The title compound was prepared according to the general working procedure and purified by column chromatography (petroleum ether/ethyl acetate = 4:1) to give the product as a yellow oil. 77% yield, 52.9 mg. HRMS (ESI) m/z: calcd for $C_{14}H_{15}NS$ $[M+H]^+$ 230.0998, found 230.0994.

^{1}H NMR (500 MHz, $CDCl_3$) δ 7.46 – 7.42 (m, 2H), 7.26 – 7.22 (m, 2H), 7.17 – 7.09

(m, 3H), 6.77 – 6.73 (m, 2H), 3.02 (s, 6H).
^{13}C NMR (125 MHz, $CDCl_3$) δ 150.7, 140.4, 136.2, 128.8, 126.9, 125.0, 113.1, 40.4.

4-(4-(phenylthio)phenyl)morpholine (**3ap**)[S12]

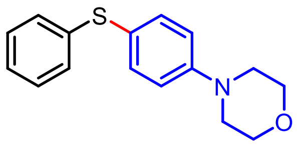

The title compound was prepared according to the general working procedure and purified by column chromatography (petroleum ether/ethyl acetate = 4:1) to give the product as a White solid. 65% yield, 52.8 mg.
^{1}H NMR (500 MHz, $CDCl_3$) δ 7.40 (dd, *J* = 6.8, 4.8 Hz, 2H), 7.23 (t, *J* = 7.6 Hz, 2H), 7.20 – 7.10 (m, 3H), 6.90 (d, *J* = 8.7 Hz, 2H), 3.90 – 3.84 (m, 4H), 3.24 – 3.18 (m, 4H).
^{13}C NMR (125 MHz, $CDCl_3$) δ 151.1, 138.9, 135.2, 128.9, 128.0, 125.6, 122.6, 116.1, 66.8, 48.7.

Methyl 2-amino-5-(phenylthio)benzoate (**3aq**)

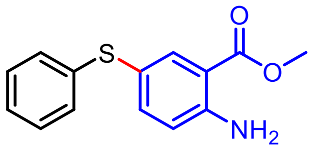

The title compound was prepared according to the general working procedure and purified by column chromatography (petroleum ether/ethyl acetate = 4:1) to give the product as a yellow oil. 60% yield, 46.6 mg. HRMS (ESI) m/z: calcd for $C_{14}H_{13}NO_2S$ $[M+Na]^+$ 282.0559, found 282.0545.
^{1}H NMR (500 MHz, $CDCl_3$) δ 8.10 (d, *J* = 2.1 Hz, 1H), 7.39 (dd, *J* = 8.5, 2.2 Hz, 1H), 7.22 (t, *J* = 7.7 Hz, 2H), 7.14 – 7.09 (m, 3H), 6.68 (d, *J* = 8.6 Hz, 1H), 5.90 (s, 2H), 3.86 (s, 3H).
^{13}C NMR (126 MHz, $CDCl_3$) δ 168.0, 150.7, 140.6, 139.3, 138.3, 128.9, 127.24, 125.4, 118.3, 118.0, 111.5, 51.7.

7. Supporting Refrence

[S1] J. Vicente, J. A. Abad, R. M. López-Nicolás, *Tetrahedron* **2008**, 64, 6281-6288.
[S2] D. Yang, K. Yan, W. Wei, J. Zhao, M. Zhang, X. Sheng, G. Li, S. Lu, H. Wang, *J. Org. Chem*. **2015,** 80, 6083-6092.
[S3] X. Jiang, Z. Shen, C. Zheng, *Tetrahedron Letters.* 61, (**2020**) 152141.
[S4] W. Zhao, F. Zhang, G. Deng, *J. Org. Chem*. **2021**, 86, 291-301.
[S5] X.-L. Fang, R.-Y. Tang, X.-G. Zhang, J.-H. Li, *Synthesis* 2011, **2011**,1099-1105.
[S6] H. Tian, H. Yang, C. Zhu, H. Fu, *Adv. Synth. Catal.* **2015,** 357, 481-488.
[S7] D. Yang, K. Yan, W. Wei, J. Zhao, M. Zhang, X. Sheng, G. Li, S. Lu, H. Wang, *J. Org. Chem.* **2015**, 80, 6083-6092.
[S8] M. Kabir, M. Lorenz, M. Van Linn, *J. Org. Chem*. **2010**, 75, 3626–3643.
[S9] D. S. Raghuvanshi and N. Verma, *RSC Adv.,* **2017**, 7, 22860-22868.
[S10] F. Kwong, S. L. Buchwald, *Org. Lett.*, Vol. 4, No. 20, **2002.**
[S11] Miura S, Shimada M, Marui S, inventors; Patent PCT Int. Appl. WO 2007052843. *Chem. Abstr.* **2007**, 146, 501048.
[S12] Y. Liu, L. Lam, J. Ye, *Adv. Synth. Catal.* **2020,** 362, 2326-2331.

8. NMR Spectra for the Products

4-(phenylthio)aniline (**3aa**)

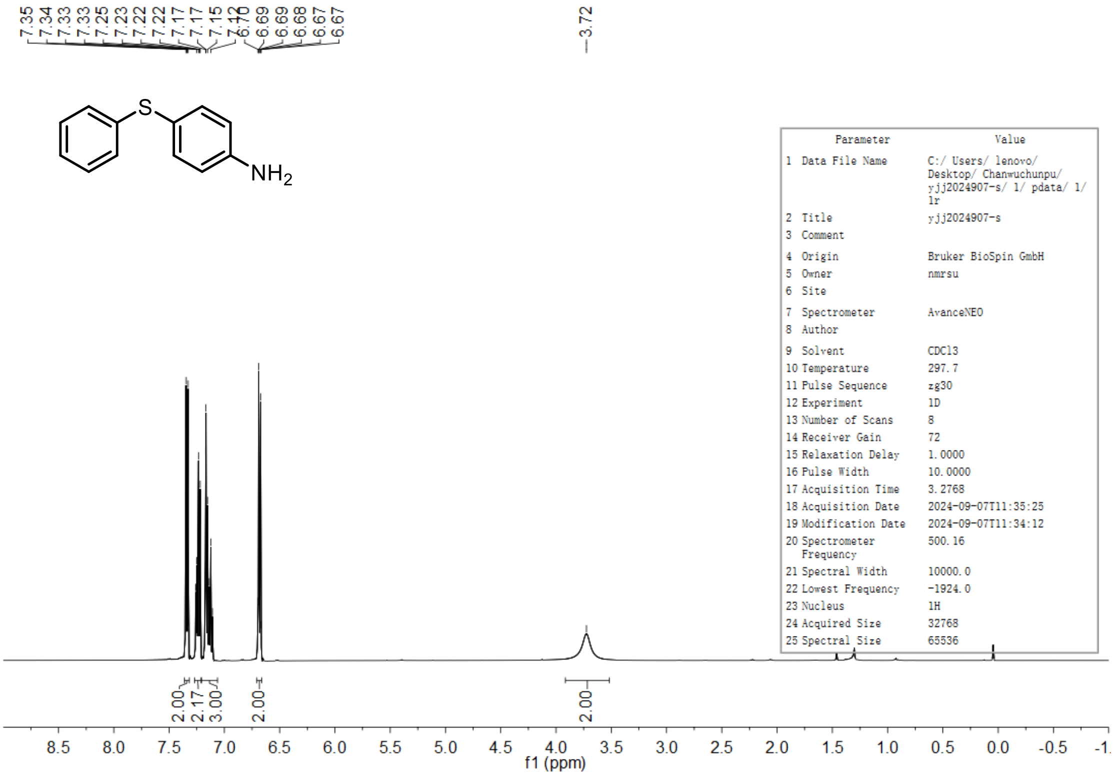

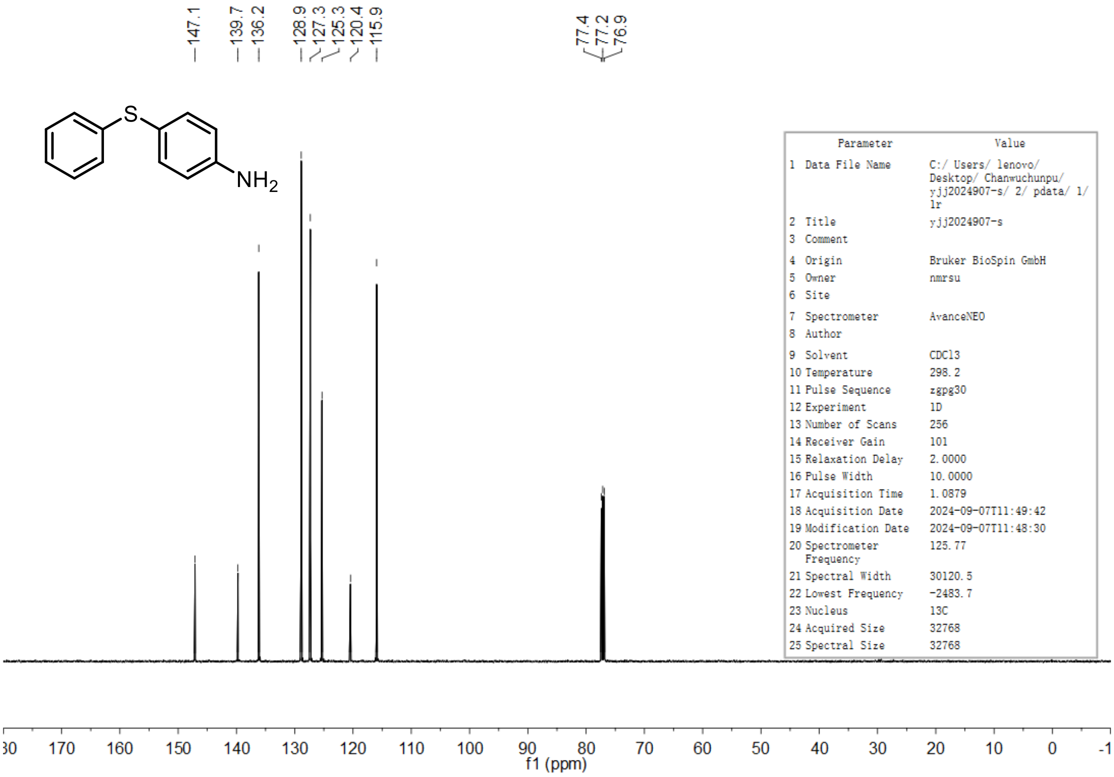

4-((4-bromophenyl)thio)aniline (**3ba**)

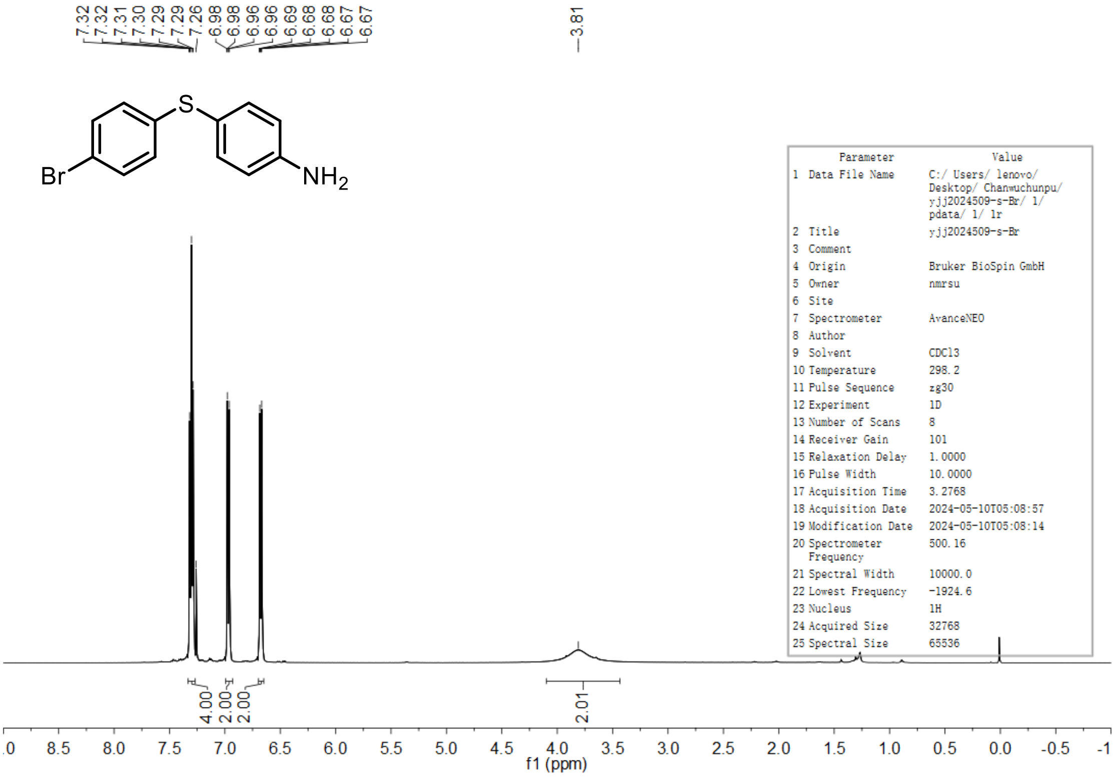

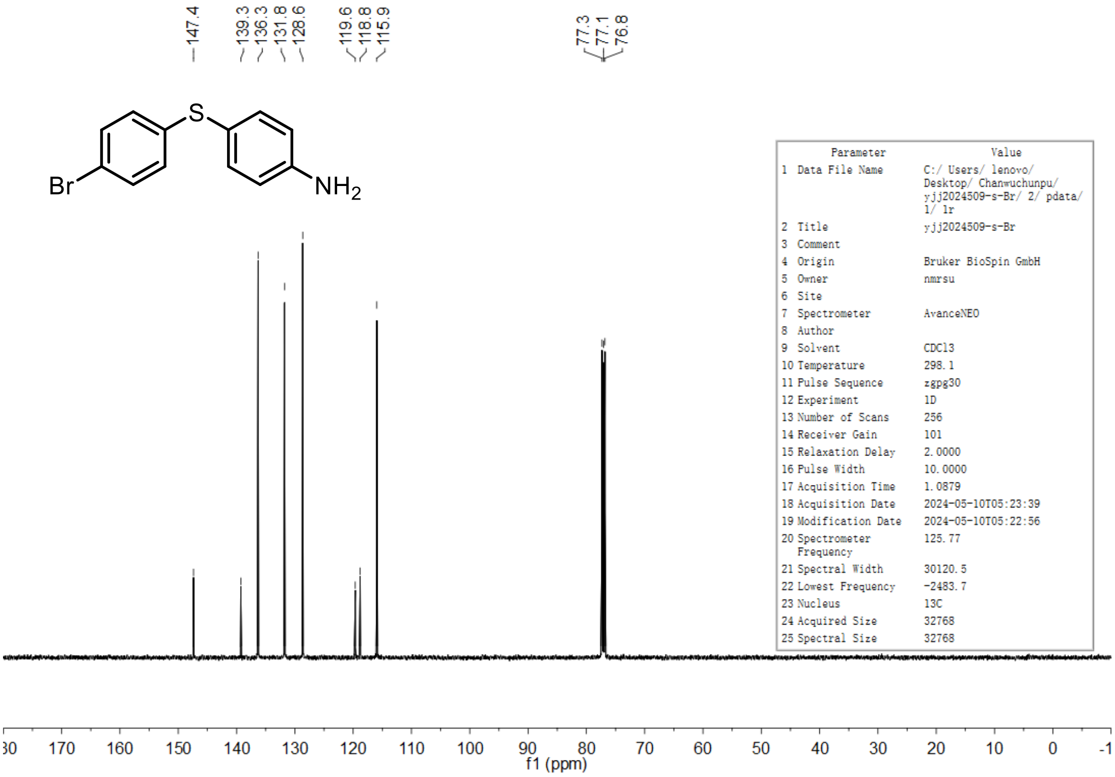

4-((4-chlorophenyl)thio)aniline (**3ca**)

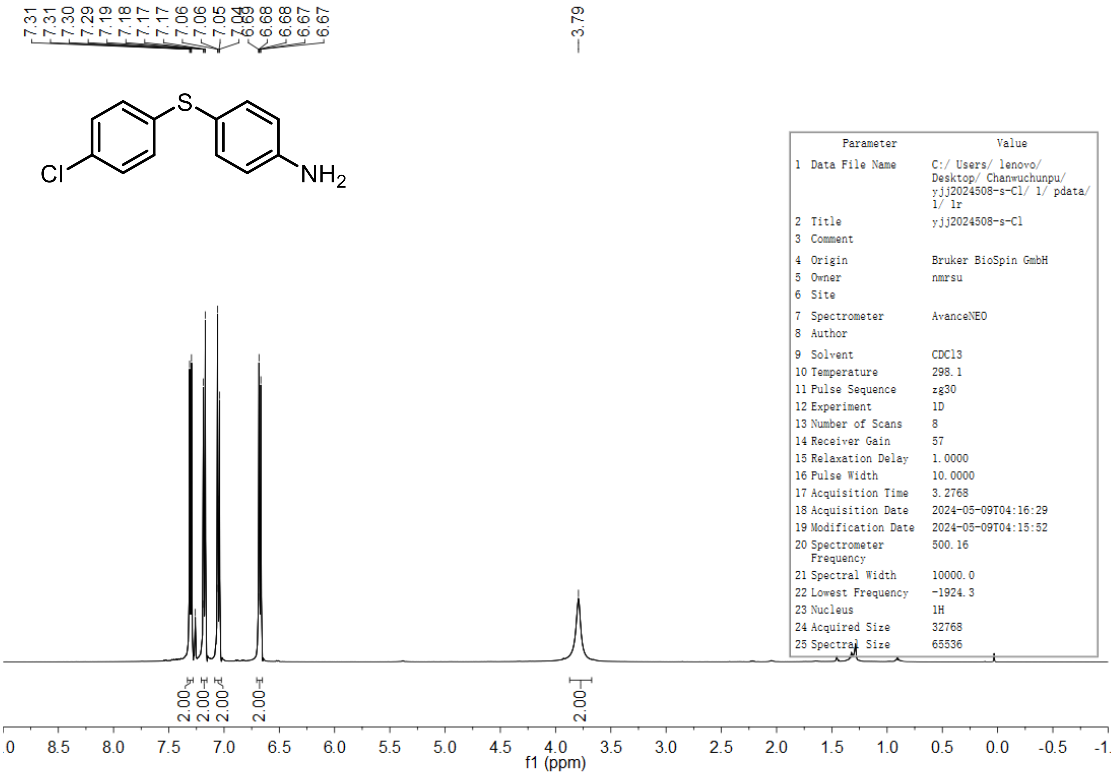

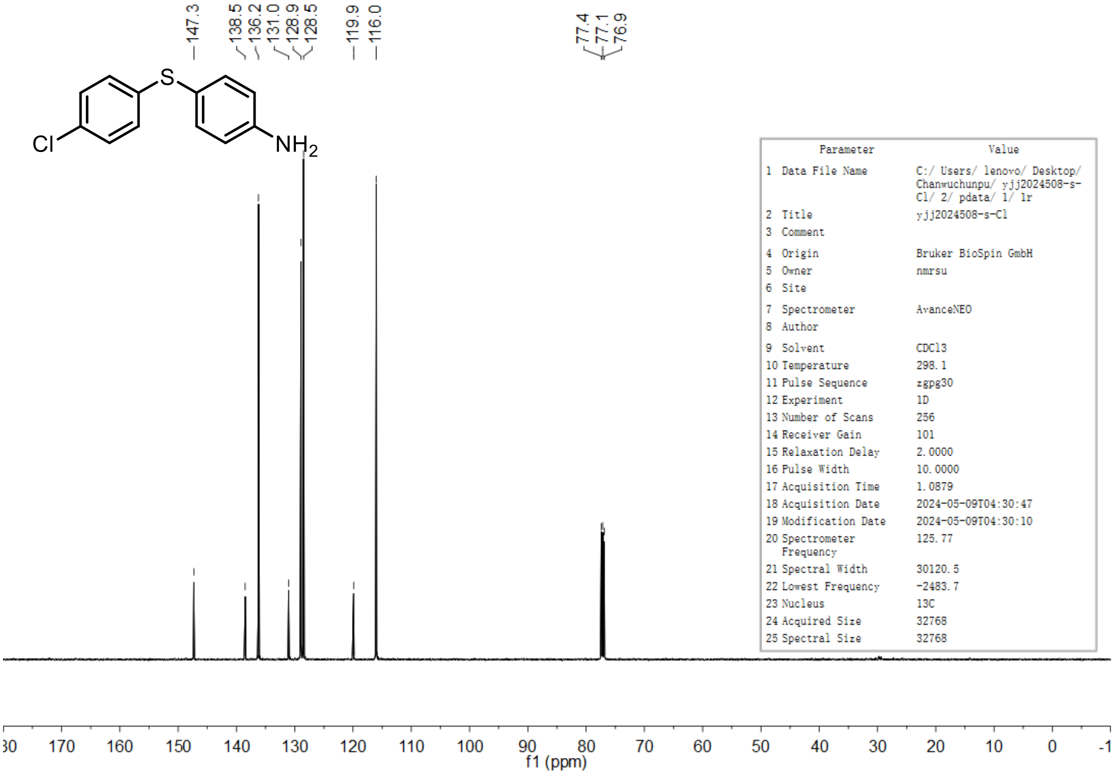

4-((4-(trifluoromethyl)phenyl)thio)aniline (**3da**)

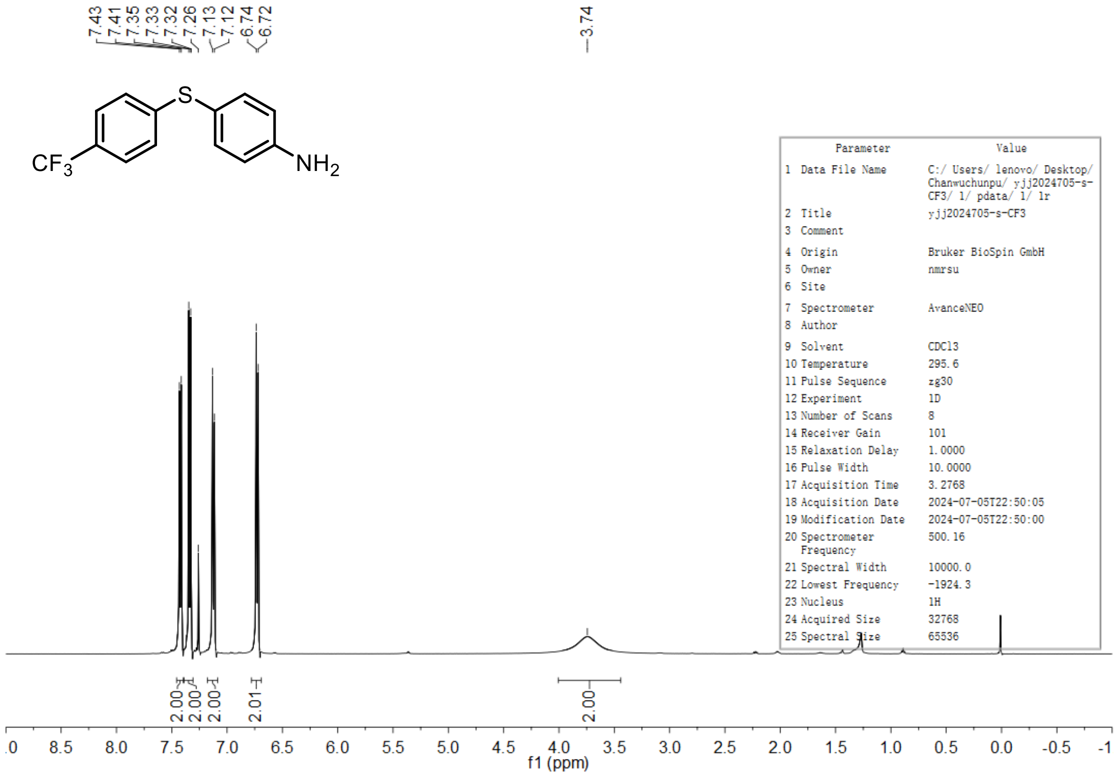

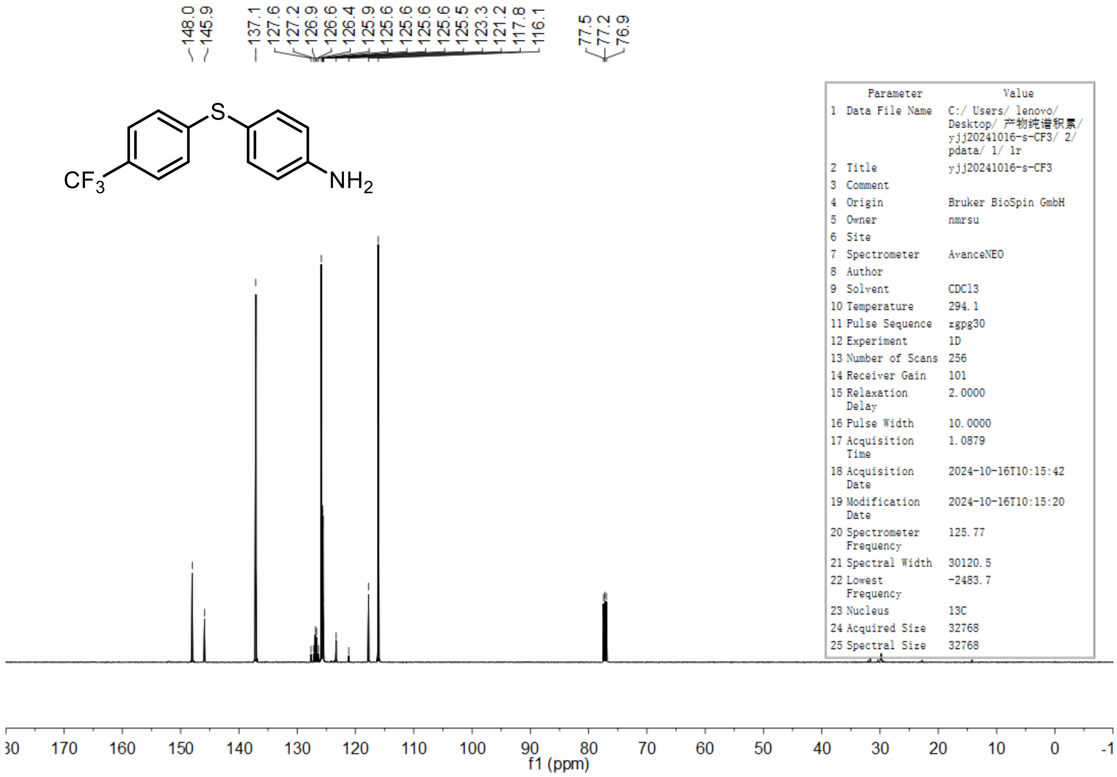

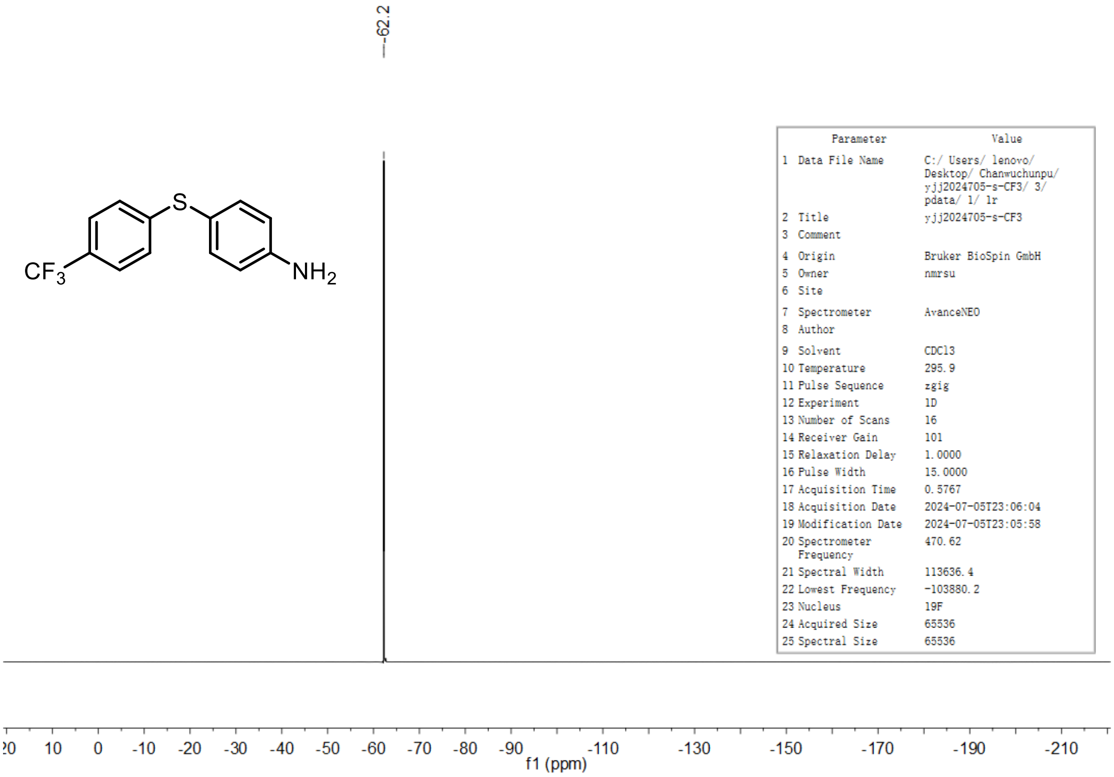

Parameter	Value
1 Data File Name	C:/ Users/ lenovo/ Desktop/ Chanwuchunpu/ yjj2024705-s-CF3/ 3/ pdata/ 1/ 1r
2 Title	yjj2024705-s-CF3
3 Comment	
4 Origin	Bruker BioSpin GmbH
5 Owner	nmrsu
6 Site	
7 Spectrometer	AvanceNEO
8 Author	
9 Solvent	CDCl3
10 Temperature	295.9
11 Pulse Sequence	zgig
12 Experiment	1D
13 Number of Scans	16
14 Receiver Gain	101
15 Relaxation Delay	1.0000
16 Pulse Width	15.0000
17 Acquisition Time	0.5767
18 Acquisition Date	2024-07-05T23:06:04
19 Modification Date	2024-07-05T23:05:58
20 Spectrometer Frequency	470.62
21 Spectral Width	113636.4
22 Lowest Frequency	-103880.2
23 Nucleus	19F
24 Acquired Size	65536
25 Spectral Size	65536

4-((2-chlorophenyl)thio)aniline (**3ea**)

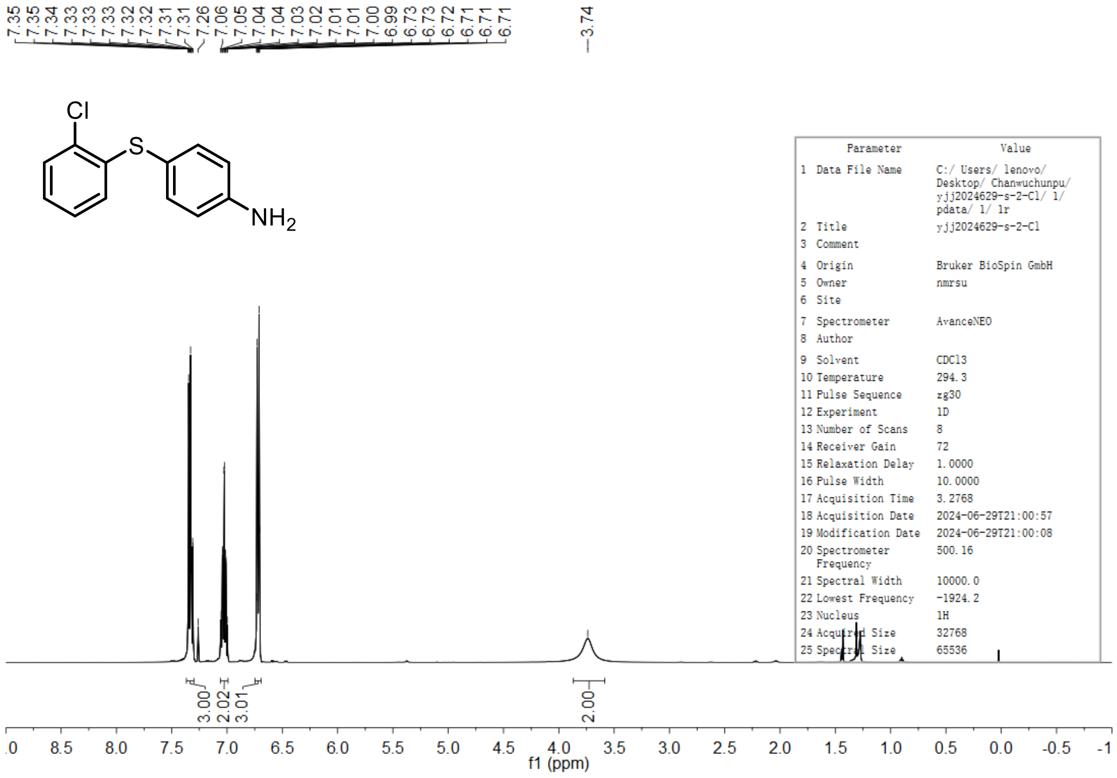

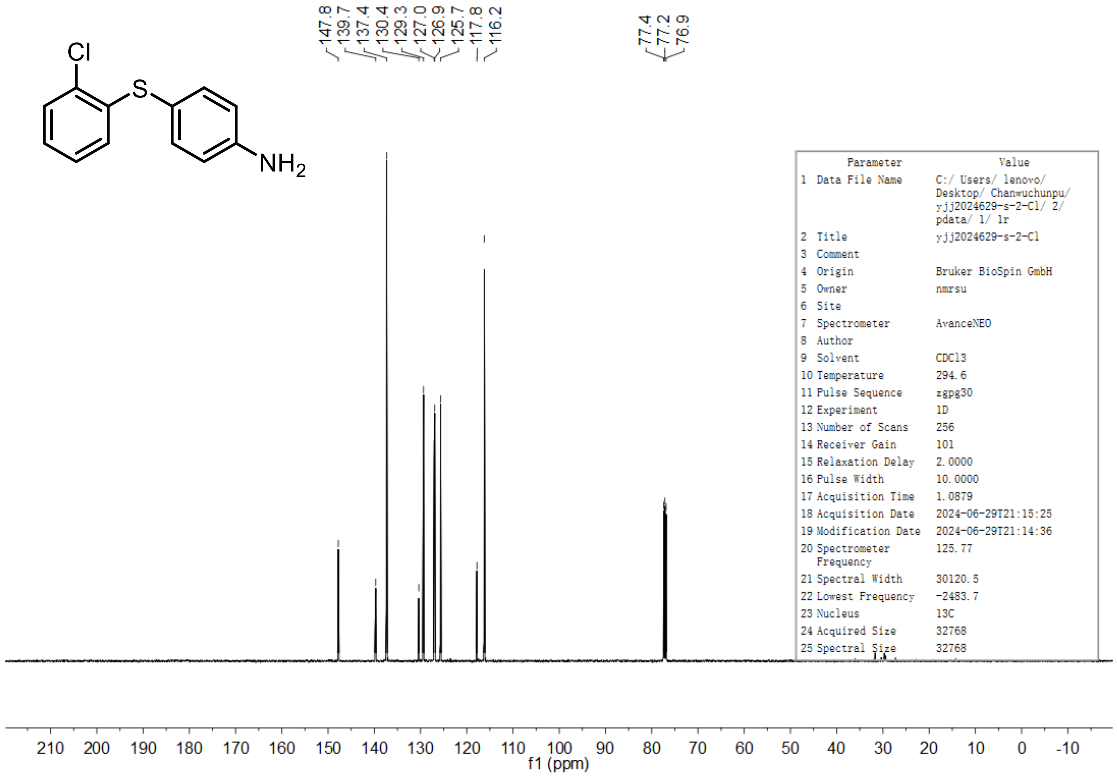

4-((2-bromophenyl)thio)aniline (**3fa**)

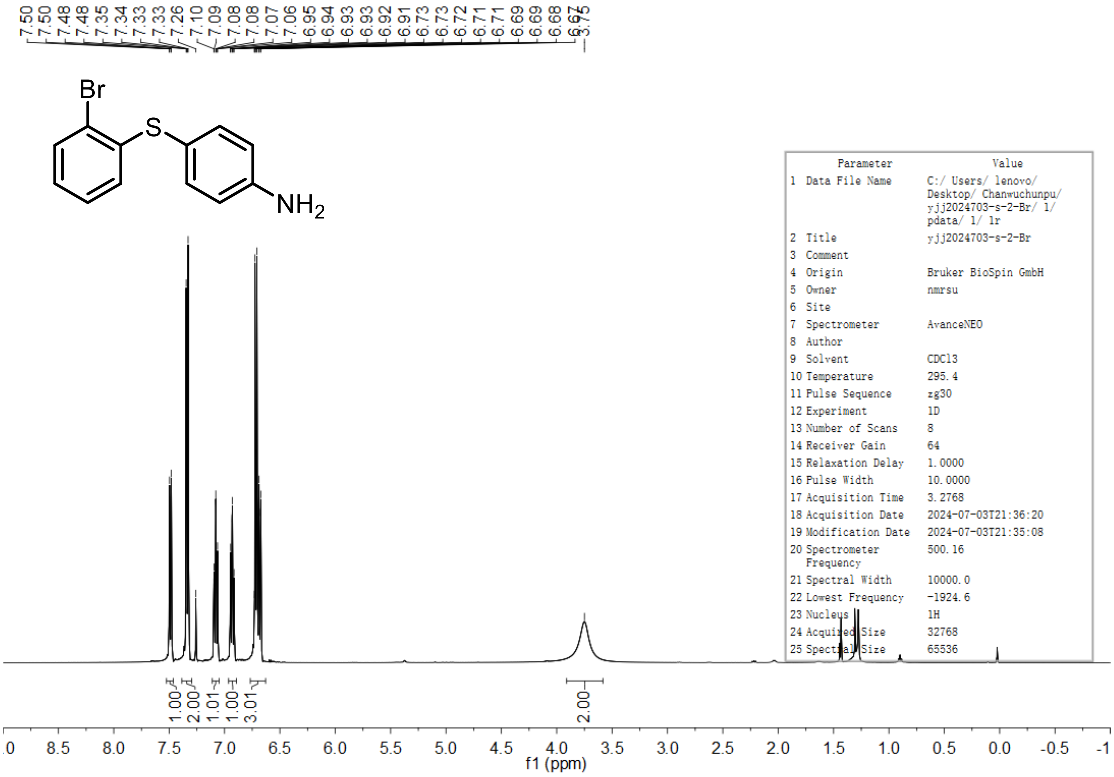

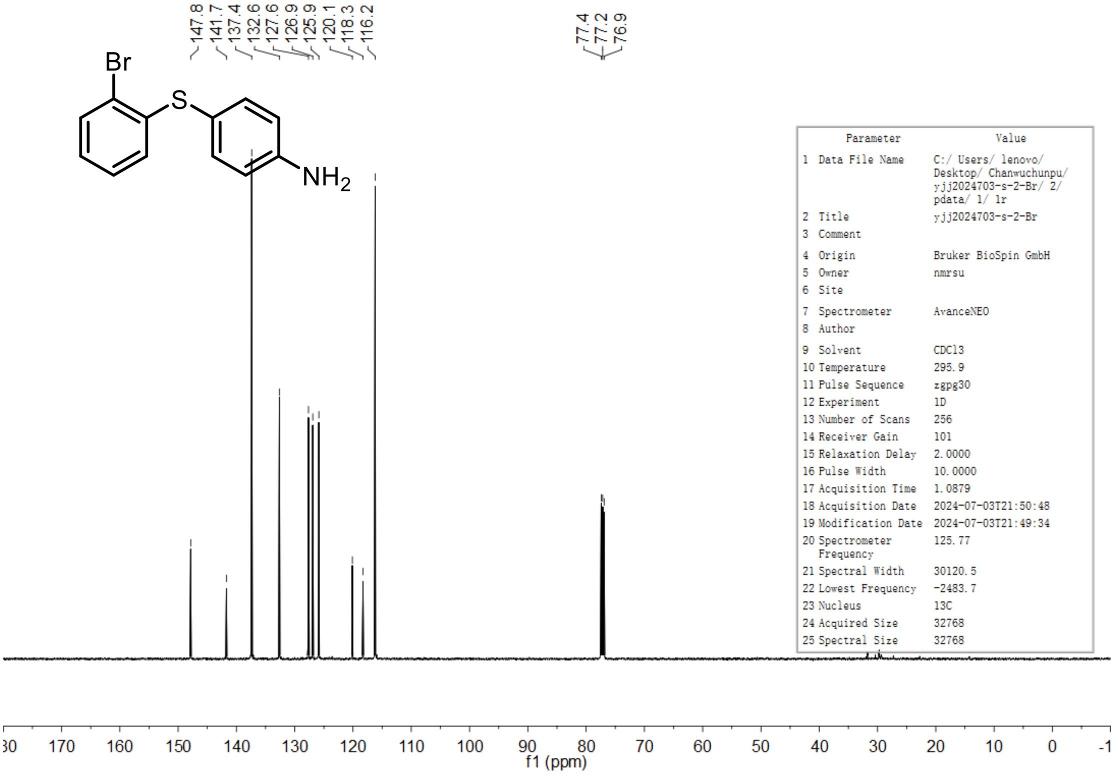

4-((3-chlorophenyl)thio)aniline (**3ga**)

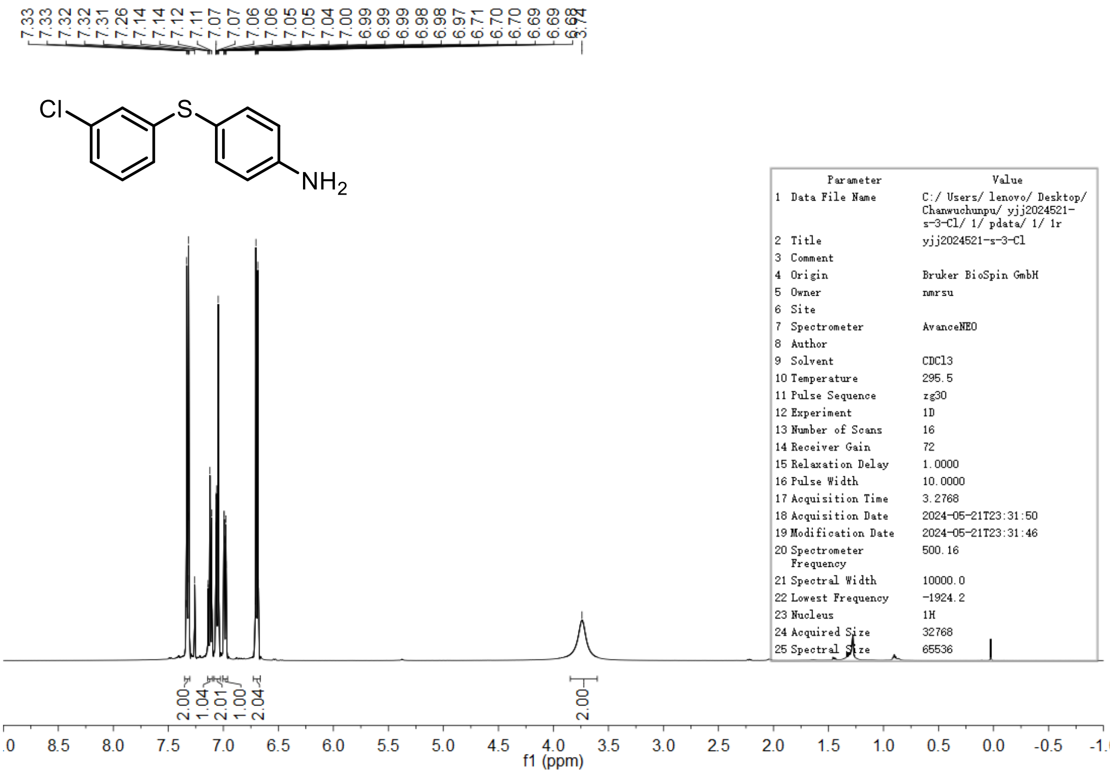

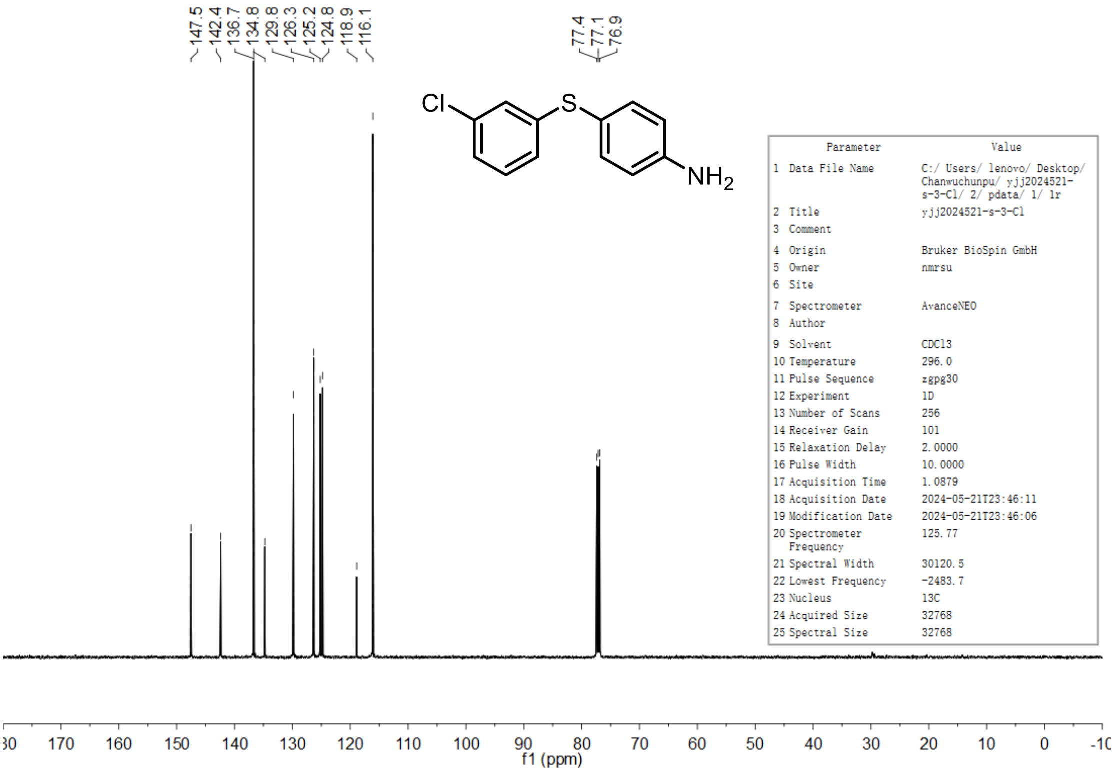

4-((4-fluorophenyl)thio)aniline (**3ha**)

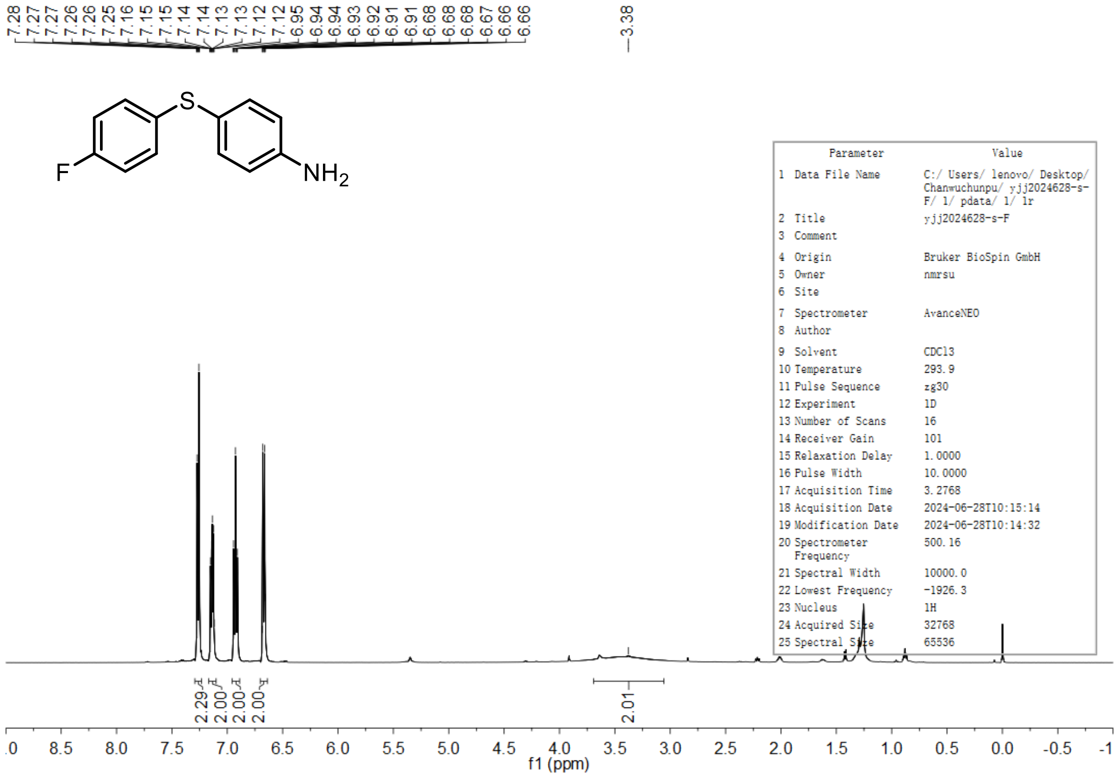

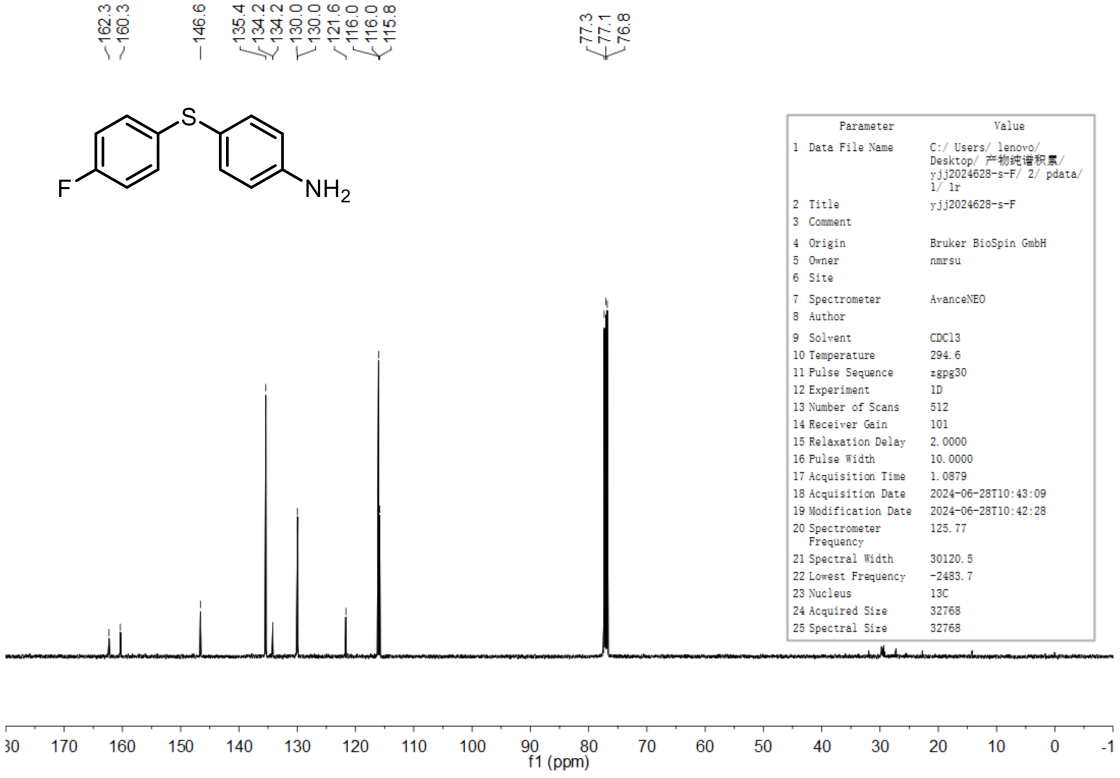

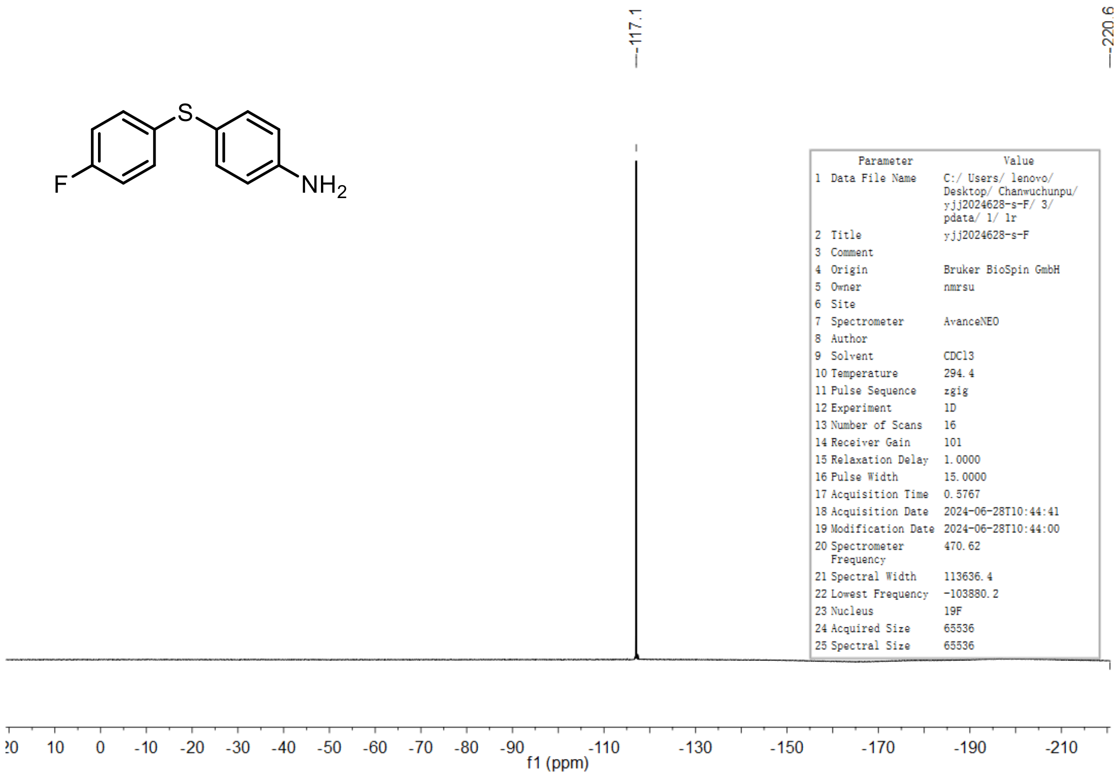

—117.1
—220.6
S
F
NH2
Parameter Value
1 Data File Name C:/ Users/ lenovo/ Desktop/ Chanwuchunpu/ yjj2024628-s-F/ 3/ pdata/ 1/ 1r
2 Title yjj2024628-s-F
3 Comment
4 Origin Bruker BioSpin GmbH
5 Owner nmrsu
6 Site
7 Spectrometer AvanceNEO
8 Author
9 Solvent CDCl3
10 Temperature 294.4
11 Pulse Sequence zgig
12 Experiment 1D
13 Number of Scans 16
14 Receiver Gain 101
15 Relaxation Delay 1.0000
16 Pulse Width 15.0000
17 Acquisition Time 0.5767
18 Acquisition Date 2024-06-28T10:44:41
19 Modification Date 2024-06-28T10:44:00
20 Spectrometer Frequency 470.62
21 Spectral Width 113636.4
22 Lowest Frequency -103880.2
23 Nucleus 19F
24 Acquired Size 65536
25 Spectral Size 65536
20 10 0 -10 -20 -30 -40 -50 -60 -70 -80 -90 -110 -130 -150 -170 -190 -210
f1 (ppm)

4-(p-tolylthio)aniline (**3ia**)

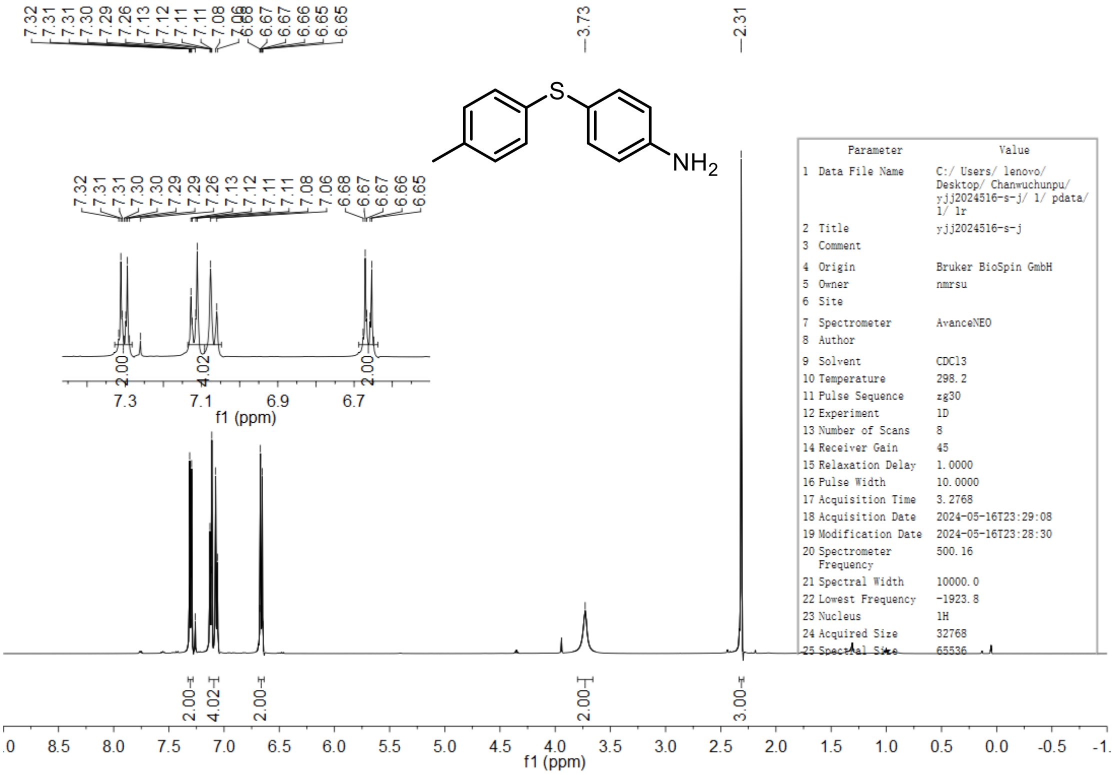

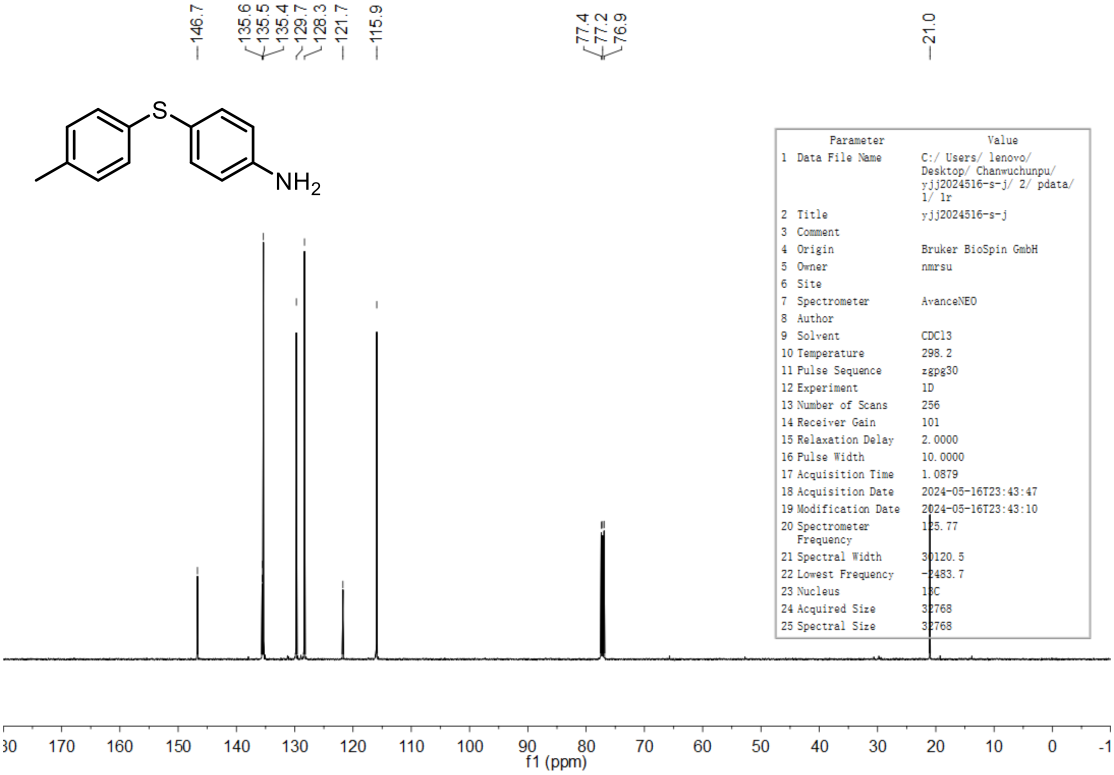

4-(o-tolylthio)aniline (**3ja**)

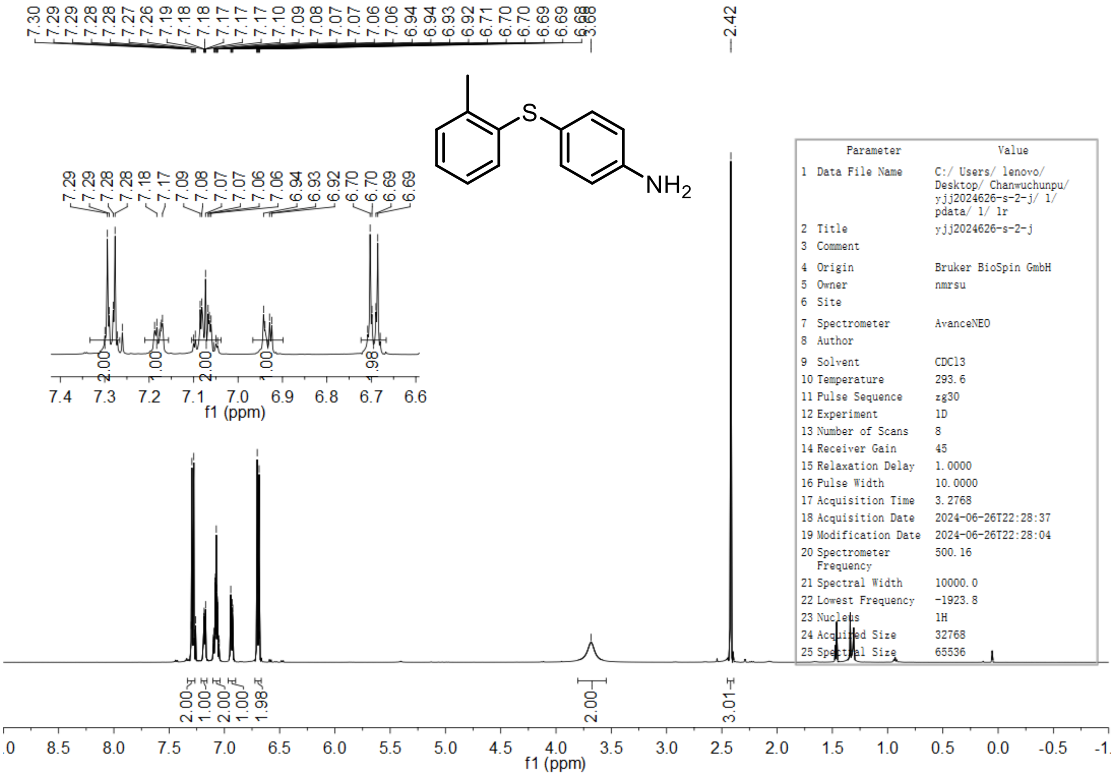

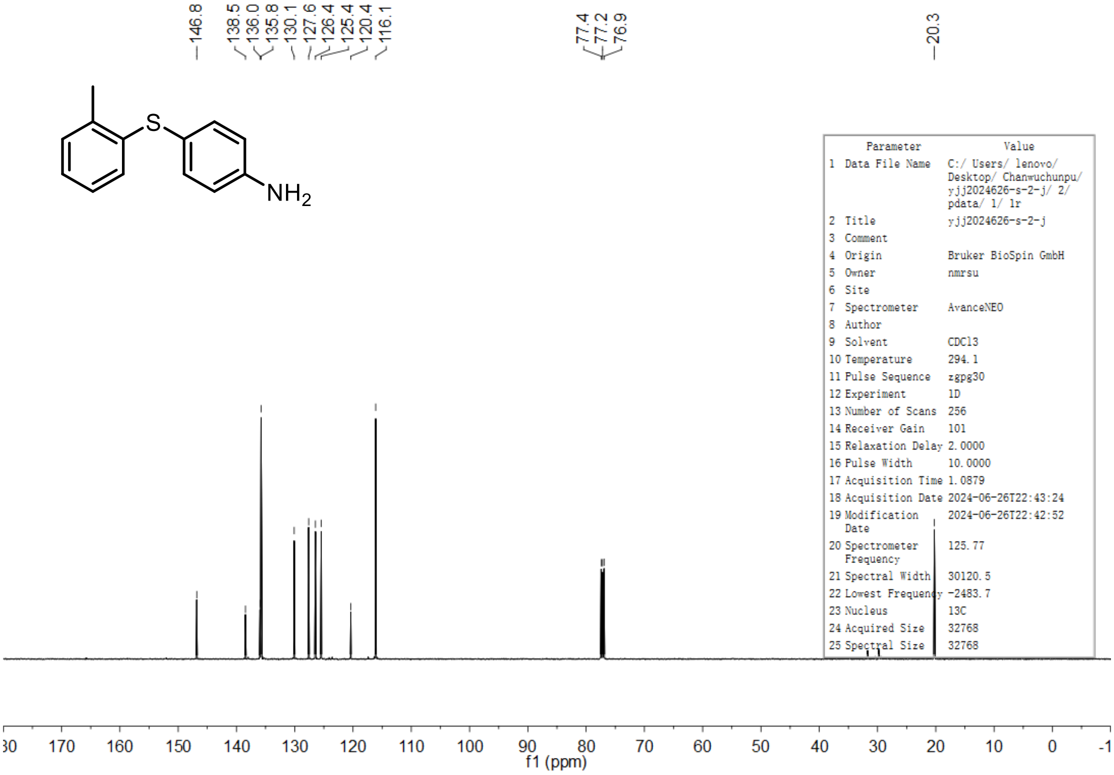

4-(m-tolylthio)aniline (**3ka**)

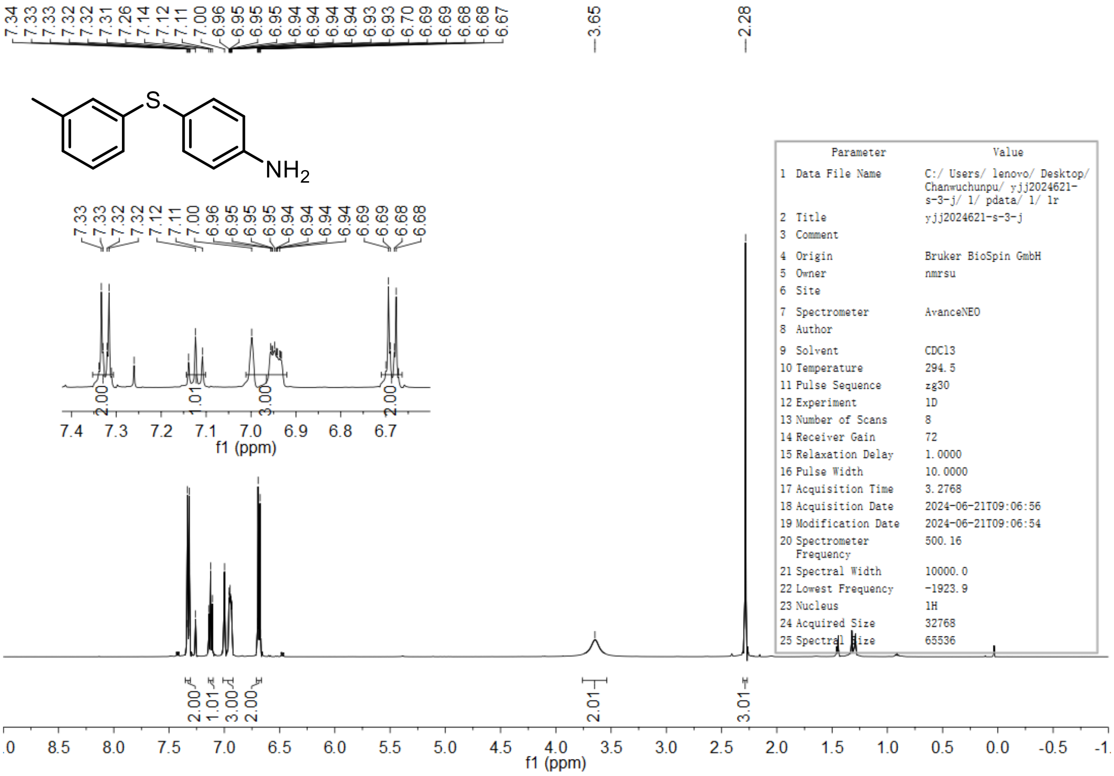

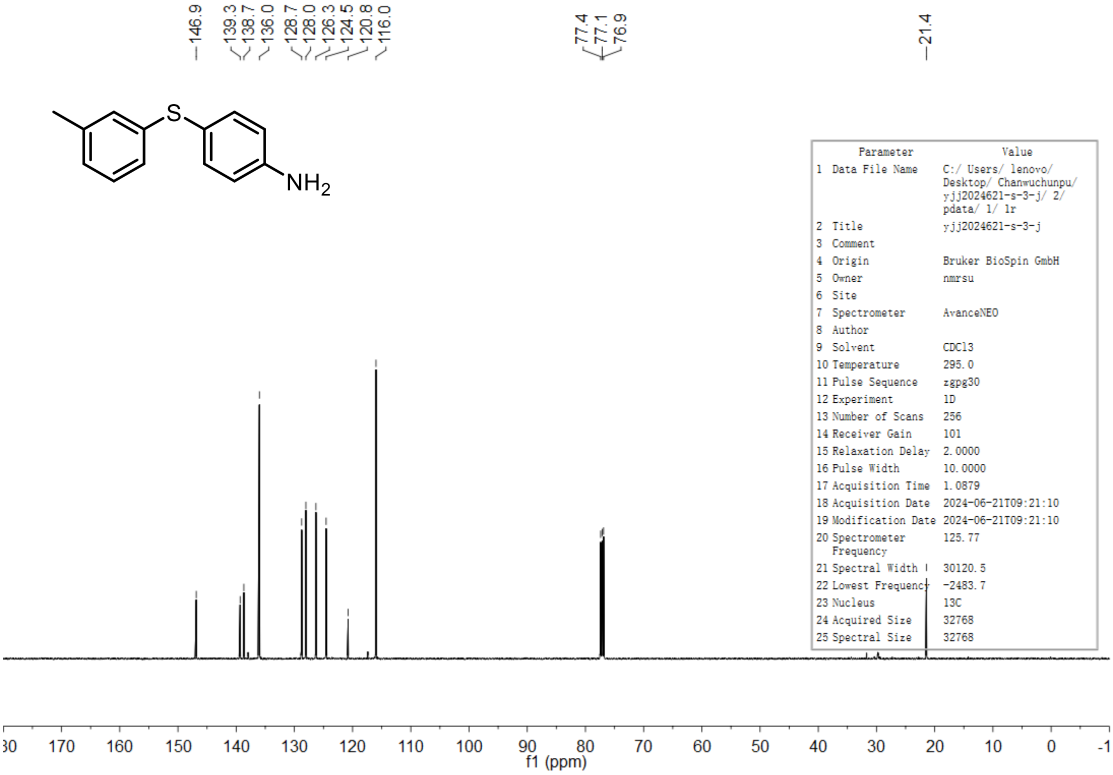

4-((4-methoxyphenyl)thio)aniline (**3la**)

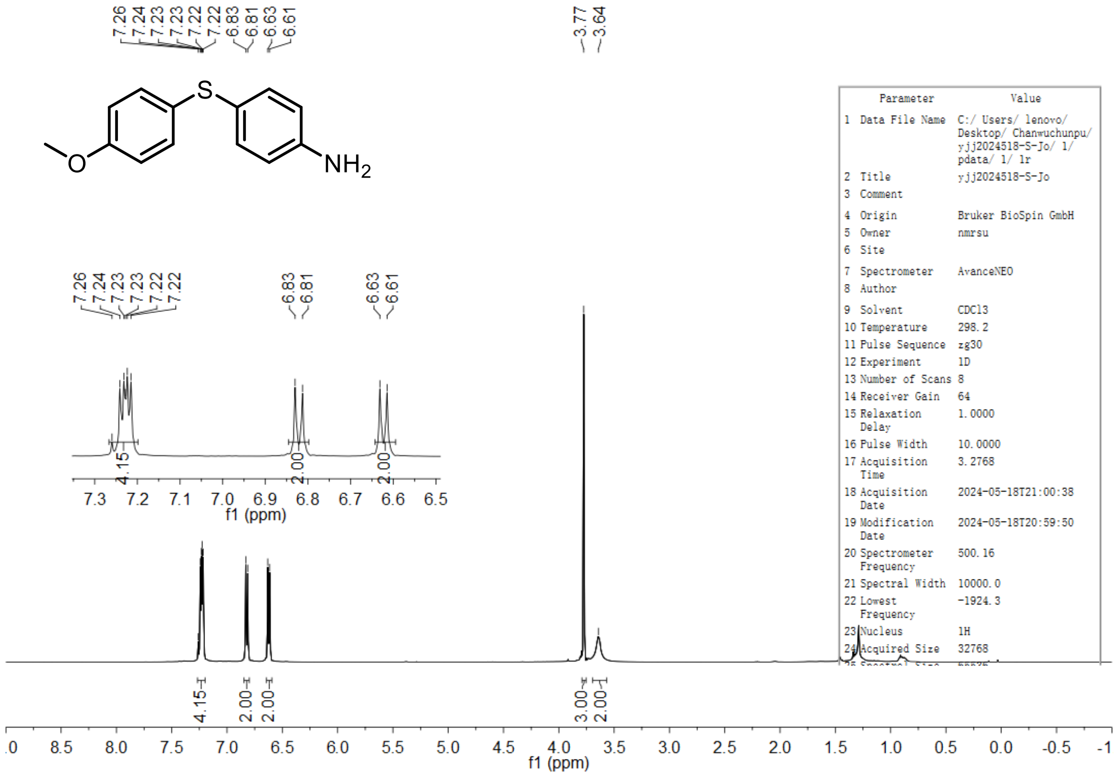

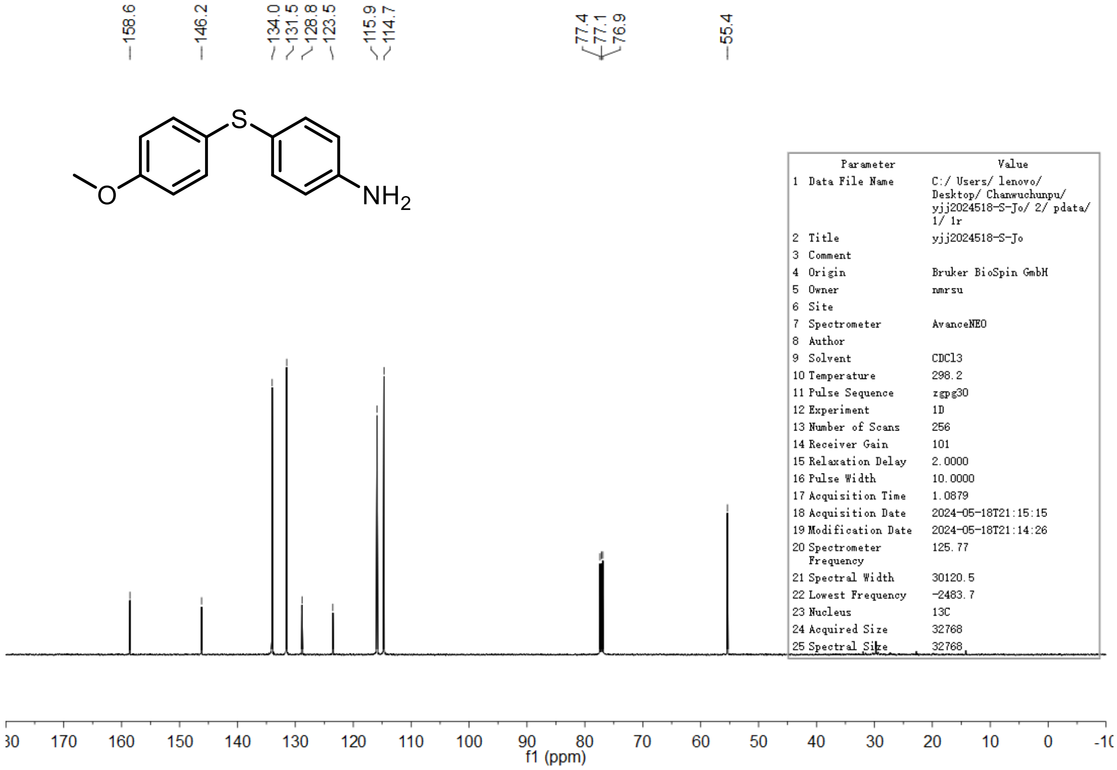

4-((3-methoxyphenyl)thio)aniline (**3ma**)

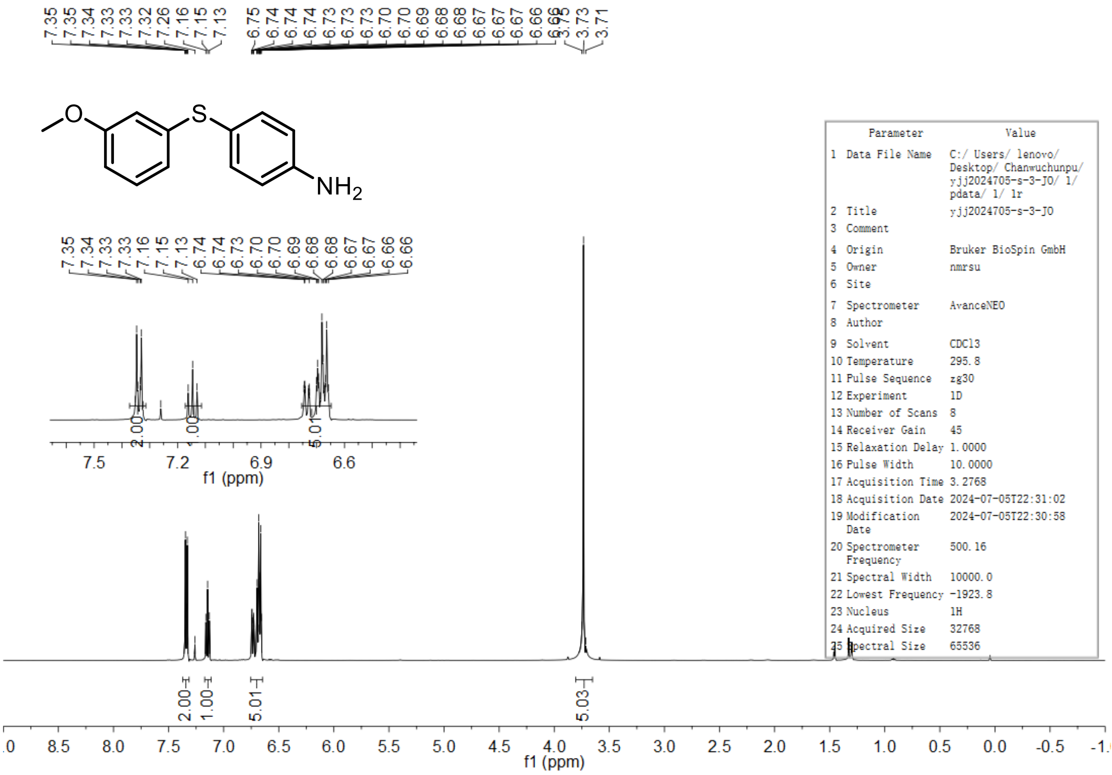

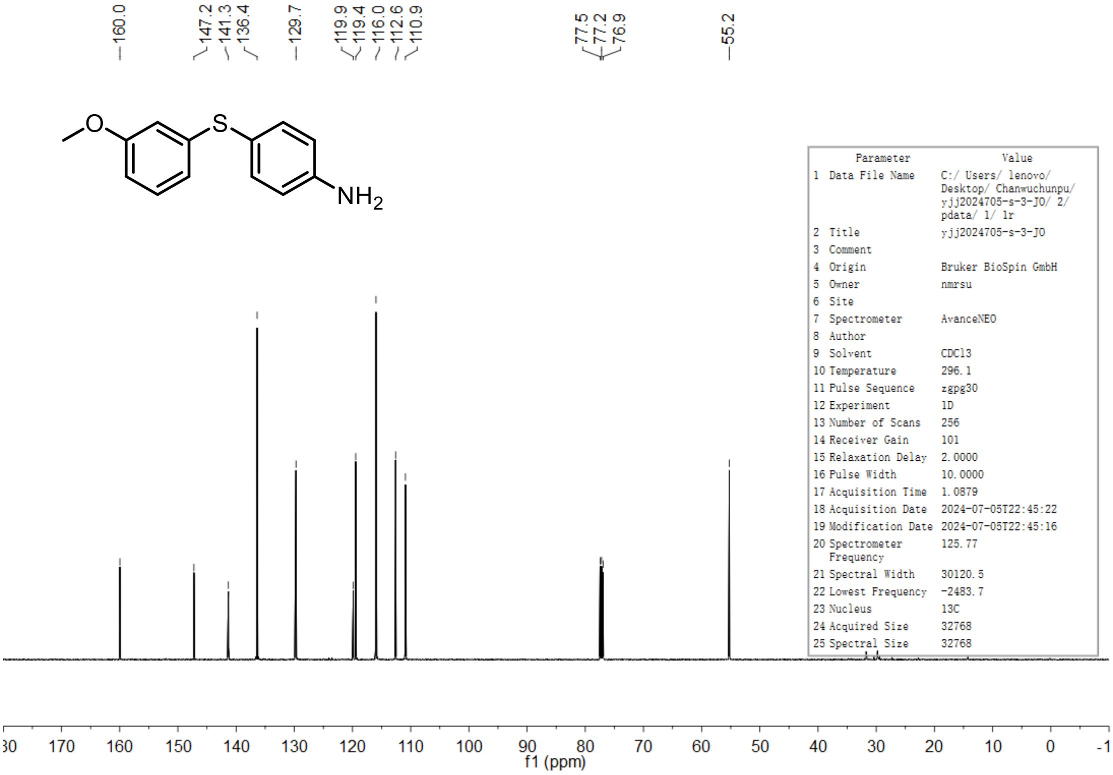

4-((2-methylfuran-3-yl)thio)aniline (**3na**)

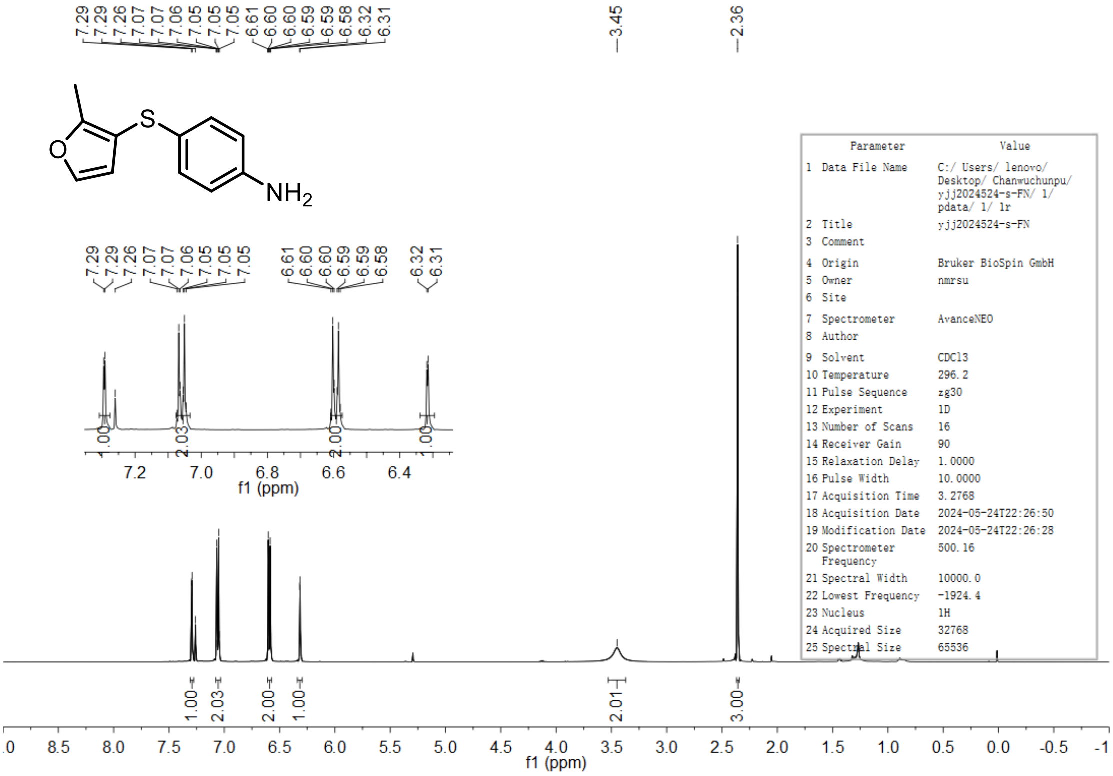

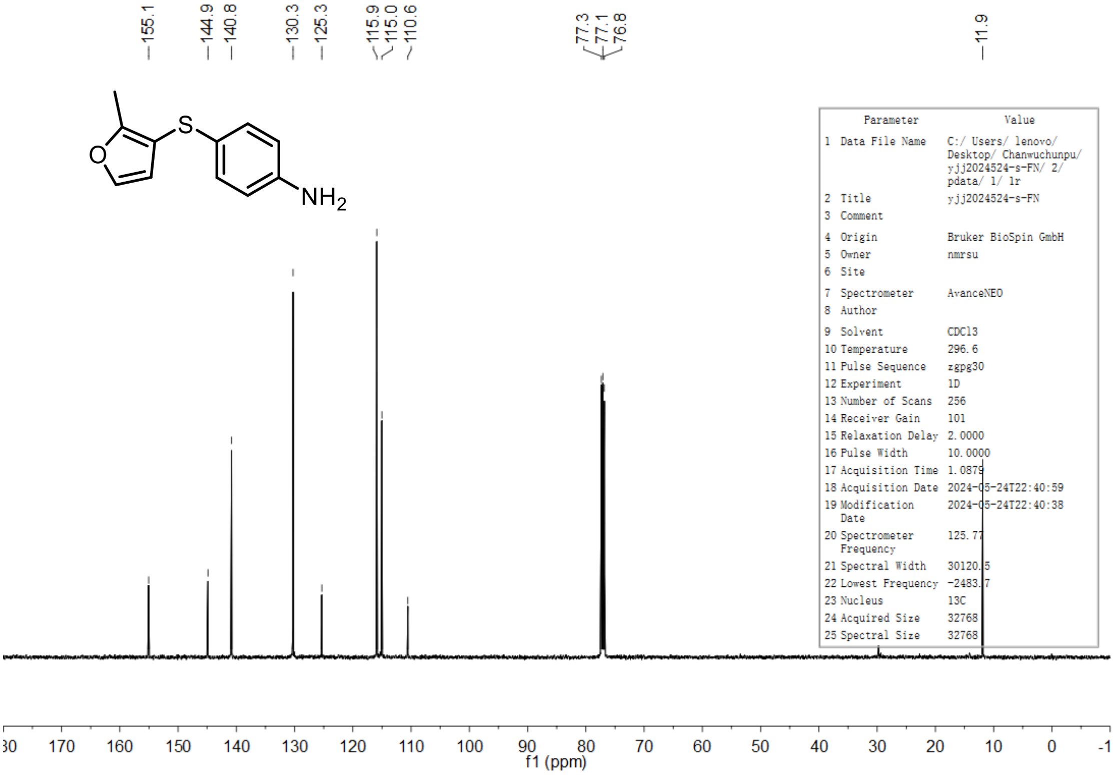

2-methyl-4-(phenylthio)aniline (**3ab**)

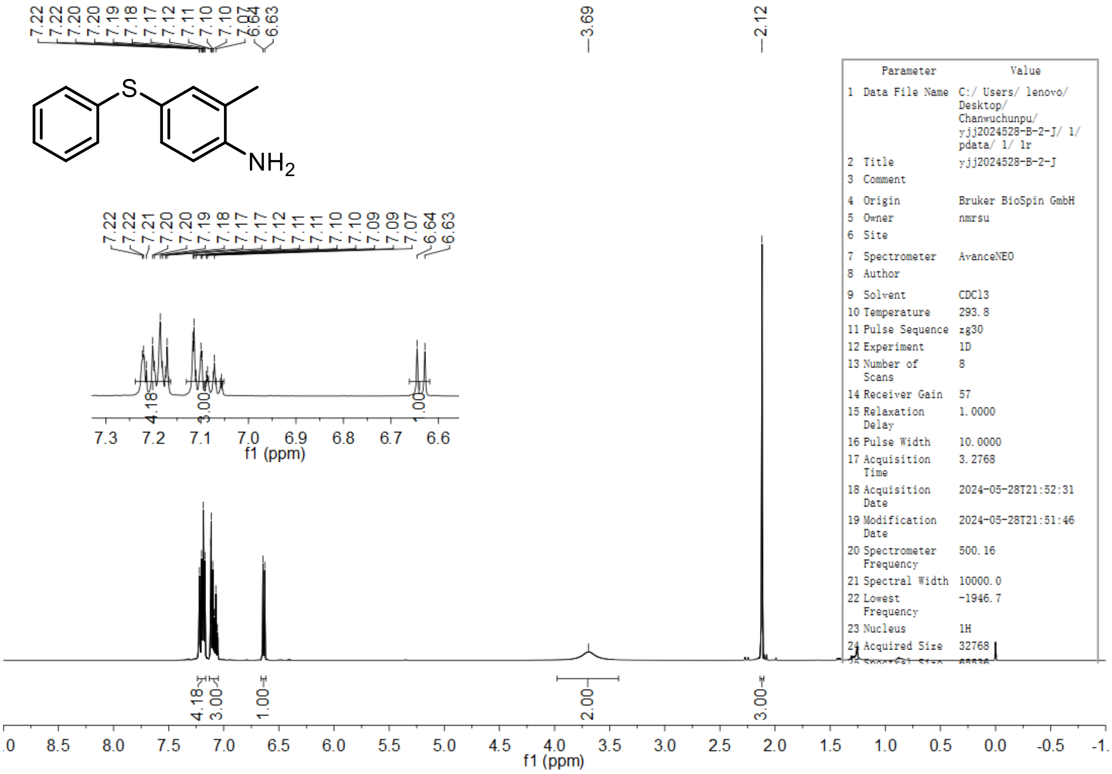

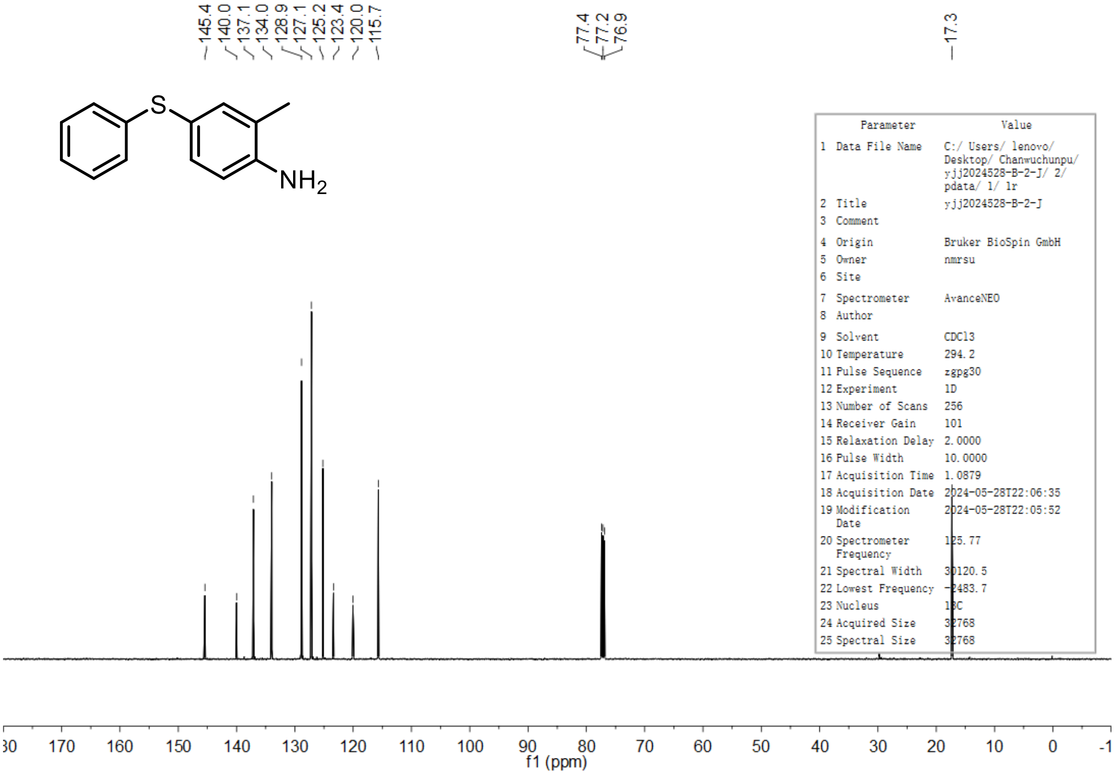

3-methyl-4-(phenylthio)aniline (**3ac**)

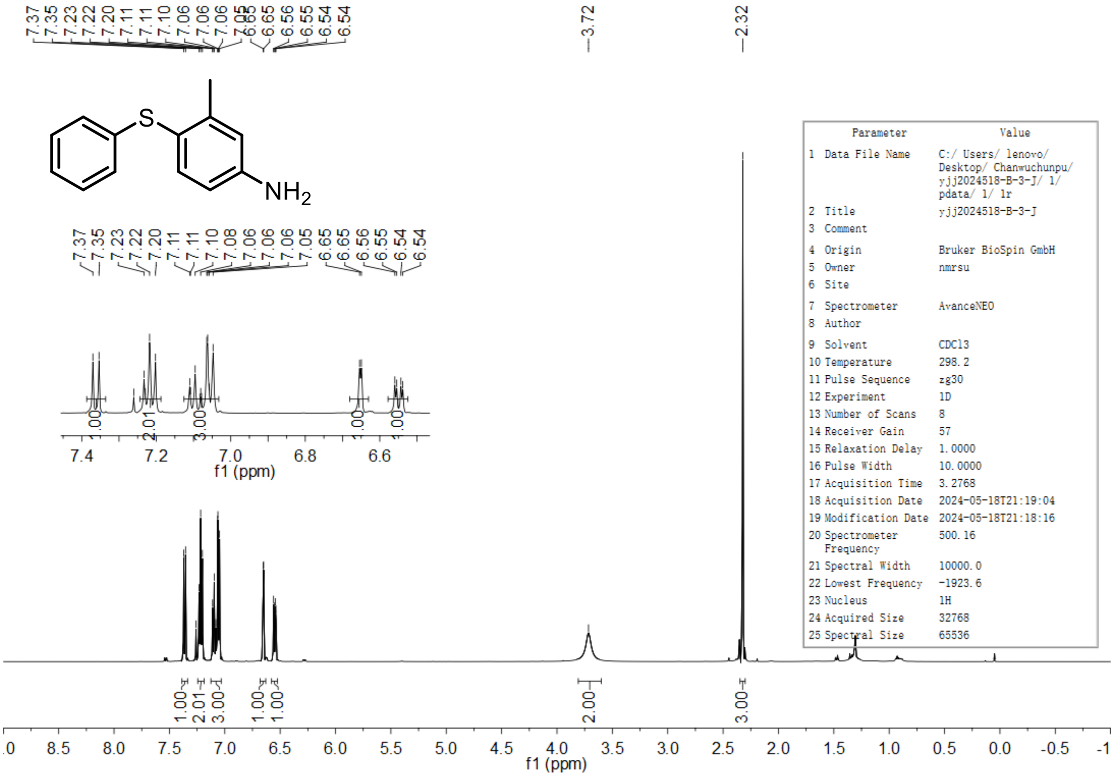

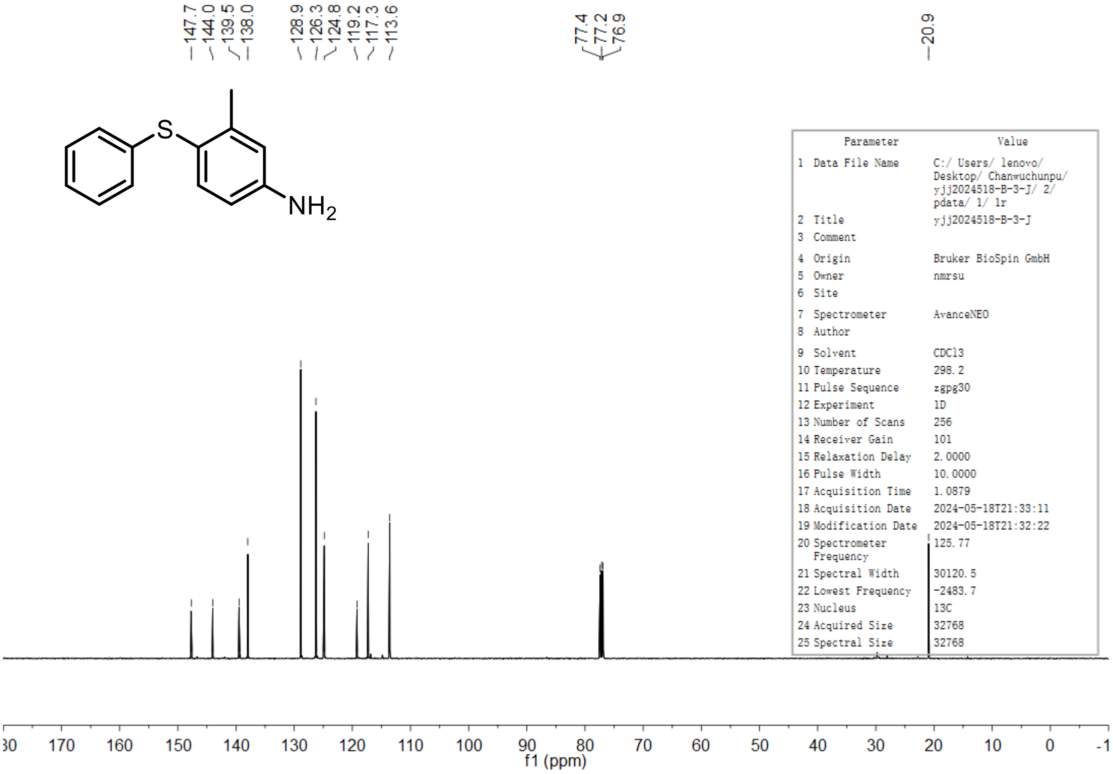

3-bromo-4-(phenylthio)aniline (**3ad**)

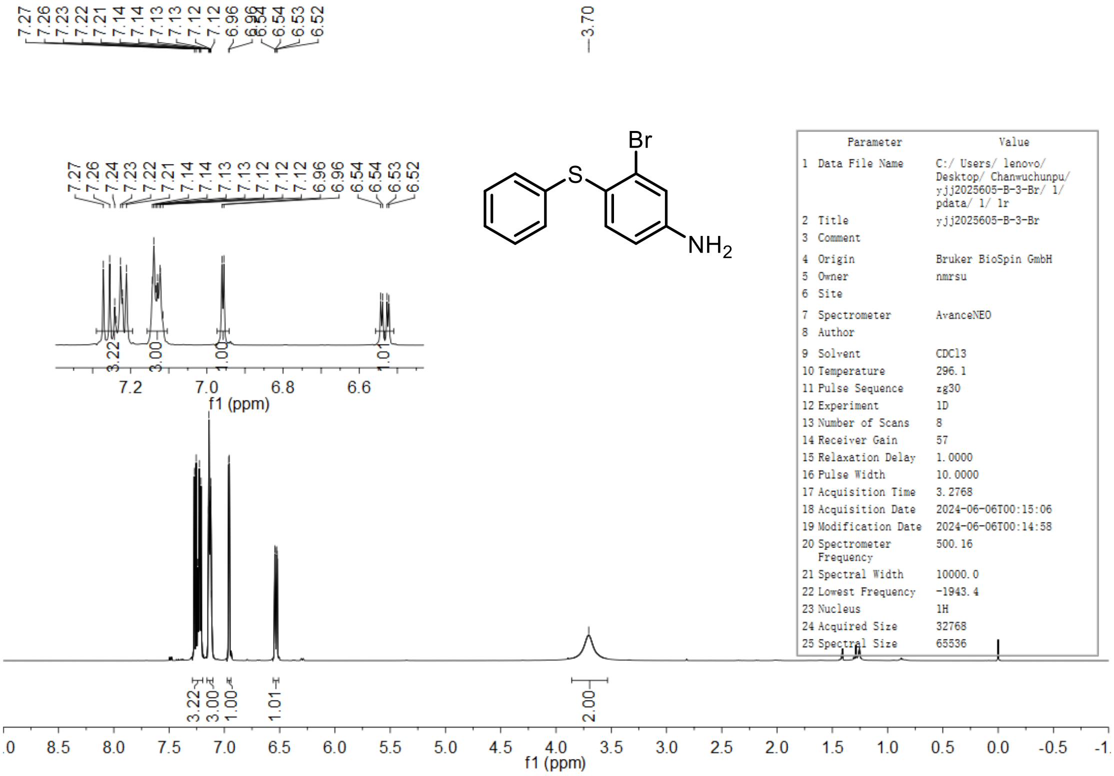

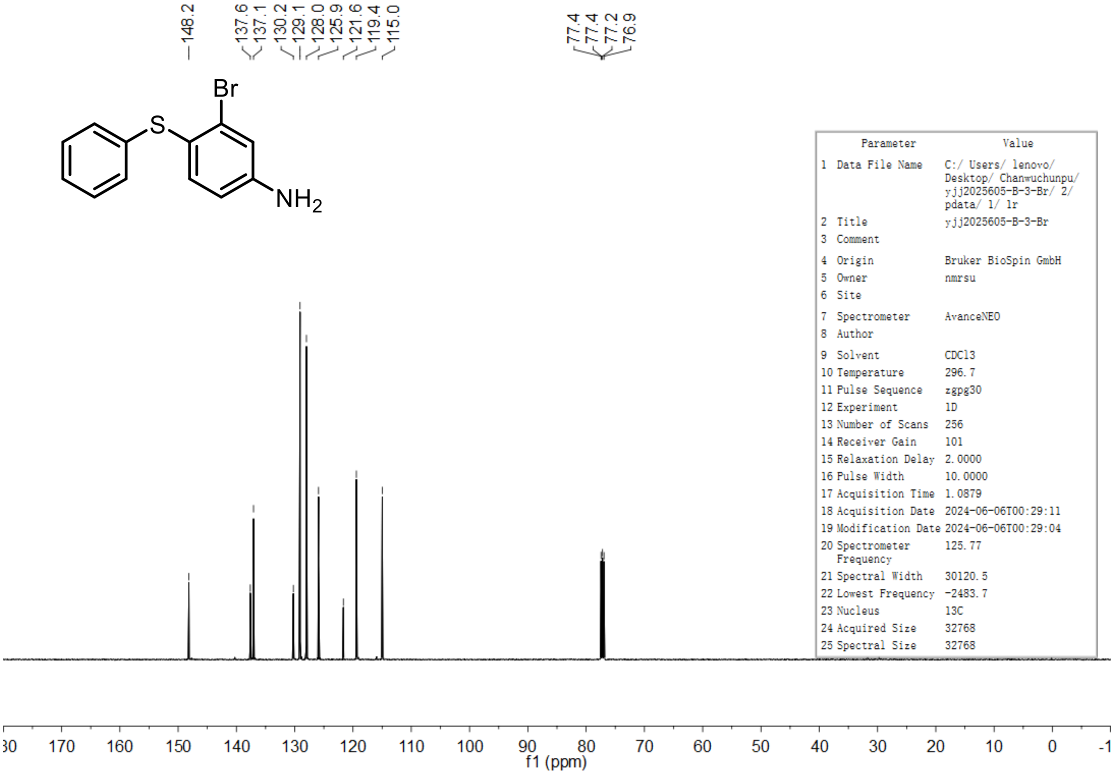

3-chloro-4-(phenylthio)aniline (**3ae**)

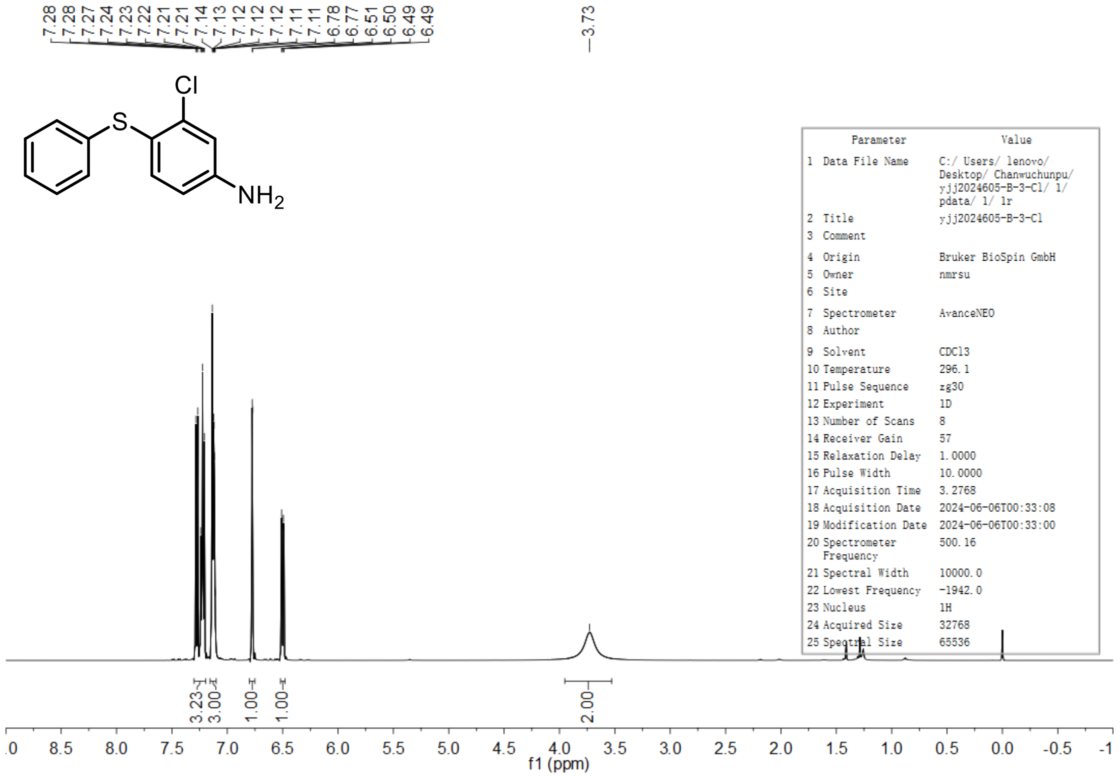

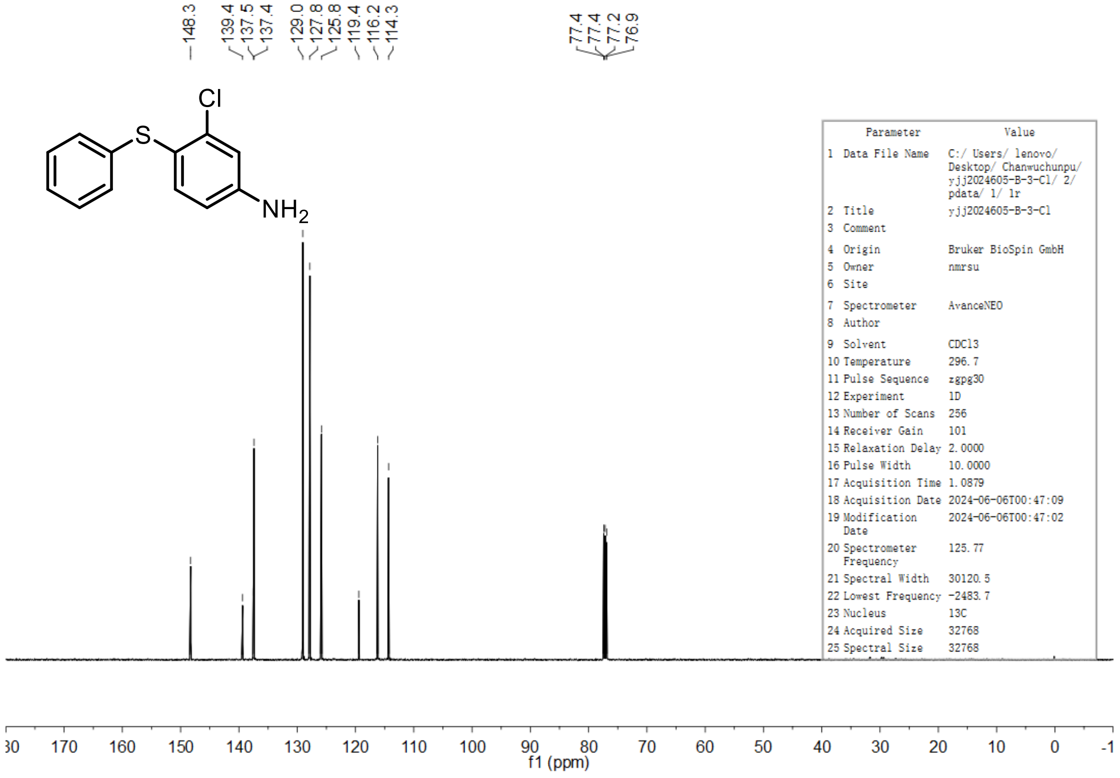

3-fluoro-4-(phenylthio)aniline (**3af**)

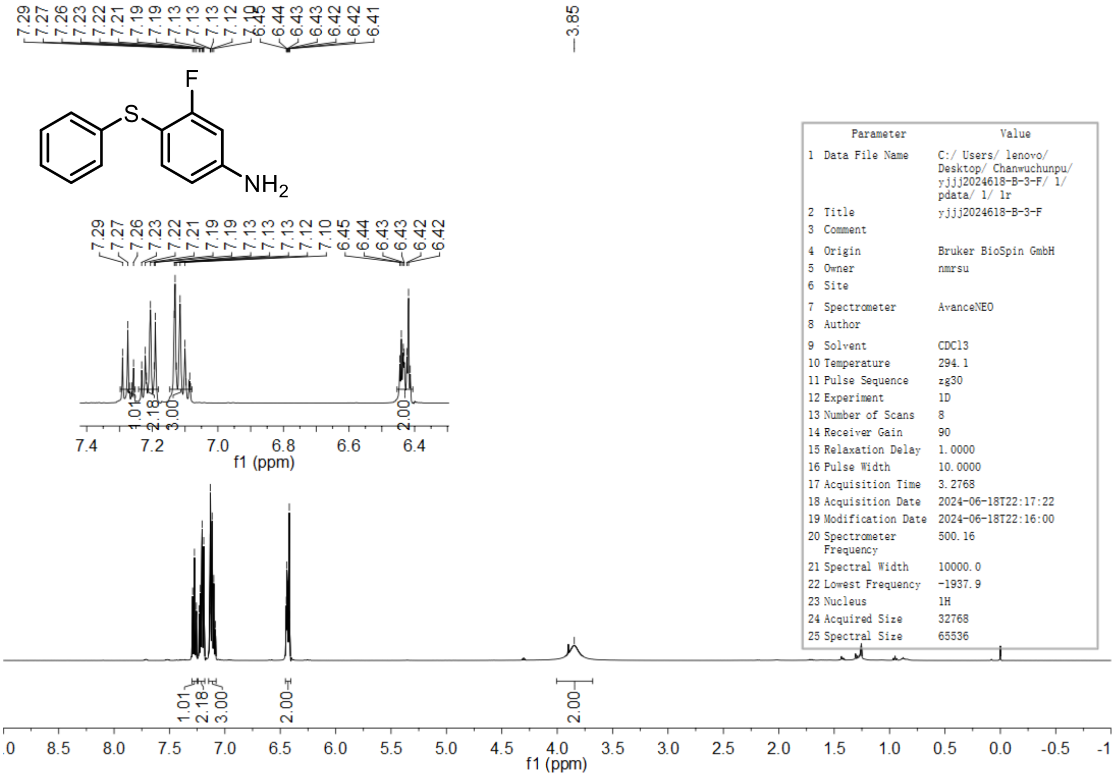

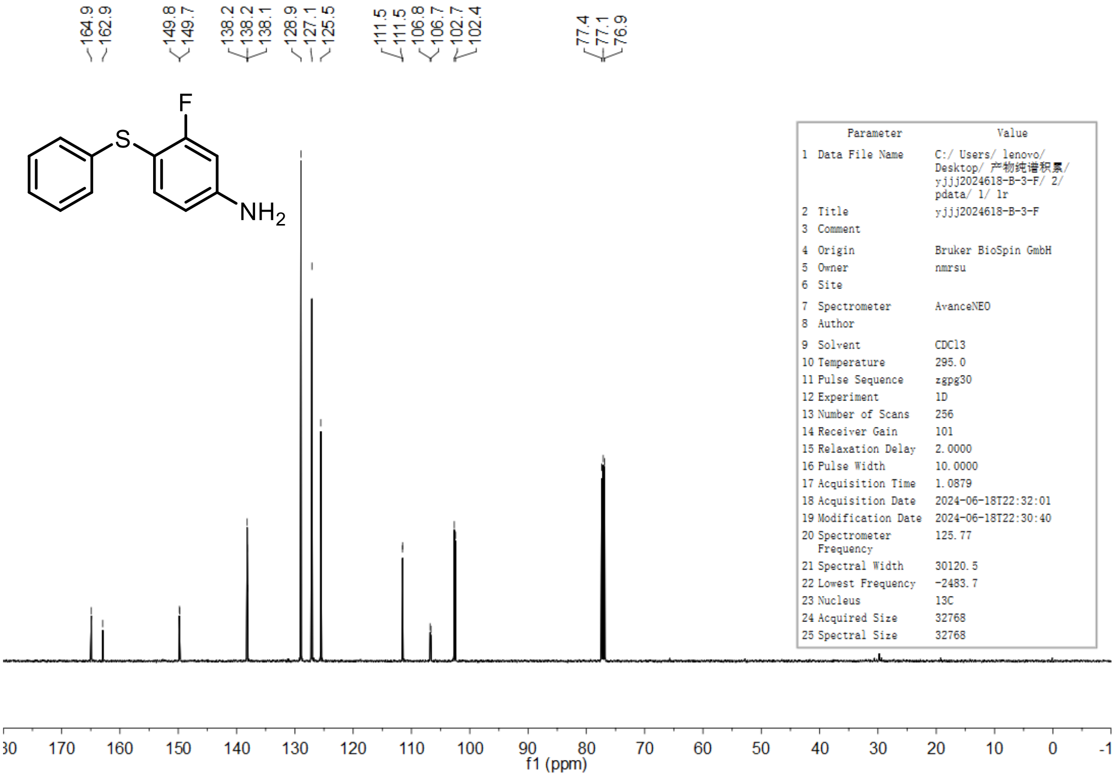

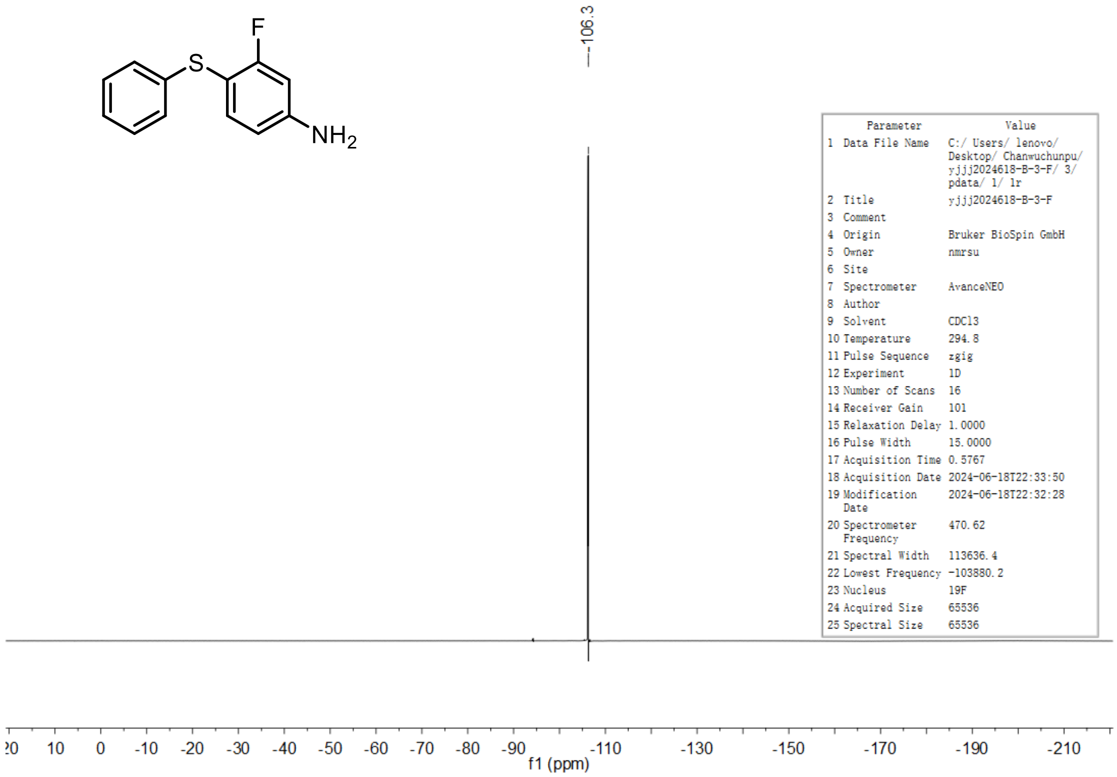
F
S
NH2
-106.3
Parameter Value
1 Data File Name C:/ Users/ lenovo/ Desktop/ Chanwuchunpu/ yjjj2024618-B-3-F/ 3/ pdata/ 1/ 1r
2 Title yjjj2024618-B-3-F
3 Comment
4 Origin Bruker BioSpin GmbH
5 Owner nmrsu
6 Site
7 Spectrometer AvanceNEO
8 Author
9 Solvent CDCl3
10 Temperature 294.8
11 Pulse Sequence zgig
12 Experiment 1D
13 Number of Scans 16
14 Receiver Gain 101
15 Relaxation Delay 1.0000
16 Pulse Width 15.0000
17 Acquisition Time 0.5767
18 Acquisition Date 2024-06-18T22:33:50
19 Modification Date 2024-06-18T22:32:28
20 Spectrometer Frequency 470.62
21 Spectral Width 113636.4
22 Lowest Frequency -103880.2
23 Nucleus 19F
24 Acquired Size 65536
25 Spectral Size 65536
20 10 0 -10 -20 -30 -40 -50 -60 -70 -80 -90 -110 -130 -150 -170 -190 -210
f1 (ppm)

3-methoxy-4-(phenylthio)aniline (**3ag**)

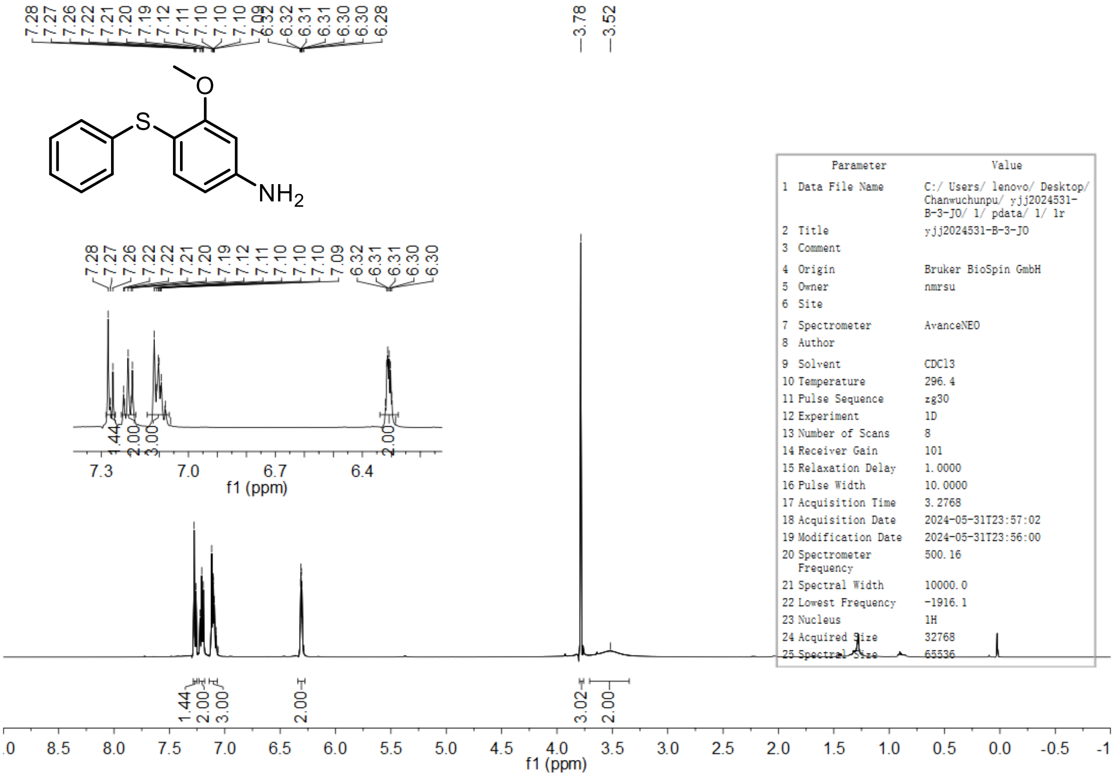

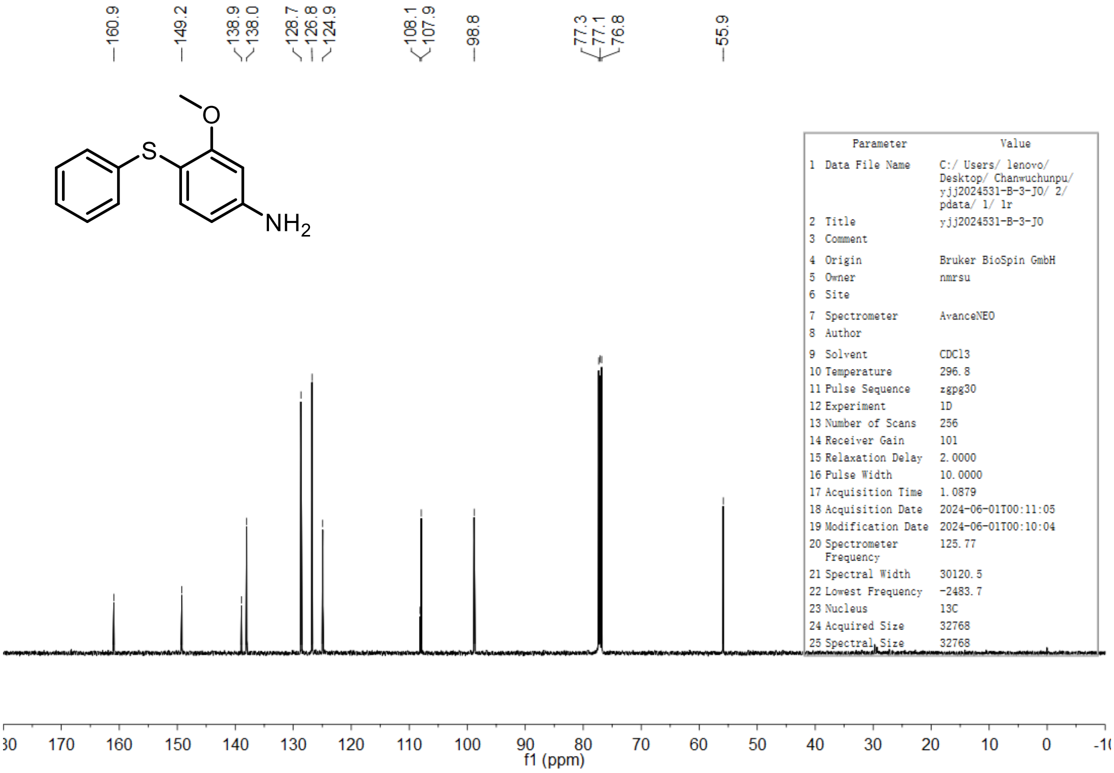

2-bromo-4-(phenylthio)aniline (**3ah**)

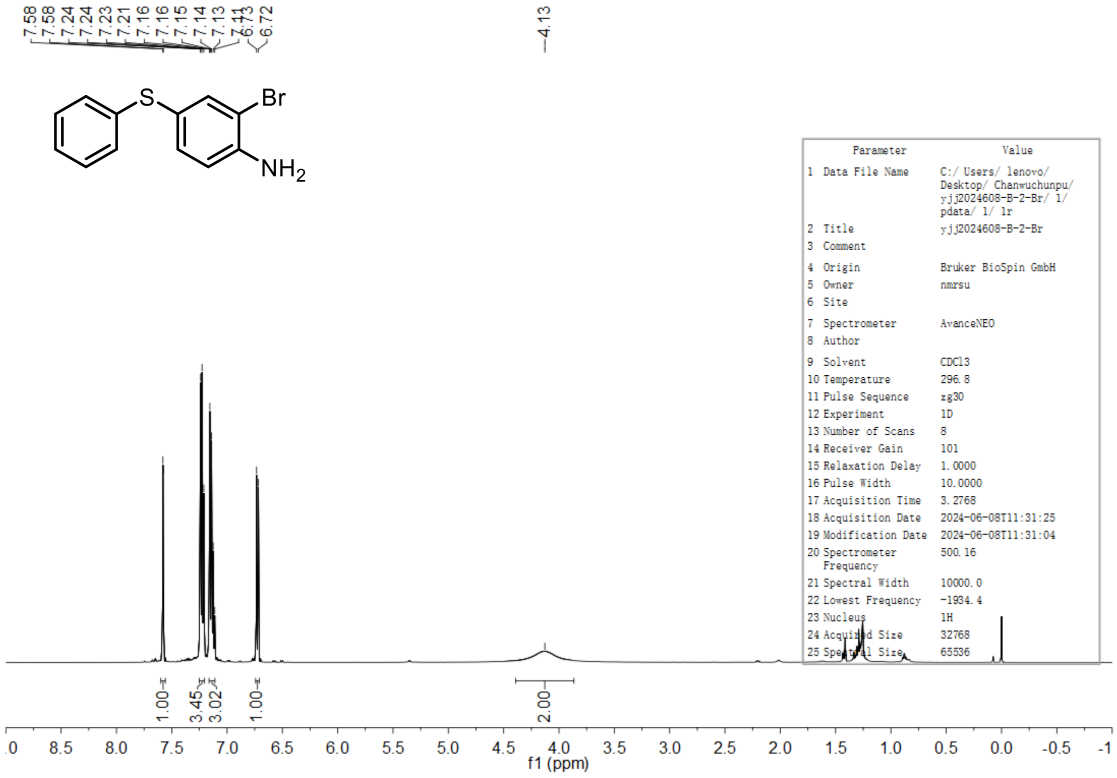

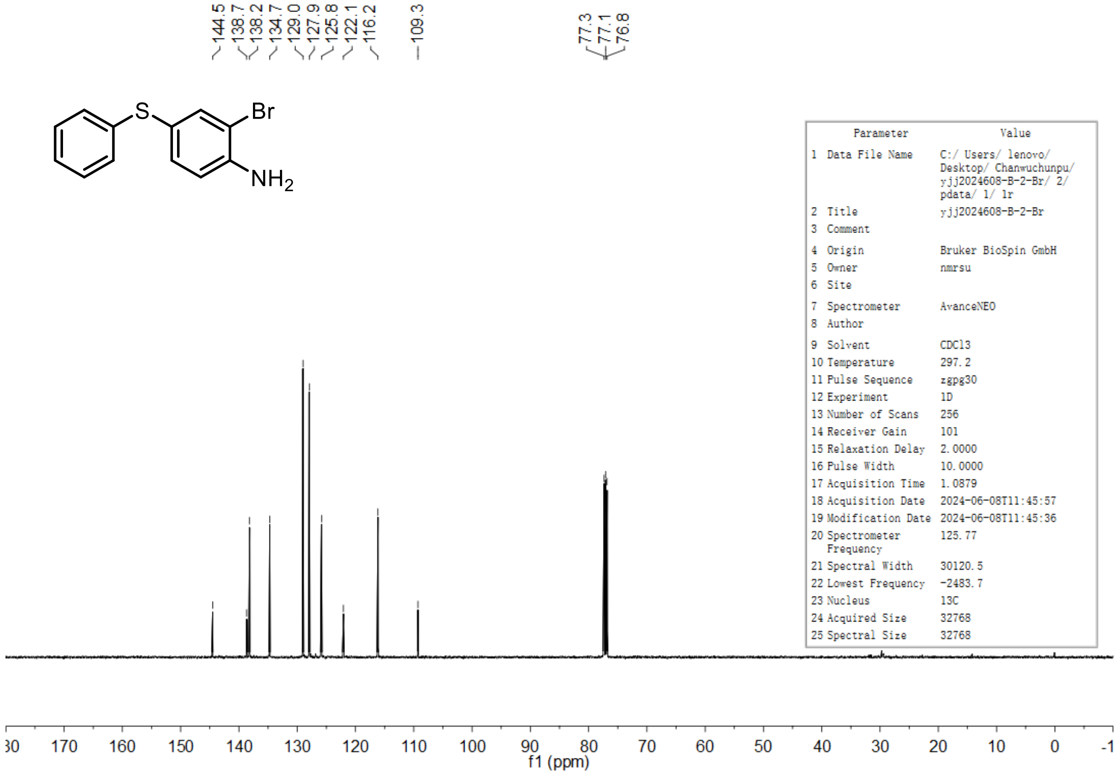

2-chloro-4-(phenylthio)aniline (**3ai**)

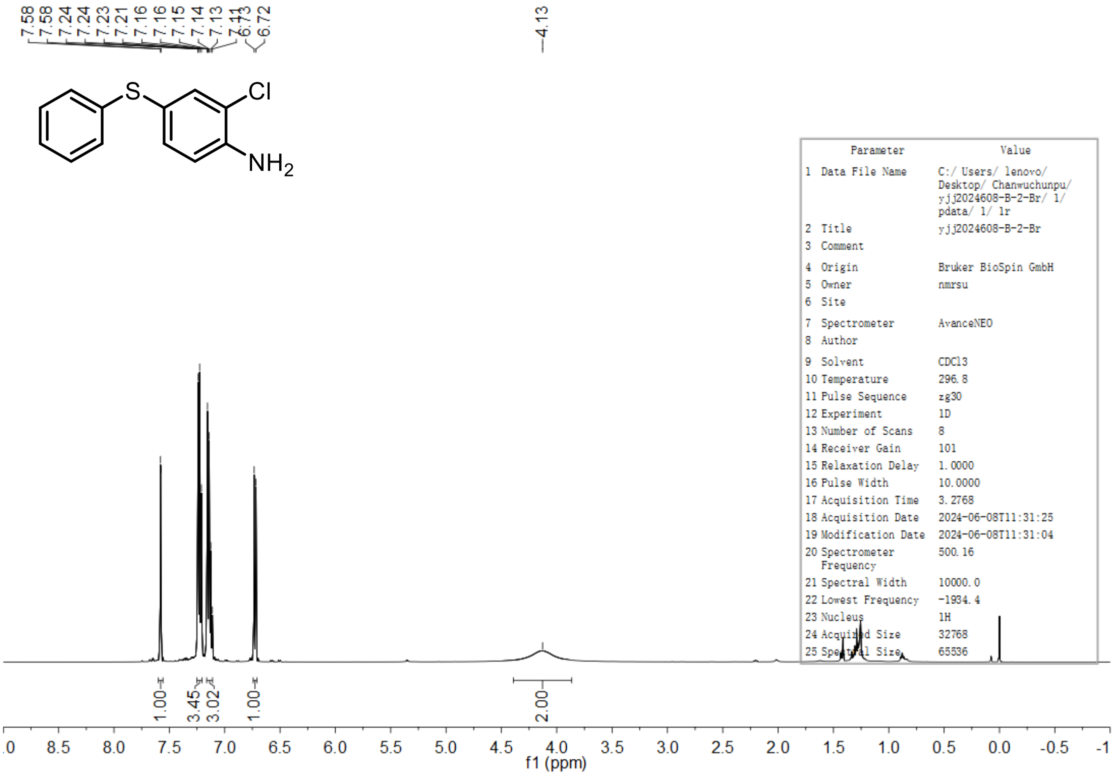

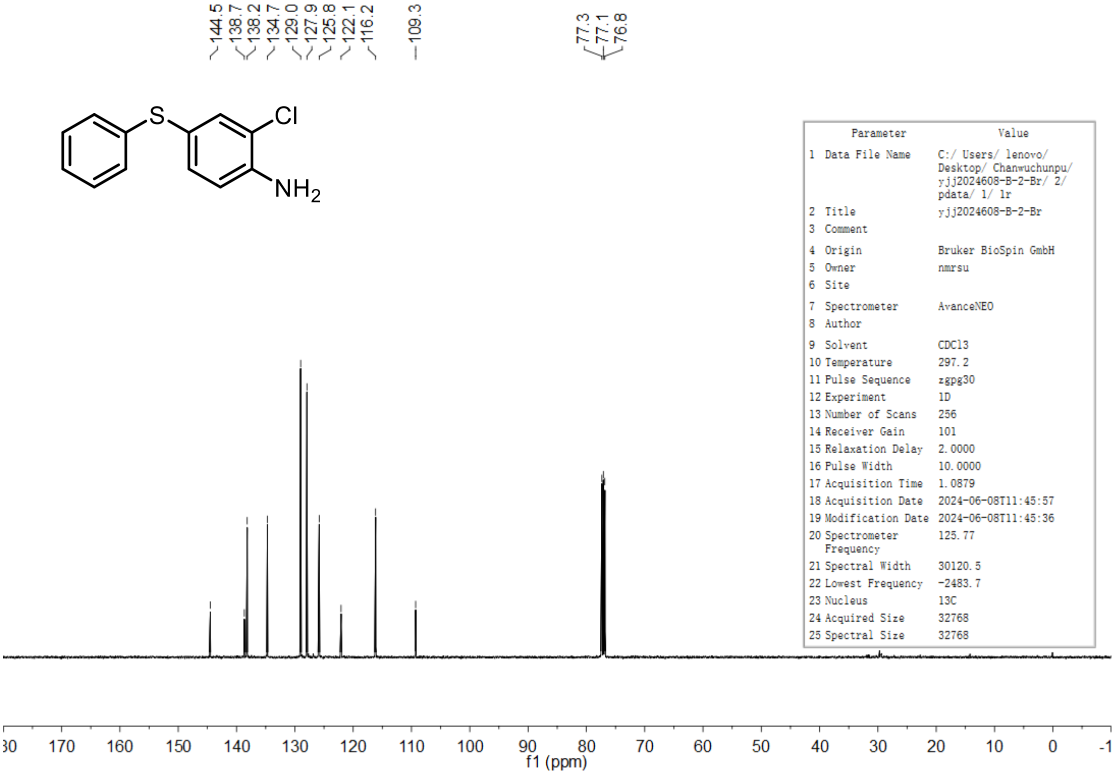

2-(tert-butyl)-4-(phenylthio)aniline (**3aj**)

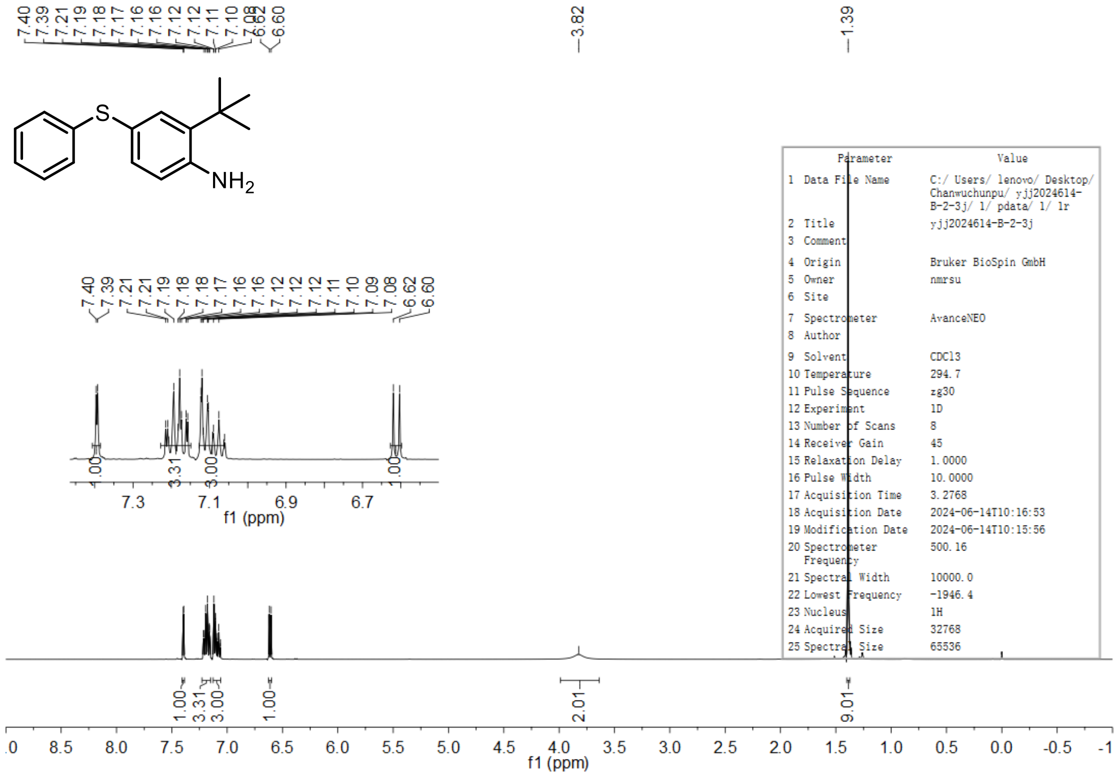

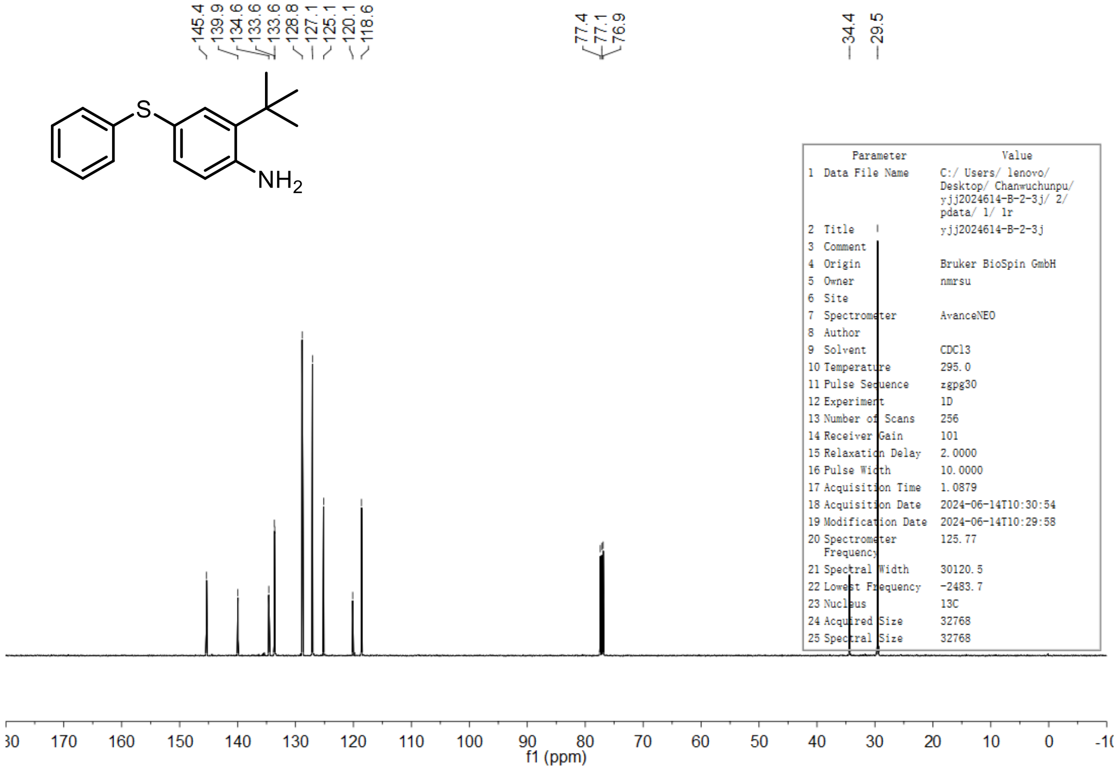

2-methoxy-4-(phenylthio)aniline (**3ak**)

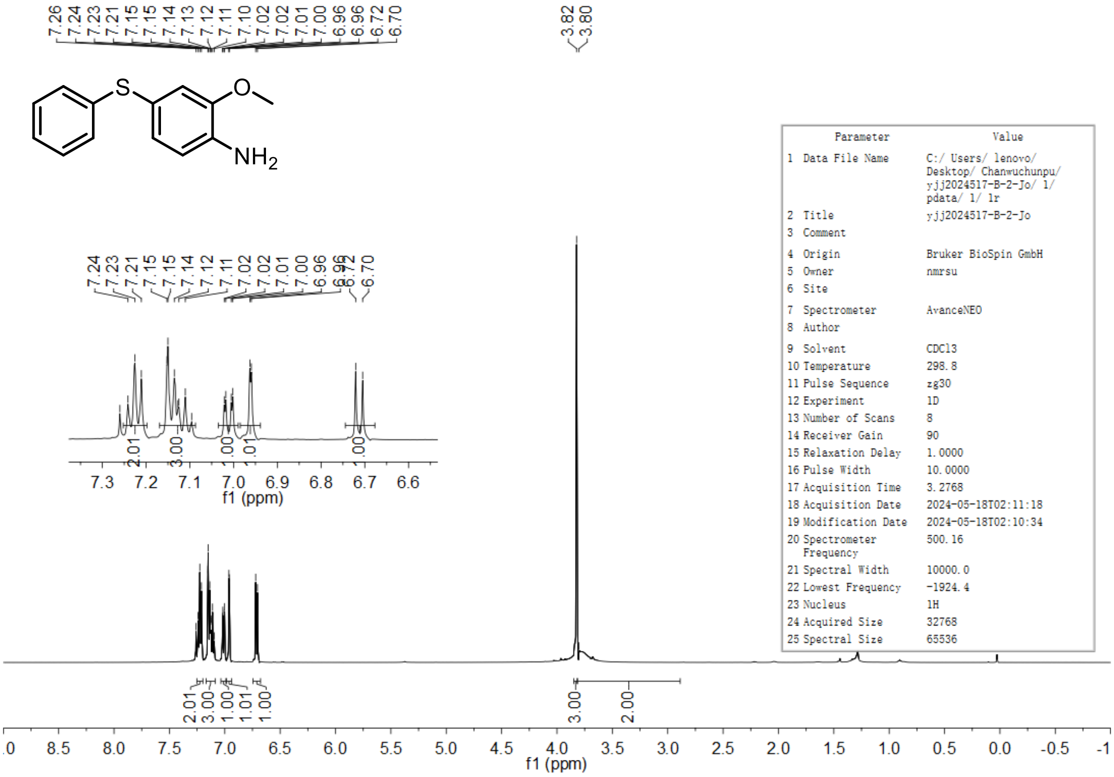

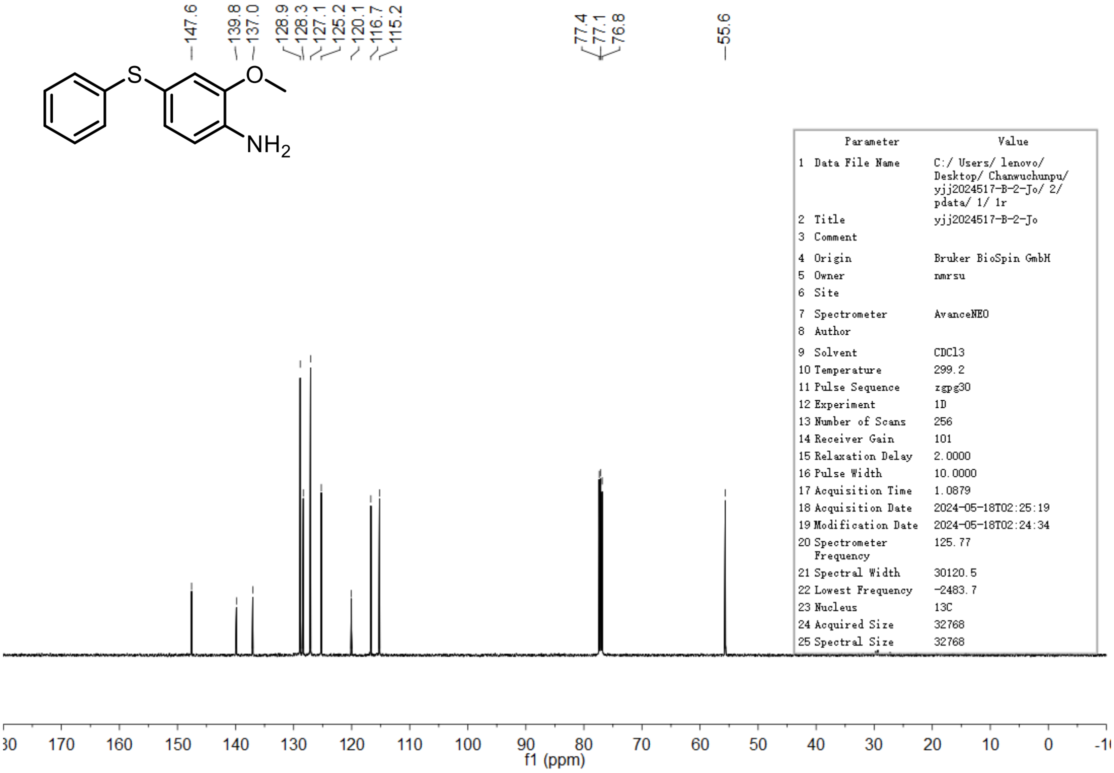

2,6-dimethyl-4-(phenylthio)aniline (**3al**)

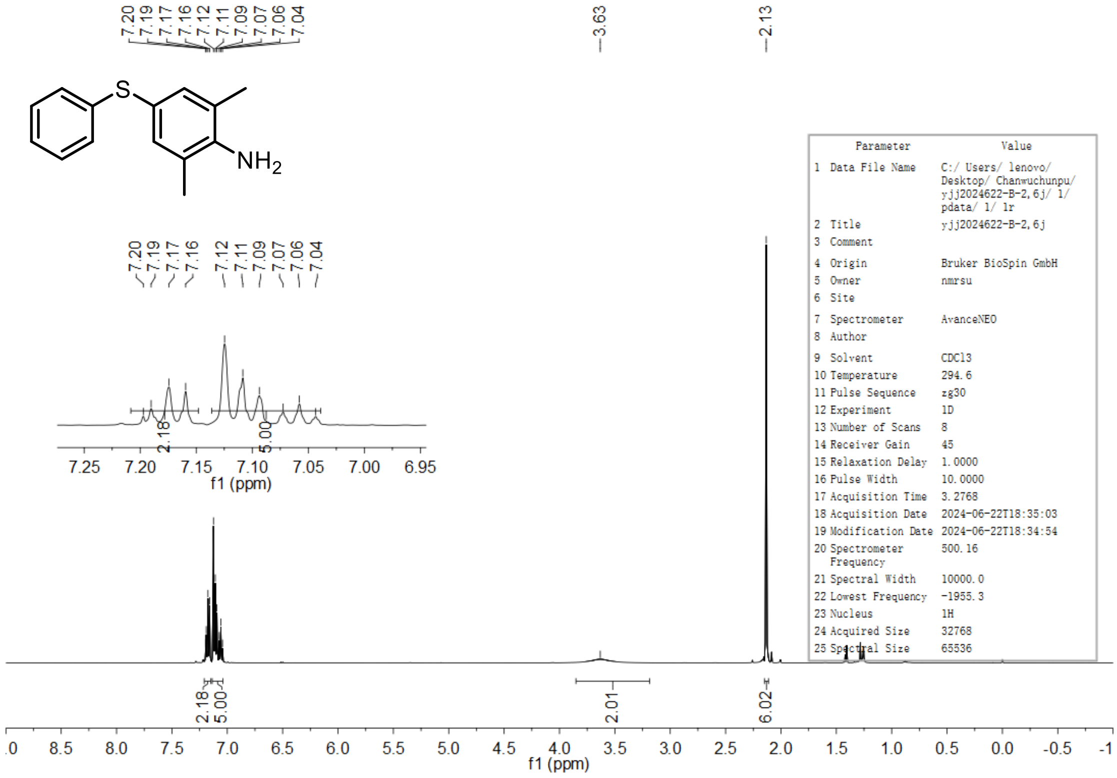

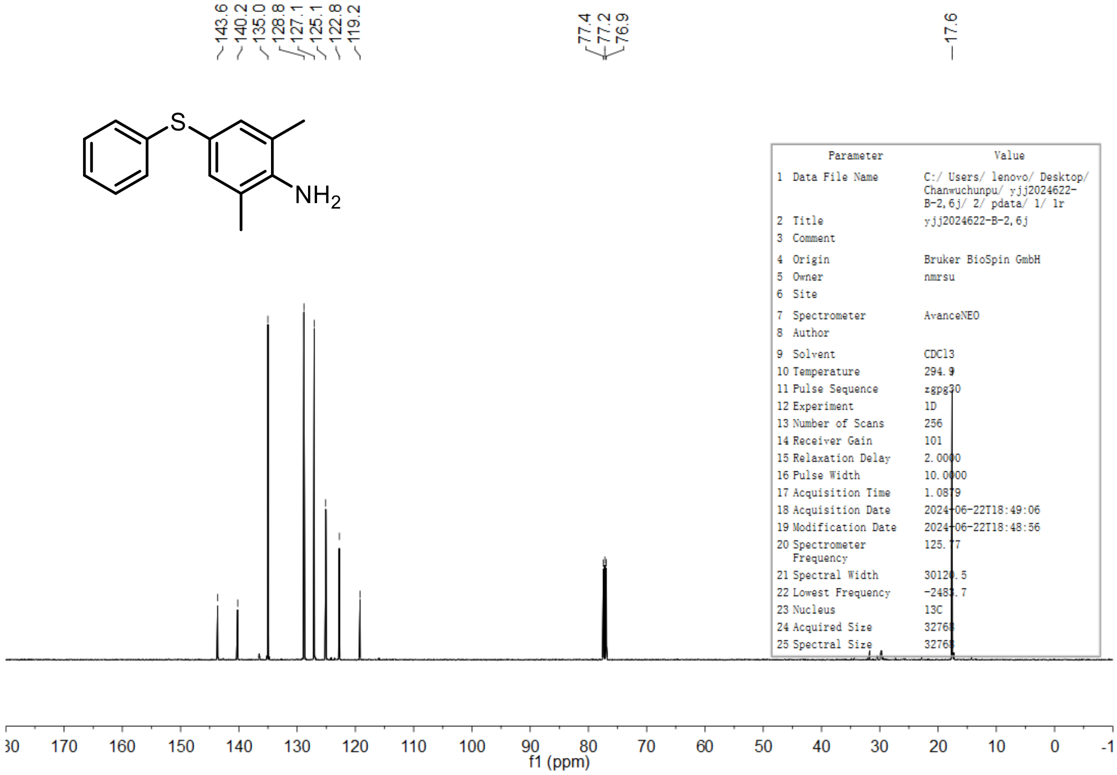

3,5-dimethyl-4-(phenylthio)aniline (**3am**)

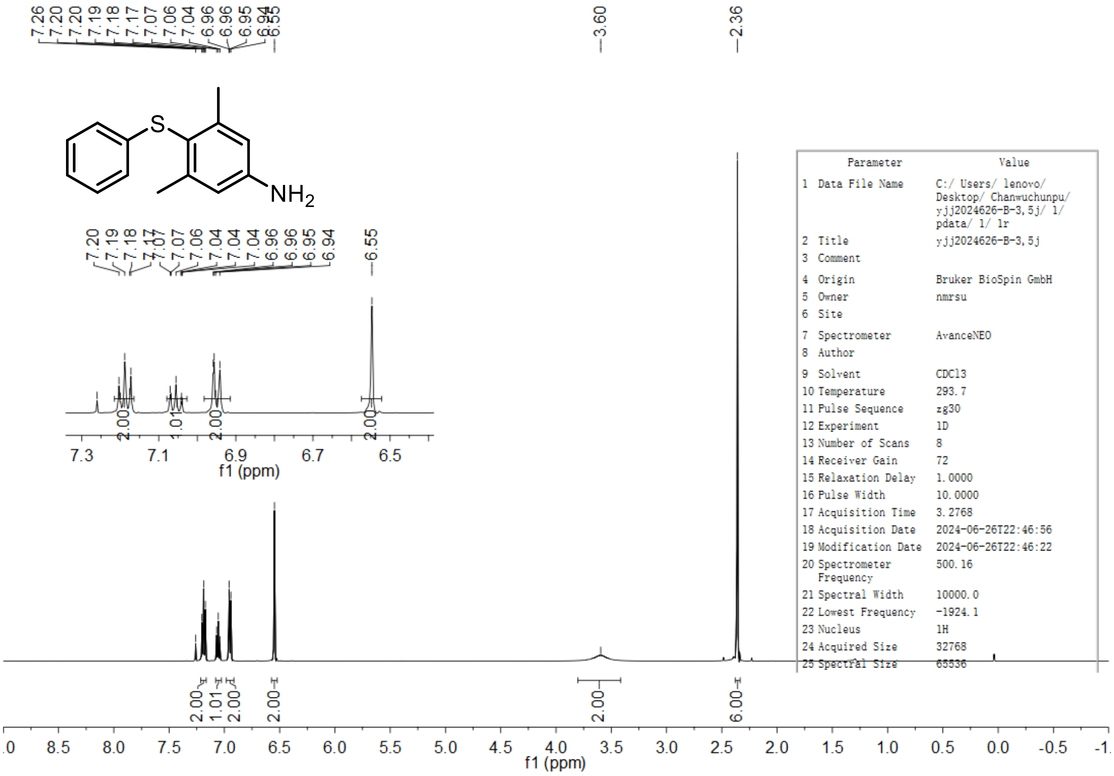

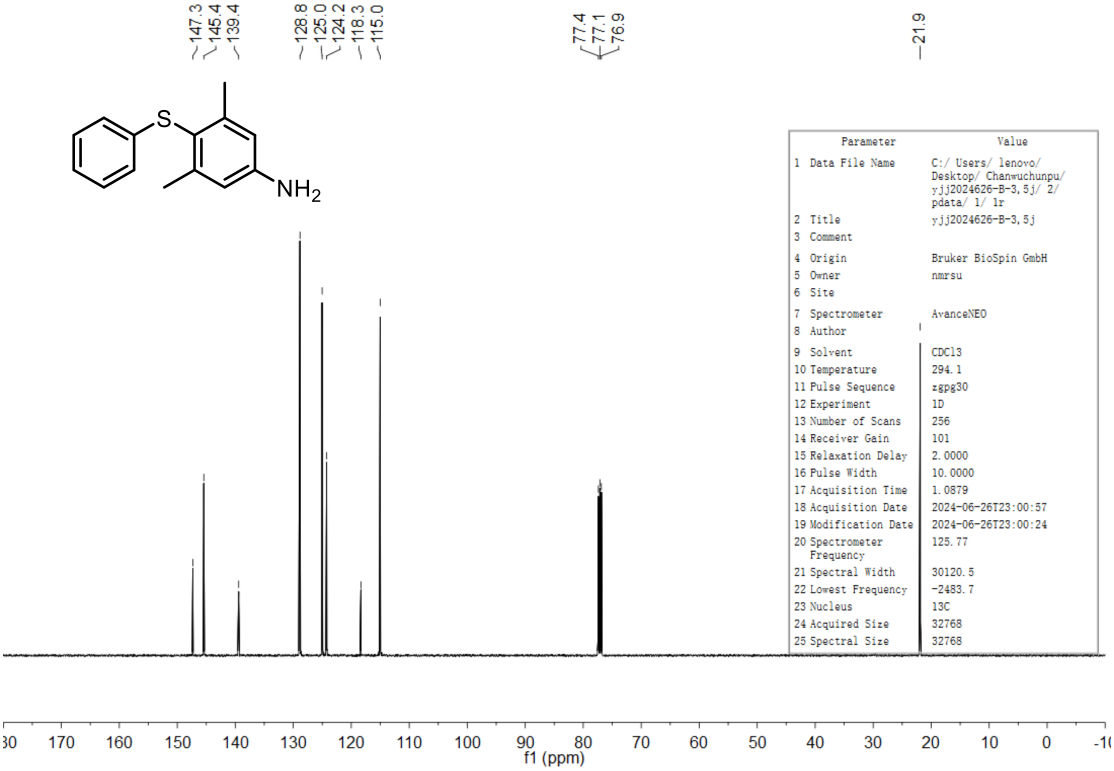

N-methyl-4-(phenylthio)aniline (**3an**)

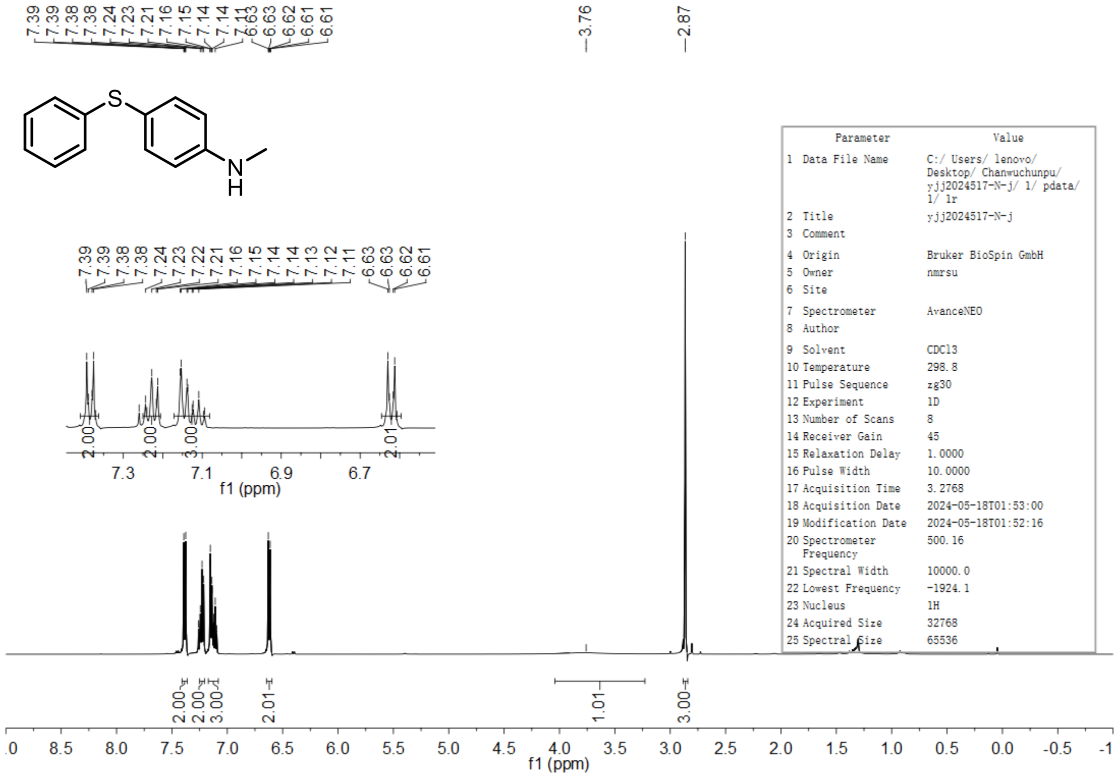

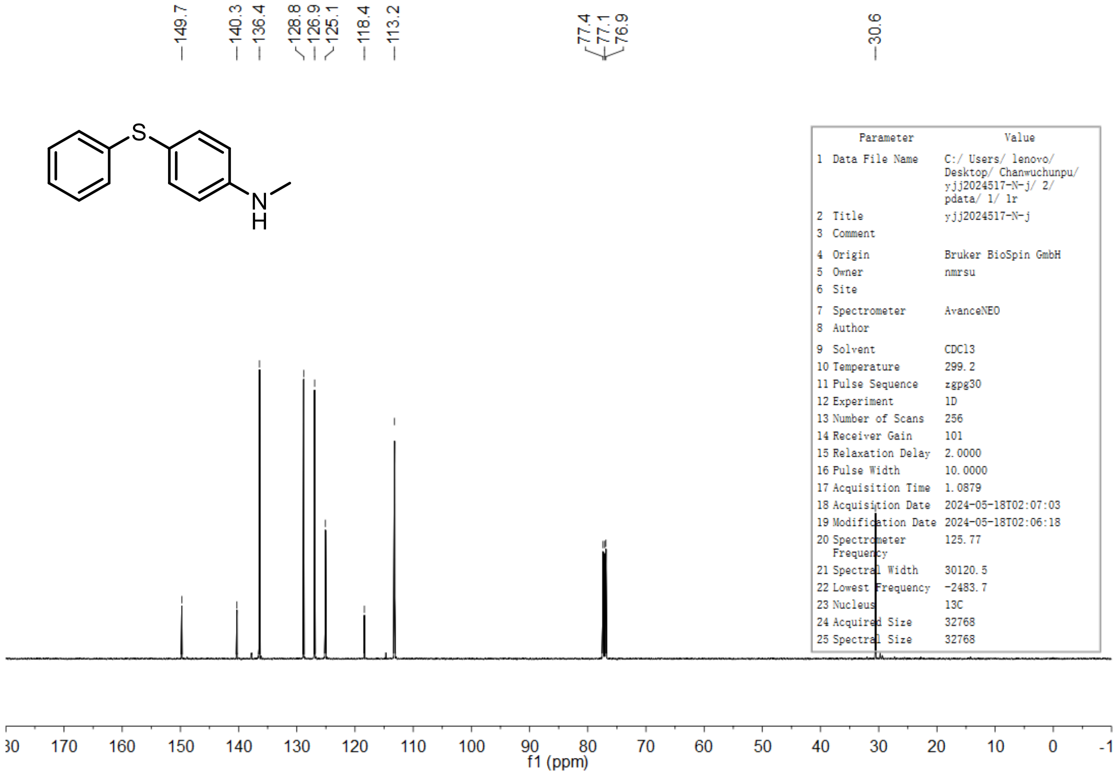

N,N-dimethyl-4-(phenylthio)aniline (**3ao**)

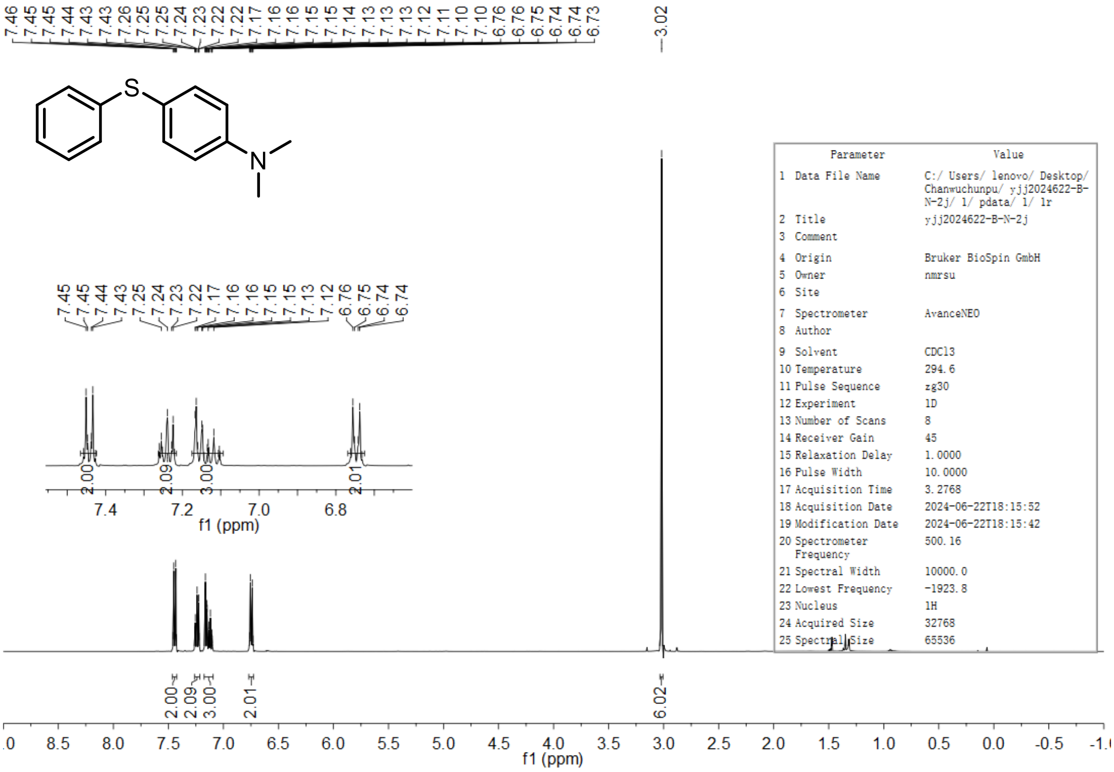

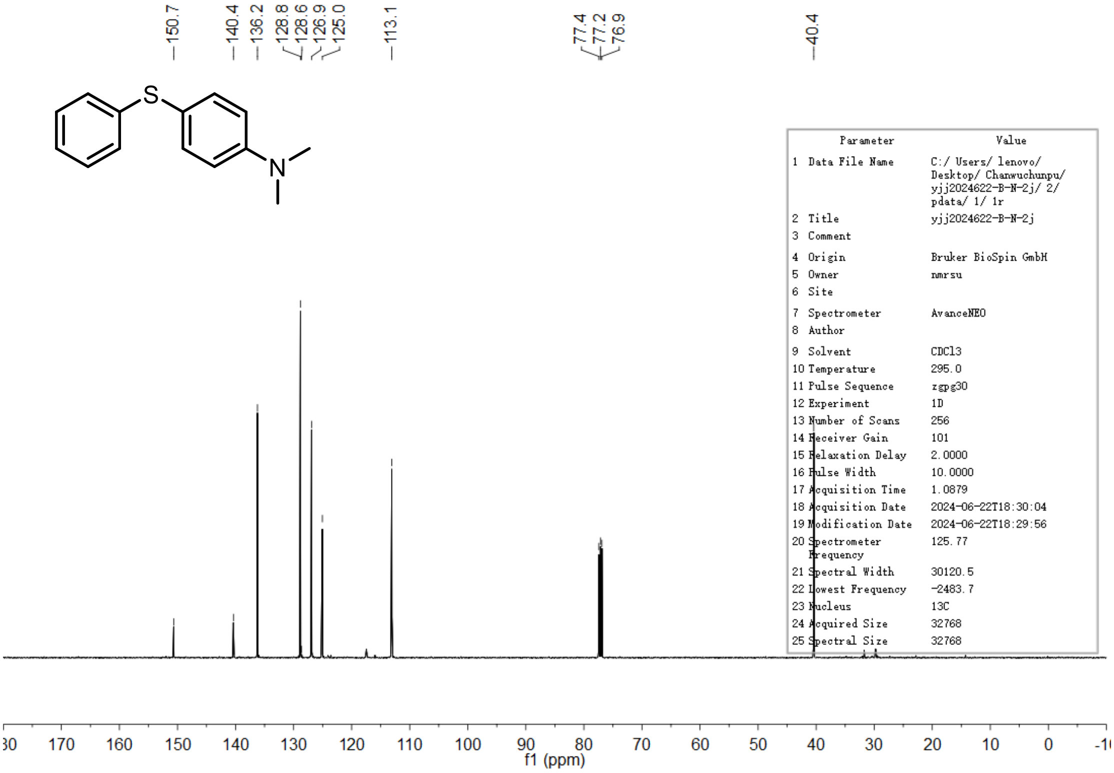

4-(4-(phenylthio)phenyl)morpholine (**3ap**)

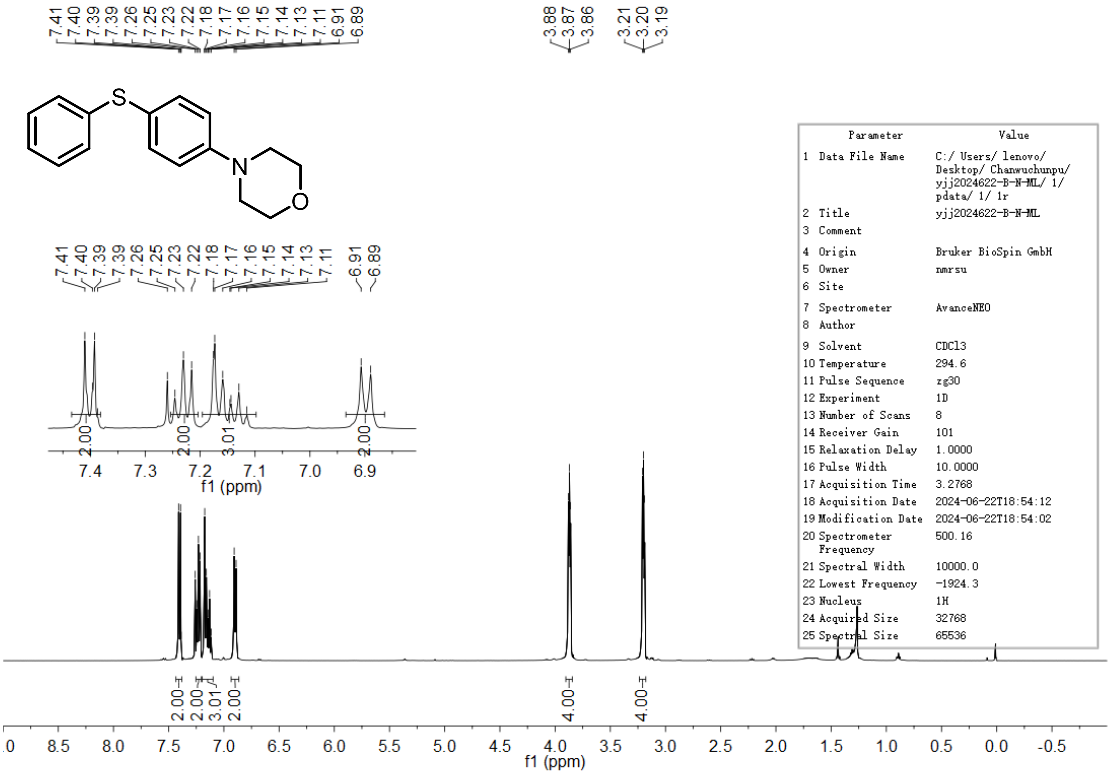

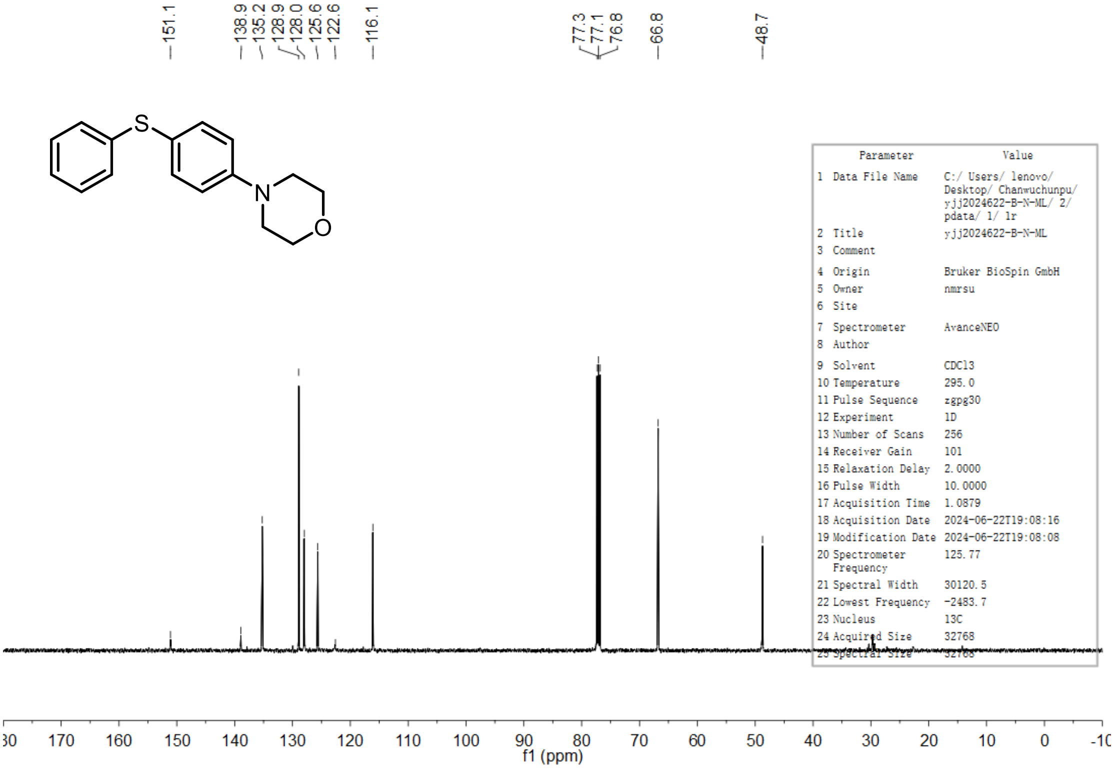

Methyl 2-amino-5-(phenylthio)benzoate (**3aq**)

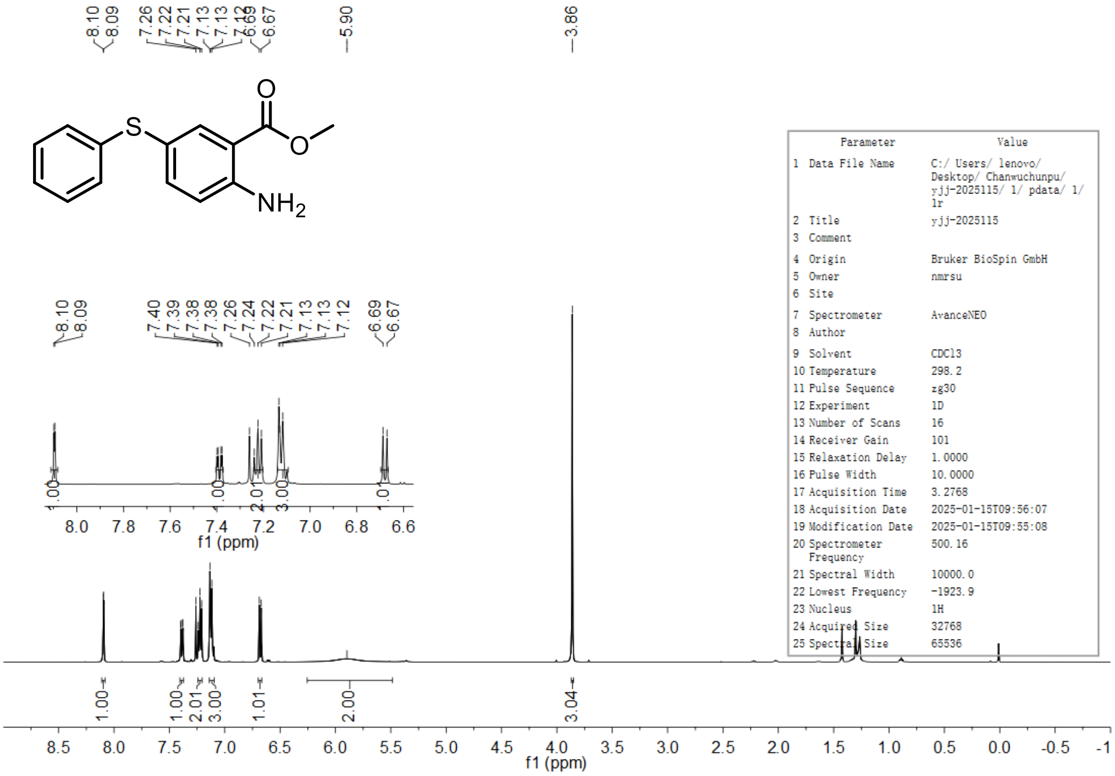

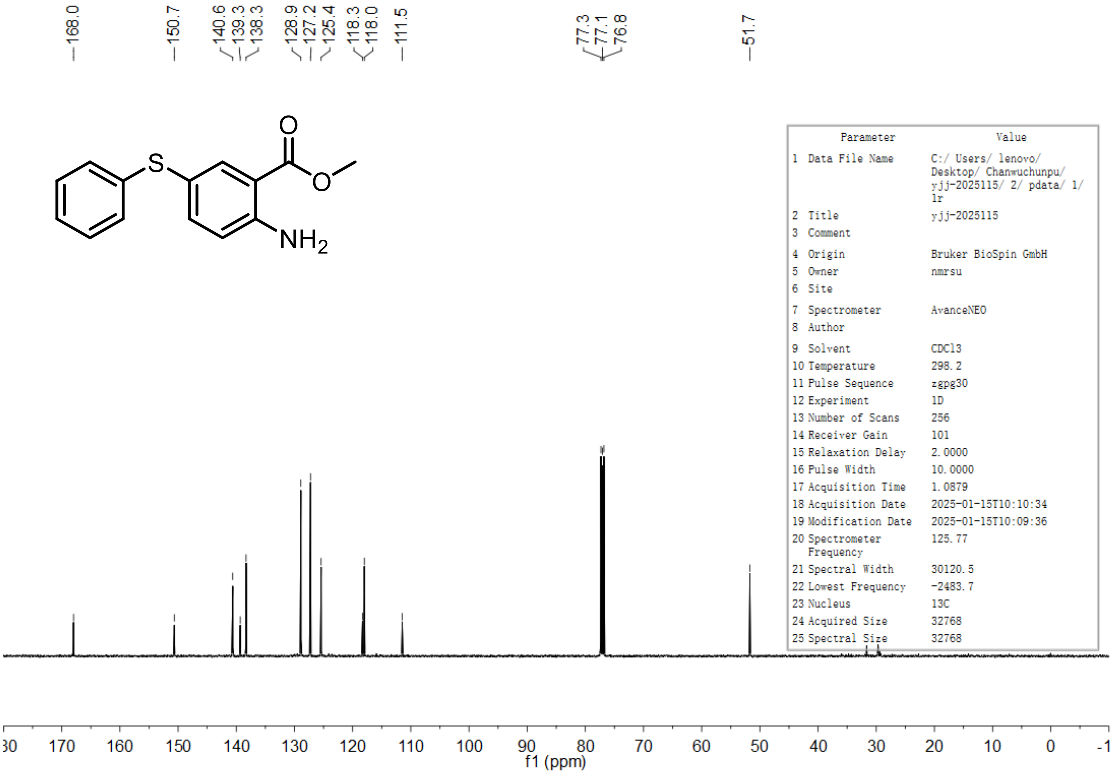

Supplement: RA-015-D5RA00100E-s001 [file RA-015-D5RA00100E-s001.pdf]
